# Adult Hodgkin lymphoma incidence trends in the United States from 2000 to 2020

**DOI:** 10.1038/s41598-024-69975-3

**Published:** 2024-09-03

**Authors:** Armin Aslani, Soroush Morsali, Seyed Ehsan Mousavi, Samireh Choupani, Zahra Yekta, Seyed Aria Nejadghaderi

**Affiliations:** 1 Department of Community Medicine, Faculty of Medicine, Social Determinants of Health Research Center, Tabriz University of Medical Sciences, Tabriz, Iran; 2 Student Research Committee, Tabriz University of Medical Sciences, Tabriz, Iran; 3 Tabriz USERN Office, Universal Scientific Education and Research Network (USERN), Tabriz, Iran; 4 Neurosciences Research Center, Aging Research Institute, Tabriz University of Medical Sciences, Tabriz, Iran; 5 Tabriz University of Medical Sciences, Tabriz, Iran; 6 Calaveras County Department of Health, Calaveras County, CA USA; 7 HIV/STI Surveillance Research Center, and WHO Collaborating Center for HIV Surveillance, Institute for Futures Studies in Health, Kerman University of Medical Sciences, Kerman, Iran; 8 Systematic Review and Meta‑analysis Expert Group (SRMEG), Universal Scientific Education and Research Network (USERN), Tehran, Iran

**Keywords:** Hodgkin’s lymphoma, Surveillance, Epidemiology and End Result, SEER, Incidence, United States, Epidemiology, Cancer epidemiology, Hodgkin lymphoma

## Abstract

Hodgkin lymphoma (HL) is a rare malignancy affecting the lymphatic system. Our study examined the incidence rates of adult HL based on sex, race/ethnicity, age, and histological subgroups in the United States (US) from 2000 to 2020. Data for this study were extracted from the Surveillance, Epidemiology, and End Results 22 database. HL patients were identified utilizing the International Classification of Diseases for Oncology version 3 and categorized as classical HL, lymphocyte-rich/mixed cell/lymphocyte depleted, nodular sclerosis, classical HL, not otherwise specified, and nodular lymphocyte-predominant HL. The study reported average annual percent change (AAPC). All estimates were presented as counts and age-standardized incidence rates (ASIRs) per 100,000 individuals. Between 2000 and 2019, a total of 70,924 cases of HL were reported in the US. Classical HL was the predominant subtype (94.27%), and most incident cases were among non-Hispanic Whites (66.92%) and those aged 20–29 years (24.86%). The ASIR per 100,000 population was 3.83 for men and 2.92 for women. Both sexes showed declines in the AAPCs between 2000 and 2019 (− 0.64% [− 0.99, − 0.28] and − 0.40% [− 0.77, − 0.03] for men and women, respectively). There was a significant decrease in ASIRs after COVID-19 among both sexes (percent change: − 7.49% [− 11.58, − 3.40]). Throughout all age groups, men had a higher incidence rate compared to women, except for those aged 20–29 years. Although the overall HL incidence rate was lowered in the study period from 2000 to 2019, a dramatic decrease in ASIRs of HL patients following COVID-19 pandemic was observed.

## Introduction

Hodgkin lymphoma (HL) is an uncommon monoclonal lymphoid neoplasm with B-cell origin, typically manifested by localized nodal diseases^1,2^. HL is frequently observed in cervical lymph nodes, and it can be categorized into classical HL (CHL) and nodular lymphocyte-predominant HL (NLPHL)^3^. These two disease entities exhibit variations in both clinical presentation and pathology. In this regard, CHL represents around 95% of all cases of HLs and is categorized into four subgroups: nodular sclerosis HL (NSHL), lymphocyte-rich HL, mixed cellularity, and lymphocyte-depleted^4^. HLs are characterized by four key features, which are frequent occurrence in cervical lymph nodes, higher prevalence in young adults, the presence of scattered significant mononuclear Hodgkin and multinucleated cells (Reed-Sternberg) within a background of various non-neoplastic inflammatory cells, and the typical observation of T lymphocytes surrounding the neoplastic cells^2,4^.

Globally, HL accounted for 0.4% of newly reported cancer cases and 0.2% of cancer-related deaths in 2020^5^. In the United States (US), HL has an estimated incidence rate of 0.26 cases per 100,000 individuals in 2020^5^, making up 10% of all diagnosed lymphomas in the country^6^. This disease exhibits a bimodal distribution, with a higher incidence observed in young adults and individuals aged 55 years and older^7^. The prevalence of HL is influenced by factors such as sex, age, geographical location, and various environmental risk factors^8^. Individuals at a heightened risk for HL encompass males^9^, adolescents and young adults^1^, those with a history of Epstein-Barr virus (EBV) infection^10^, individuals with human immunodeficiency virus (HIV)/acquired immunodeficiency syndrome (AIDS)^11^, those with autoimmune diseases^12^, cigarette smokers^13^, and individuals with a family history of HL. Furthermore, research indicates variations in HL incidence based on family size and socioeconomic status^1^.

The impact of the COVID-19 pandemic on HL was significant, marked by a global decrease in the incidence rate of HL by 7.2%^14^. The prolonged interval between symptom onset and diagnosis observed during this period is indicative of the pandemic’s impact on HL, potentially leading to a higher proportion of patients presenting with advanced-stage disease^15^.

Prior studies on HL incidence have either been conducted on a global scale^1^ or have been limited to specific subtypes of hematological cancers, such as Burkitt lymphoma, which is a subtype of non-HL^16^. However, a more precise examination of the updated trends in HL incidence is necessary for resource allocation and public health planning. Consequently, our objective was to perform a comprehensive analysis of the Surveillance, Epidemiology, and End Results (SEER) database to report the incidence trends of adult (≥ 20 years old) HL from 2000 to 2020 in the US by age, sex, race/ethnicity, and histological subgroups. Additionally, we explored the impact of the COVID-19 pandemic on the overall incidence trends of HL in adults.

## Methods

### Data source

The SEER Program functions as a comprehensive, population-based cancer data repository within the US. SEER 22 covers approximately 48% of the US population and furnishes information on patient survival rates and cancer stage at initial diagnosis^17^. The SEER program collects data on patients' demographic characteristics, initial tumor location, tumor morphology, stage at diagnosis, initial treatment, and ongoing monitoring of vital status^17^. In this study, the SEER 22 database, accessible since April 2023 and comprising data reported up to November 2022, was utilized to calculate the incidence rates and annual percent changes (APCs) of HL from 2000 to 2020^18,19^. Access to the SEER 22 database was conducted following the SEER Research Data Agreement for 1975–2020 Data^20^, and cancer statistics were published following the SEER 22 guideline^21^.

### Definitions

Cancer cases are presented in frequencies and percentages, with the incidence rate reported as cases per 100,000 people. The APCs over a specified time period demonstrate variation as a constant proportion of the rate observed in the previous year. The average annual percent changes (AAPCs) represent the mean of multiple APCs during a specific time frame. Patients were classified into three groups: Non-Hispanic White (NHW), Non-Hispanic Black (NHB), and Hispanic. However, due to the limited number of cases, the race and ethnicity groups of American Indian/Alaska Native, Native Hawaiian, and Asian/Pacific Islander were only utilized for calculating parameters related to all races and ethnicities. HL patients were identified using the International Classification of Diseases for Oncology version 3. The morphologies were categorized as follows: 1(a) CHL (codes 9650-9655, 9661-9667), with subtypes 1(a)1 lymphocyte-rich/mixed cell/lymphocyte depleted (codes 9651-9655), 1(a)2 nodular sclerosis (codes 9663-9667), 1(a)3 CHL, not otherwise specified (CHL-NOS) (codes 9650, 9661-9662), and 1(b) NLPHL (code 9659).

### Statistical analysis

The study made use of the SEER 22 Research Limited-Field Data with Delay-Adjustment database spanning the years 2000 to 2020^18^, which was obtained from SEER*Stat, version 8.4.1.2^22^. To compute the age-standardized incidence rate (ASIR) of HL with delay adjustments, the study employed the SEER 22 Research Limited-Field Data with Delay-Adjustment database for the period from 2000 to 2020. The modeling reporting delay was meant to accommodate anticipated future data revisions, encompassing additions and deletions^23^. The adjusted counts and the associated delay model can be utilized to identify current trends in cancer incidence more accurately^23^. The inclusion criteria for cases were limited to individuals diagnosed with cancer whose age at diagnosis was known. Following this, the delay model was implemented, incorporating adjustment factors for variables such as cancer site, registry, age group, race and ethnicity, and year of diagnosis^4,24^. Moreover, the SEER 22 Research Limited-Field Data database spanning the years 2000 to 2020 was employed to ascertain the ASIRs of HL subtypes^18^, which was retrieved from SEER*Stat, version 8.4.1.2^25^. The Tiwari technique was applied to estimate the ASIRs using the 2000 US standard population, along with the corresponding 95% confidence interval (CI)^26^ using the SEER*Stat version 8.4.1.2^22^.

To estimate the APCs, AAPCs^27^, joinpoint regression modeling, parallelism test, and coincident test^28^ for ASIRs^29^, the Joinpoint Regression Program, version 5.0.2^30^ was used. Recognizing the potential bias introduced by the 2020 incidence data due to COVID-19, it was excluded from Joinpoint trends and only represented in graphs.

To compute the APCs of ASIRs for HL, the best fit of least-squares regression lines on the natural logarithm of the rates, with the year of diagnosis as a regressor variable, was employed. A minimum of two observations between two joinpoints and a minimum of two observations from a joinpoint to either end of the data were set as criteria. The selection of models was guided by the weighted Bayesian Information Criteria approach^31^. The 95% CIs for the AAPCs were calculated using the empirical quantile method^32^. The parallelism test was used in pairwise comparisons to assess whether the trends of the two groups exhibited similarity over time^28^. A pairwise comparison employing the coincidence test was also conducted to ascertain whether the rates of the two groups were identical over time.

## Results

### Hodgkin lymphoma

#### Overall incidence

**Figure 1:**
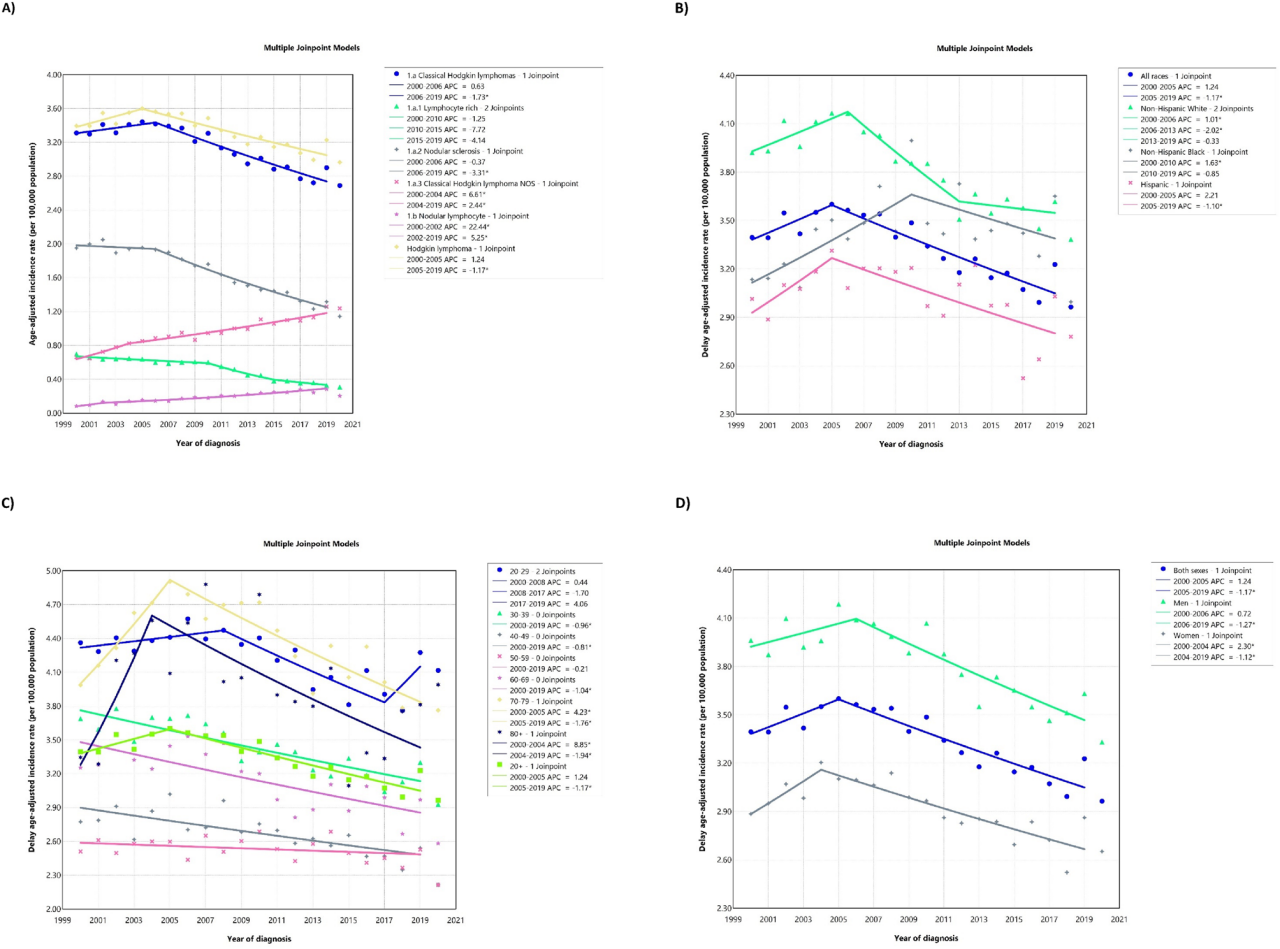
Delayed age-adjusted incidence rate of adult Hodgkin lymphoma over 2000–2019 and in 2020 in the United States, by cancer subtype (**A**), race/ethnicity (**B**), age (**C**), and sex (**D**). APC: annual percent change. * Represent p-value less than 0.05.

Between 2000 and 2019, there were a total of 70,924 cases of adult HL in the US. CHL was the most commonly reported subtype (94.27%). Most of these cases were identified among NHWs (66.92%) and individuals aged 20–29 years (24.86%). The ASIR per 100,000 population was 3.83 (3.79, 3.87) for men and 2.92 (2.88, 2.95) for women. Both sexes experienced a significant decline in AAPCs between 2000 and 2019 at − 0.64% (− 0.99, − 0.28) and − 0.40% (− 0.77, − 0.03) for men and women, respectively. NHW men had the highest ASIR (4.31 [4.26, 4.37]). Among all races and ethnicities, only NHB women exhibited the most significant increase in ASIRs over the 2000–2019 period, with an AAPC of 0.68% (0.08, 1.32) (Table 1 and Fig. 1A–D).

**Table 1:** Counts and age-standardized rate of Hodgkin lymphoma cancer incidence per 100,000 and average annual percent change from 2000 to 2019 in the United States by age, sex, and race.

All race/ethnicities						
Age group (years)	Men			Women		
	Case (%)	Delayed ASIR (95% CI)	AAPC (95% CI)	Case (%)	Delayed ASIR (95% CI)	AAPC (95% CI)
20+	39,149 (55.20)	3.83 (3.79, 3.87)	− 0.64 (− 0.99, − 0.28)	31,775 (44.80)	2.92 (2.88, 2.95)	− 0.4 (− 0.77, − 0.03)
20–29	8792 (12.40)	4.13 (4.05, 4.22)	− 0.48 (− 1.01, 0.07)	8835 (12.46)	4.32 (4.23, 4.41)	− 0.58 (− 1.04, − 0.08)
30–39	7663 (10.80)	3.72 (3.64, 3.8)	− 1.11 (− 1.62, − 0.62)	6511 (9.18)	3.16 (3.08, 3.24)	− 0.75 (− 1.22, − 0.3)
40–49	6673 (9.41)	3.28 (3.2, 3.36)	− 1.23 (− 2.01, − 0.52)	4371 (6.16)	2.11 (2.05, 2.18)	− 0.25 (− 0.96, 0.45)
50–59	5920 (8.35)	3.26 (3.18, 3.35)	− 0.14 (− 0.6, 0.34)	3527 (4.97)	1.85 (1.79, 1.91)	− 0.39 (− 0.94, 0.19)
60–69	4669 (6.58)	3.84 (3.73, 3.95)	− 1.22 (− 1.98, − 0.37)	3357 (4.73)	2.48 (2.39, 2.56)	− 0.87 (− 1.5, − 0.19)
70–79	3598 (5.07)	5.22 (5.05, 5.4)	− 0.06 (− 0.99, 1.21)	3157 (4.45)	3.62 (3.5, 3.75)	− 0.58 (− 2.29, 0.94)
80+	1834 (2.59)	5.11 (4.88, 5.35)	0.67 (− 1.43, 3.17)	2017 (2.84)	3.27 (3.12, 3.41)	− 0.41 (− 2.08, 1.06)

Hispanic						
Age groups	Men			Women		
	Case (%)	Delayed ASIR (95% CI)	AAPC (95% CI)	Case (%)	Delayed ASIR (95% CI)	AAPC (95% CI)
20+	6576 (55.99)	3.58 (3.48, 3.68)	− 0.72 (− 1.18, − 0.21)	5169 (44.01)	2.53 (2.46, 2.61)	0.18 (− 0.97, 1.38)
20–29	1511 (12.87)	2.45 (2.33, 2.58)	1.75 (0.64, 3.15)	1616 (13.76)	2.91 (2.77, 3.06)	0.35 (− 0.65, 1.44)
30–39	1371 (11.67)	2.48 (2.35, 2.62)	− 0.5 (− 1.63, 0.68)	1034 (8.80)	1.98 (1.86, 2.11)	0.5 (− 0.51, 1.62)
40–49	1171 (9.97)	2.68 (2.53, 2.84)	− 1.49 (− 2.85, − 0.05)	664 (5.65)	1.55 (1.44, 1.67)	− 0.2 (− 2.19, 2.09)
50–59	963 (8.20)	3.32 (3.12, 3.54)	− 0.94 (− 2.26, 0.61)	558 (4.75)	1.84 (1.69, 2)	− 1.8 (− 3.56, 0.2)
60–69	728 (6.20)	4.78 (4.44, 5.14)	− 1.57 (− 3.14, 0.49)	555 (4.73)	3.13 (2.88, 3.4)	− 0.78 (− 2.54, 1.37)
70–79	597 (5.08)	8.2 (7.55, 8.89)	− 0.28 (− 1.92, 1.71)	485 (4.13)	4.99 (4.55, 5.45)	0.67 (− 2.26, 4.83)
80+	235 (2.00)	7.19 (6.3, 8.17)	− 1.76 (− 3.51, 0.38)	257 (2.19)	4.77 (4.21, 5.39)	− 1.05 (− 3.11, 1.47)

NHB						
Age groups	Men			Women		
	Case (%)	Delayed ASIR (95% CI)	AAPC (95% CI)	Case (%)	Delayed ASIR (95% CI)	AAPC (95% CI)
20+	4501 (54.30)	4.05 (3.93, 4.18)	0.17 (− 0.58, 0.97)	3788 (45.70)	2.94 (2.84, 3.03)	0.68 (0.08, 1.32)
20–29	1042 (12.57)	3.96 (3.72, 4.21)	0.09 (− 1.67, 2.03)	1004 (12.11)	3.7 (3.48, 3.94)	− 1.11 (− 2.49, 0.28)
30–39	1027 (12.39)	4.45 (4.18, 4.73)	− 0.15 (− 1.39, 1.11)	899 (10.85)	3.49 (3.26, 3.72)	1.12 (− 0.11, 2.44)
40–49	1012 (12.21)	4.47 (4.2, 4.75)	0.22 (− 0.86, 1.31)	678 (8.18)	2.66 (2.46, 2.87)	1.59 (− 0.31, 3.65)
50–59	782 (9.43)	4.16 (3.88, 4.46)	0.7 (− 0.4, 1.96)	544 (6.56)	2.48 (2.28, 2.7)	1.06 (− 0.03, 2.3)
60–69	391 (4.72)	3.48 (3.14, 3.84)	0.31 (− 1.97, 3.11)	373 (4.50)	2.59 (2.34, 2.87)	− 1.03 (− 3.29, 3)
70–79	181 (2.18)	3.34 (2.87, 3.87)	− 0.09 (− 2.07, 2.19)	200 (2.41)	2.44 (2.11, 2.8)	1.78 (− 0.11, 4.13)
80+	66 (0.80)	2.85 (2.2, 3.63)	N/A	90 (1.09)	1.88 (1.51, 2.32)	1.51 (− 8.92, 13.54)

NHW						
Age groups	Men			Women		
	Case (%)	Delayed ASIR (95% CI)	AAPC (95% CI)	Case (%)	Delayed ASIR (95% CI)	AAPC (95% CI)
20+	26,182 (55.16)	4.31 (4.26, 4.37)	− 0.72 (− 1.43, − 0.16)	21,283 (44.84)	3.4 (3.36, 3.45)	− 0.56 (− 1.16, 0.01)
20–29	5662 (11.93)	5.4 (5.26, 5.54)	− 0.74 (− 1.42, − 0.2)	5624 (11.85)	5.53 (5.39, 5.68)	− 0.56 (− 1.47, 0.18)
30–39	4870 (10.26)	4.53 (4.4, 4.66)	− 1.09 (− 1.65, − 0.57)	4224 (8.90)	3.99 (3.87, 4.11)	− 1 (− 1.55, − 0.48)
40–49	4229 (8.91)	3.56 (3.45, 3.67)	− 1 (− 1.54, − 0.5)	2854 (6.01)	2.42 (2.33, 2.51)	− 0.17 (− 1.01, 0.6)
50–59	3914 (8.25)	3.28 (3.18, 3.39)	− 0.1 (− 0.53, 0.33)	2297 (4.84)	1.88 (1.81, 1.96)	− 0.38 (− 1.06, 0.3)
60–69	3354 (7.07)	3.91 (3.78, 4.05)	− 1.36 (− 2.3, − 0.34)	2318 (4.88)	2.51 (2.41, 2.61)	− 1.18 (− 2.03, − 0.28)
70–79	2692 (5.67)	5.25 (5.06, 5.46)	− 0.03 (− 0.67, 0.82)	2359 (4.97)	3.75 (3.6, 3.9)	− 1.11 (− 2.24, 0.01)
80+	1461 (3.08)	5.22 (4.96, 5.5)	0.66 (− 1.8, 3.43)	1607 (3.39)	3.36 (3.19, 3.53)	− 1.15 (− 3.06, 0.64)

NHW, Non-Hispanic White; NHB, Non-Hispanic Black; ASIR, age-standardized incidence rate; CI, confidence interval; AAPC, average annual percent change.

In the more recent period from 2015 to 2019, the US reported a total of 17,903 cases of HL. Most cases were observed among NHWs (62.33%), and the highest incidence occurred in individuals aged 20–29 years (24.47%). During this period, the ASIRs per 100,000 population was 3.56 (3.49, 3.63) for men and 2.73 (2.67, 2.79) for women. There was a significant decrease in ASIRs over the 2015–2019 period in both men and women, with an AAPC of − 1.27% (− 2.20, − 0.92) and − 1.11% (− 1.75, − 0.80), respectively (Table S1).

The findings of the identical and parallel trends are provided in Tables S2 and S3, respectively.

##### Men

From 2000 to 2019, 39,149 (55.20%) cases of HL were diagnosed in men. The majority of cases were in individuals aged 20–29 years (62.33%) and NHWs (66.88%). Cases between 70 to 79 years had the highest ASIR among all age groups (5.22 [5.05, 5.40]). Individuals between 40 and 49 years showed the highest decrease from 2000 to 2019 (AAPC: − 1.23% [− 2.01, − 0.52]) (Table 1).

Of all the reported cases, 16.80% were among Hispanic men. The majority of cases fell within the age range of 20–29 years (22.98%). The overall delayed ASIR per 100,000 population was 3.58 (3.48, 3.68), with cases between 70 and 79 years having the highest delayed ASIR at 8.20 (7.55, 8.89). The overall AAPC for this group was − 0.72% (− 1.18, − 0.21). Among age groups, those in the 20–29 age group had a significant increase in ASIR with an AAPC of 1.75% (0.64, 3.15), and individuals in the 40–49 age group had the largest decrease in ASIRs (AAPC: − 1.49% [− 2.85, − 0.05]) (Table 1).

The majority of NHB men cases (45.58%) occurred in individuals aged 20–29 years. The delayed ASIR per 100,000 population was 4.05 (3.93, 4.18), with those aged 40–49 years exhibiting the highest ASIR at 4.47 (4.20, 4.75) (Table 1).

The majority of NHW men fell within the 55–69 years age bracket (21.63%). The overall delayed ASIR per 100,000 population was 4.31 (4.26, 4.37) (Table 1).

##### Women

A total of 31,775 cases of HL were reported among women in the US during the period 2000–2019. The majority of cases were observed in NHWs (66.89%) and within the age range of 20 to 29 years (27.80%). Cases between 20 and 29 years exhibited the highest delayed ASIR among all age groups (4.32 [4.23, 4.41]). Individuals between 60 and 69 years experienced the largest decrease in ASIRs from 2000 to 2019 (AAPC: − 0.87% [− 1.50, − 0.19]). NHB women were the only group to show a significant increase in ASIR from 2000 to 2019, with an AAPC of 0.68% (0.08, 1.32) (Table 1).

Among all women, 16.27% were Hispanics. Most cases were observed in the age group of 20–29 years (31.26%). The delayed ASIR per 100,000 population in Hispanic women was 2.53 (2.46, 2.61), with individuals between 70 and 79 years had the highest ASIR (4.99 [4.55, 5.45]) (Table 1).

Among women, 11.92% of reported cases were NHBs. The majority of cases (26.50%) were in the age group of 20–29 years. The delayed ASIR per 100,000 population in NHB women was 2.94 (2.84, 3.03). Cases between 30 and 39 years had the highest reported ASIR (3.49 [3.26, 3.72]) (Table 1).

The majority of NHW cases occurred in women aged 20–29 years (26.42%). The delayed ASIR per 100,000 population in NHW women was 3.40 (3.36, 3.45). Individuals between 20 and 29 years had the highest reported ASIR (5.53 [5.39, 5.68]) (Table 1).

#### Age and sex patterns

**Figure 2:**
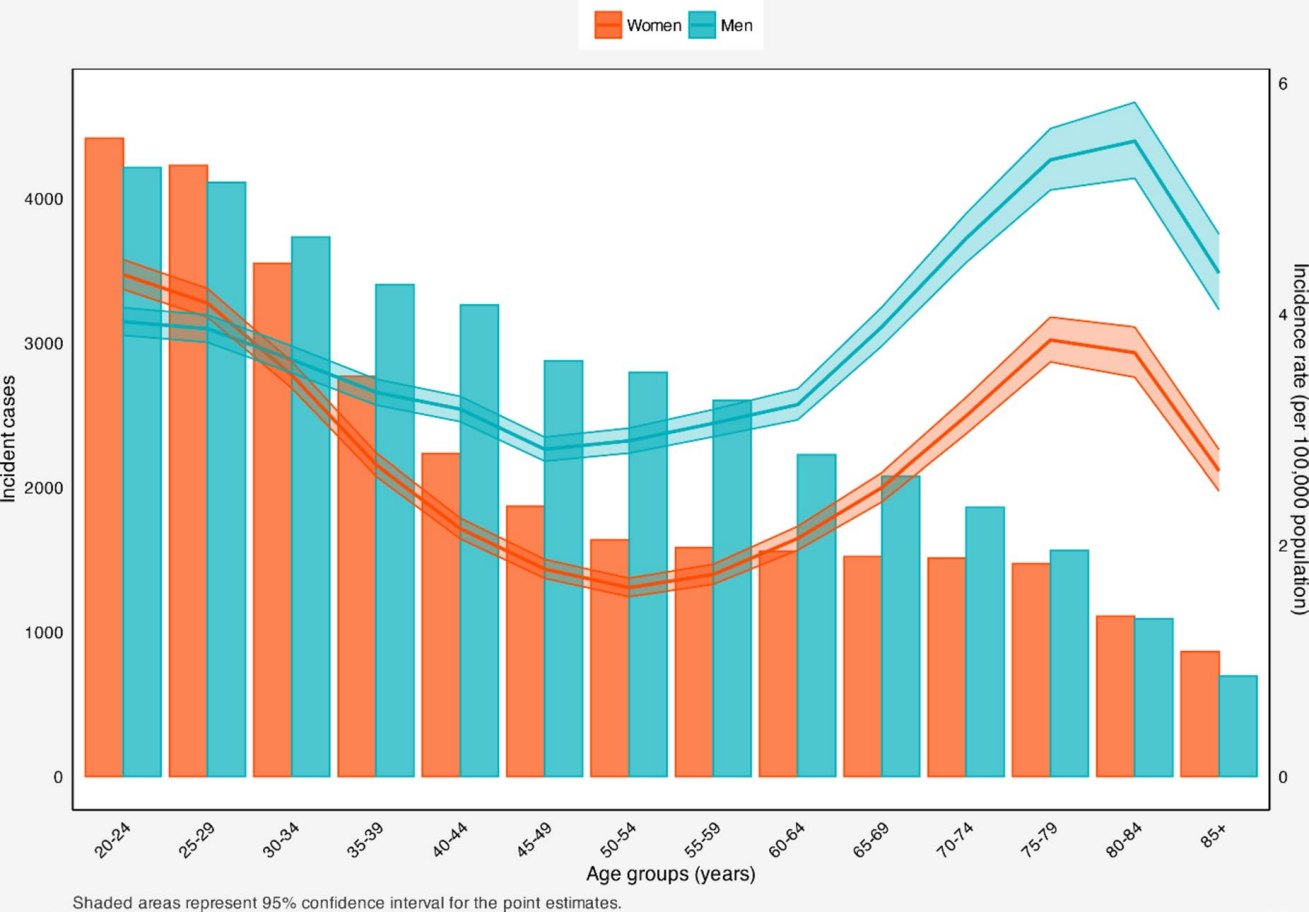
Incident cases and delay-adjusted incidence rate of adult Hodgkin lymphoma in the United States among males and females in each age group.

There was a decrease in incident cases in both sexes from ages 20–24 to 85+ years old. In contrast to the age groups of 20–29 and > 80, where women had a higher incident cases compared to men, in other age groups, men had a higher incident numbers. Concerning the incidence rates, both sexes experienced a steady decline, reaching the minimum at the age range of 45–49 in men and 50–54 in women. This was followed by an increase, peaking at 80–84 for men and 75–79 in women. Throughout all age groups, men had a higher incidence rate compared to women; however, between 20 and 29, women had a higher incidence rates (Fig. 2).

#### COVID-19 impacts

There was a significant decrease in the ASIRs of adult HL across all races/ethnicities in both sexes within all age groups (percent change (PC): − 7.49% [− 11.58, − 3.40]) and for males (PC: − 7.22% [− 12.76, − 1.69]) and females (PC: − 7.25% [− 13.39, − 1.11]) from 2019 to 2020 (Table 2).

**Table 2:** Percent change in age-standardized, delay-adjusted incidence rates of adult Hodgkin lymphoma from 2019 to 2020, by race and sex, using the November 2022 data submission.

Races/ethnicities	Sex	2019 Delayed ASIR (95% CI)	2020 Delayed ASIR (95% CI)	PC (95% CI)
All	Both	2.66 (2.58, 2.74)	2.46 (2.38, 2.54)	− 7.49 (− 11.58, − 3.4)
	Female	2.38 (2.27, 2.49)	2.2 (2.1, 2.31)	− 7.25 (− 13.39, − 1.11)
	Male	2.97 (2.85, 3.09)	2.75 (2.63, 2.87)	− 7.22 (− 12.76, − 1.69)
Hispanic	Both	2.47 (2.31, 2.65)	2.28 (2.13, 2.45)	− 7.7 (− 16.77, 1.37)
	Female	2.08 (1.87, 2.3)	1.89 (1.69, 2.1)	− 9.17 (− 22.57, 4.23)
	Male	2.91 (2.65, 3.19)	2.73 (2.48, 3.01)	− 6.08 (− 18.64, 6.49)
NHB	Both	2.85 (2.61, 3.11)	2.49 (2.26, 2.73)	− 12.83 (− 23.93, − 1.74)
	Female	2.47 (2.16, 2.81)	2.32 (2.02, 2.64)	− 6.24 (− 23.58, 11.11)
	Male	3.29 (2.91, 3.7)	2.76 (2.41, 3.15)	− 16.05 (− 30.91, − 1.18)
NHW	Both	3 (2.88, 3.13)	2.81 (2.69, 2.94)	− 6.26 (− 11.8, − 0.73)
	Female	2.76 (2.6, 2.94)	2.58 (2.41, 2.75)	− 6.73 (− 14.96, 1.51)
	Male	3.26 (3.08, 3.44)	3.08 (2.91, 3.26)	− 5.53 (− 13.01, 1.95)

NHW, Non-Hispanic White; NHB, Non-Hispanic Black; ASIR, age-standardized incidence rate; CI, confidence interval; PC, percent change.

### Classical Hodgkin lymphoma (CHL)

From 2000 to 2019, there were 66,861 CHL cases in all age groups in the US. The majority of cases were men (54.62%), NHWs (67.41%), and between 20 and 29 years (25.38%). The ASIR per 100,000 population was 3.57 (3.53, 3.61) for men and 2.78 (2.75, 2.81) for women. NHW men had the highest ASIR (4.03 [3.98, 4.09]). The AAPCs for men and women were − 1.07% (− 1.43, − 0.69) and − 0.75% (− 1.07, − 0.40), respectively, and NHW men had the largest decrease compared to other groups (AAPC: − 1.14% [− 1.88, − 0.56]) (Table 3, Figs. S1–S3). The details of incidence trends for males and females are provided in Appendix 1.

**Table 3:** Counts and age-standardized rate of classical Hodgkin lymphoma incidence per 100,000 and average annual percent change from 2000 to 2019 in the United States by age, sex, and race.

All race/ethnicities						
Age group (years)	Men			Women		
	Case (%)	ASIR (95% CI)	AAPC (95% CI)	Case (%)	ASIR (95% CI)	AAPC (95% CI)
20+	36,522 (54.62)	3.57 (3.53, 3.61)	− 1.07 (− 1.43, − 0.69)	30,339 (45.38)	2.78 (2.75, 2.81)	− 0.75 (− 1.07, − 0.4)
20–29	8326 (12.45)	3.9 (3.82, 3.99)	− 0.74 (− 1.28, − 0.2)	8646 (12.93)	4.22 (4.13, 4.31)	− 0.76 (− 1.23, − 0.23)
30–39	7138 (10.68)	3.46 (3.38, 3.54)	− 1.56 (− 2.16, − 1)	6322 (9.46)	3.06 (2.98, 3.14)	− 0.95 (− 1.43, − 0.49)
40–49	6139 (9.18)	3.01 (2.94, 3.09)	− 1.97 (− 2.79, − 1.28)	4104 (6.14)	1.98 (1.92, 2.04)	− 0.88 (− 1.58, − 0.22)
50–59	5400 (8.08)	2.97 (2.89, 3.05)	− 0.78 (− 1.35, − 0.18)	3222 (4.82)	1.68 (1.63, 1.74)	− 1.08 (− 1.54, − 0.6)
60–69	4303 (6.44)	3.53 (3.43, 3.64)	− 1.67 (− 2.28, − 1)	3082 (4.61)	2.27 (2.19, 2.35)	− 1.51 (− 2.17, − 0.79)
70–79	3429 (5.13)	4.97 (4.8, 5.14)	− 0.38 (− 1.77, 1.13)	2988 (4.47)	3.42 (3.3, 3.55)	− 0.96 (− 2.26, 0.47)
80+	1787 (2.67)	4.97 (4.74, 5.2)	0.6 (− 2.18, 3.78)	1975 (2.95)	3.19 (3.05, 3.34)	− 0.58 (− 2.16, 0.82)

Hispanic						
Age groups	Men			Women		
	Case (%)	ASIR (95% CI)	AAPC (95% CI)	Case (%)	ASIR (95% CI)	AAPC (95% CI)
20+	6255 (55.61)	3.41 (3.31, 3.51)	− 1 (− 1.49, − 0.45)	4992 (44.39)	2.43 (2.36, 2.5)	− 0.05 (− 1.18, 1.16)
20–29	1427 (12.69)	2.31 (2.19, 2.43)	1.51 (0.07, 3.46)	1593 (14.16)	2.86 (2.72, 3)	0.19 (− 0.82, 1.32)
30–39	1297 (11.53)	2.34 (2.21, 2.47)	− 0.79 (− 1.97, 0.45)	1009 (8.97)	1.92 (1.81, 2.05)	0.31 (− 0.66, 1.37)
40–49	1110 (9.87)	2.53 (2.39, 2.69)	− 2 (− 3.57, − 0.35)	620 (5.51)	1.44 (1.33, 1.56)	− 0.75 (− 2.92, 1.73)
50–59	905 (8.05)	3.11 (2.91, 3.32)	− 1.31 (− 2.99, 0.63)	524 (4.66)	1.72 (1.58, 1.88)	− 2.52 (− 4.23, − 0.63)
60–69	702 (6.24)	4.59 (4.25, 4.94)	− 1.76 (− 3.19, − 0.06)	525 (4.67)	2.95 (2.7, 3.21)	− 0.97 (− 2.87, 1.33)
70–79	584 (5.19)	7.98 (7.34, 8.66)	− 0.46 (− 1.89, 1.28)	469 (4.17)	4.8 (4.38, 5.26)	0.63 (− 3.08, 5.98)
80+	230 (2.04)	7.01 (6.13, 7.98)	− 1.32 (− 2.87, 0.59)	252 (2.24)	4.66 (4.1, 5.27)	− 0.86 (− 2.79, 1.5)

NHB						
Age groups	Men			Women		
	Case (%)	ASIR (95% CI)	AAPC (95% CI)	Case (%)	ASIR (95% CI)	AAPC (95% CI)
20+	4003 (54.81)	3.59 (3.47, 3.7)	− 0.68 (− 2.44, 1.16)	3300 (45.19)	2.54 (2.46, 2.63)	− 0.26 (− 0.98, 0.5)
20–29	960 (13.15)	3.63 (3.4, 3.86)	− 0.56 (− 2.49, 1.54)	940 (12.87)	3.45 (3.23, 3.68)	− 1.61 (− 3.09, − 0.13)
30–39	921 (12.61)	3.97 (3.72, 4.24)	− 0.95 (− 2.29, 0.38)	802 (10.98)	3.1 (2.89, 3.32)	0.64 (− 0.8, 2.18)
40–49	882 (12.08)	3.88 (3.63, 4.14)	− 1.05 (− 2.3, 0.15)	559 (7.65)	2.18 (2, 2.37)	− 0.08 (− 1.63, 1.48)
50–59	679 (9.30)	3.59 (3.33, 3.87)	0 (− 1.21, 1.45)	441 (6.04)	2 (1.82, 2.19)	0.06 (− 1.96, 2.37)
60–69	335 (4.59)	2.96 (2.65, 3.29)	− 0.38 (− 2.85, 2.62)	302 (4.14)	2.08 (1.85, 2.33)	− 1.03 (− 2.99, 1.22)
70–79	163 (2.23)	3 (2.55, 3.5)	− 0.67 (− 3.12, 2.12)	170 (2.33)	2.06 (1.76, 2.39)	0.8 (− 1.22, 3.21)
80+	63 (0.86)	2.71 (2.08, 3.47)	N/A	86 (1.18)	1.79 (1.43, 2.21)	0.86 (− 8.76, 11.92)

NHW						
Age groups	Men			Women		
	Case (%)	ASIR (95% CI)	AAPC (95% CI)	Case (%)	ASIR (95% CI)	AAPC (95% CI)
20+	24,498 (54.35)	4.03 (3.98, 4.09)	− 1.14 (− 1.88, − 0.56)	20,576 (45.65)	3.29 (3.25, 3.34)	− 0.81 (− 1.21, − 0.35)
20–29	5389 (11.96)	5.13 (4.99, 5.27)	− 1.15 (− 1.74, − 0.59)	5532 (12.27)	5.43 (5.28, 5.57)	− 0.69 (− 1.67, 0.08)
30–39	4546 (10.09)	4.22 (4.1, 4.34)	− 1.56 (− 2.25, − 0.92)	4164 (9.24)	3.92 (3.8, 4.04)	− 1.13 (− 1.67, − 0.61)
40–49	3911 (8.68)	3.29 (3.19, 3.39)	− 1.65 (− 2.25, − 1.13)	2759 (6.12)	2.33 (2.25, 2.42)	− 0.55 (− 1.38, 0.22)
50–59	3581 (7.94)	3 (2.9, 3.1)	− 0.74 (− 1.18, − 0.29)	2149 (4.77)	1.76 (1.69, 1.84)	− 0.94 (− 1.57, − 0.32)
60–69	3085 (6.84)	3.59 (3.46, 3.72)	− 1.83 (− 2.94, − 0.65)	2158 (4.79)	2.33 (2.23, 2.43)	− 1.79 (− 2.56, − 0.99)
70–79	2561 (5.68)	4.99 (4.8, 5.19)	− 0.36 (− 1.07, 0.75)	2239 (4.97)	3.55 (3.41, 3.7)	− 1.34 (− 3.09, 0.1)
80+	1425 (3.16)	5.08 (4.82, 5.35)	0.49 (− 2.25, 3.54)	1575 (3.49)	3.28 (3.12, 3.45)	− 0.58 (− 2.67, 1.16)

NHW, non-Hispanic White; NHB, non-Hispanic Black; ASIR, age-standardized incidence rate; CI, confidence interval; AAPC, average annual percent change.

There was a decrease in incident cases of CHL with ageing. Regarding the incidence rates, both sexes experienced a gradual decline, reaching its lowest point in the age range of 45–49 for men and 50–54 for women. Subsequently, there was an increase, peaking at 80–84 for men and 75–79 for women. Across all age groups, men consistently had higher incidence rates than women, except for the 20–29 age range (Fig. S4).

### Lymphocyte-rich/mixed cell/lymphocyte-depleted Hodgkin lymphoma (LR/MC/LD HL)

Over 2000–2019, there were a total of 11,188 cases of LR/MC/LD HL in the US across all age groups. The majority of cases were observed in men (62.65%), NHWs (64.48%), and individuals aged 50–59 years (17.05%). The ASIR per 100,000 population was 0.69 (0.67, 0.71) for men and 0.37 (0.36, 0.38) for women. Hispanic men had the highest ASIR compared to other groups at 0.79 (0.74, 0.84). The AAPC for men and women were − 4.20% (− 4.88, − 3.70) and − 3.50 (− 4.31, − 2.79), respectively. Hispanic men experienced the most significant decline from 2000 to 2019 (AAPC: − 4.48% [− 5.88, − 3.10]) (Table 4, Figs. S5–S7). The details of incidence trends for males and females are provided in Appendix 1.

**Table 4:** Counts and age-standardized rate of lymphocyte-rich/mixed cell/lymphocyte depleted Hodgkin cancer incidence per 100,000 and average annual percent change from 2000 to 2019 in the United States, by age, sex, and race.

All race/ethnicities						
Age group (years)	Men			Women		
	Case (%)	ASIR (95% CI)	AAPC (95% CI)	Case (%)	ASIR (95% CI)	AAPC (95% CI)
20+	7009 (62.65)	0.69 (0.67, 0.71)	− 4.2 (− 4.88, − 3.7)	4179 (37.35)	0.37 (0.36, 0.38)	− 3.5 (− 4.31, − 2.79)
20–29	1049 (9.38)	0.49 (0.46, 0.52)	− 2.87 (− 4.36, − 1.45)	518 (4.63)	0.25 (0.23, 0.28)	− 2.36 (− 4.53, − 0.27)
30–39	1117 (9.98)	0.54 (0.51, 0.58)	− 3.87 (− 7.02, − 1.55)	530 (4.74)	0.26 (0.24, 0.28)	− 2.52 (− 6.3, 0.31)
40–49	1328 (11.87)	0.65 (0.62, 0.69)	− 5.24 (− 8.46, − 3.28)	531 (4.75)	0.25 (0.23, 0.28)	− 3.08 (− 5.36, − 1.06)
50–59	1282 (11.46)	0.7 (0.67, 0.74)	− 3.33 (− 4.33, − 2.39)	625 (5.59)	0.32 (0.3, 0.35)	− 3.37 (− 4.86, − 1.95)
60–69	1031 (9.22)	0.85 (0.8, 0.9)	− 4.67 (− 6.09, − 3.3)	704 (6.29)	0.52 (0.48, 0.56)	− 4.26 (− 5.91, − 2.59)
70–79	800 (7.15)	1.16 (1.08, 1.24)	− 3.77 (− 5.15, − 2.48)	811 (7.25)	0.93 (0.87, 1)	− 3.23 (− 6.2, − 0.85)
80+	402 (3.59)	1.12 (1.01, 1.23)	− 3.63 (− 7.29, − 0.42)	460 (4.11)	0.75 (0.68, 0.82)	-5.66 (− 8.27, − 3.55)

Hispanic						
Age groups	Men			Women		
	Case (%)	ASIR (95% CI)	AAPC (95% CI)	Case (%)	ASIR (95% CI)	AAPC (95% CI)
20+	1398 (64.54)	0.79 (0.74, 0.84)	− 4.48 (− 5.88, − 3.1)	768 (35.46)	0.44 (0.41, 0.47)	− 2.41 (− 5.07, 0.31)
20–29	204 (9.42)	0.33 (0.29, 0.38)	− 1.48 (− 7.35, 3.73)	96 (4.43)	0.17 (0.14, 0.21)	− 1.46 (− 7.02, 5)
30–39	298 (13.76)	0.54 (0.48, 0.6)	− 3.66 (− 7.19, − 0.35)	111 (5.12)	0.21 (0.18, 0.26)	− 4.21 (− 7.94, − 0.73)
40–49	305 (14.08)	0.7 (0.62, 0.78)	− 4.28 (− 6.35, − 2.3)	92 (4.25)	0.21 (0.17, 0.26)	N/A
50–59	232 (10.71)	0.8 (0.7, 0.91)	− 5.3 (− 9.64, − 1.37)	133 (6.14)	0.44 (0.37, 0.52)	− 5.3 (− 8.48, − 2.19)
60–69	178 (8.22)	1.17 (1, 1.36)	− 4.54 (− 7.36, − 1.44)	140 (6.46)	0.79 (0.66, 0.93)	N/A
70–79	135 (6.23)	1.86 (1.56, 2.2)	− 3.14 (− 6.62, 0.69)	131 (6.05)	1.34 (1.12, 1.59)	2.08 (− 5.34, 12.22)
80+	46 (2.12)	1.41 (1.03, 1.88)	N/A	65 (3.00)	1.2 (0.93, 1.53)	− 6.36 (− 16.49, 3.46)

NHB						
Age groups	Men			Women		
	Case (%)	ASIR (95% CI)	AAPC (95% CI)	Case (%)	ASIR (95% CI)	AAPC (95% CI)
20+	833 (63.73)	0.76 (0.7, 0.81)	-4.3 (− 7.06, − 2.12)	474 (36.27)	0.38 (0.34, 0.41)	− 2.32 (− 5.4, 0.39)
20–29	137 (10.48)	0.52 (0.43, 0.61)	− 7.13(− 10.74, − 3.54)	66 (5.05)	0.24 (0.19, 0.31)	N/A
30–39	180 (13.77)	0.78 (0.67, 0.9)	− 2.23 (− 5.03, 0.41)	96 (7.35)	0.37 (0.3, 0.46)	− 2.4 (− 7.06, 2.47)
40–49	217 (16.60)	0.95 (0.83, 1.09)	− 4.23 (− 8.07, − 0.97)	97 (7.42)	0.38 (0.3, 0.46)	− 1.08 (− 4.96, 2.88)
50–59	173 (13.24)	0.91 (0.78, 1.06)	− 1.47 (− 5.17, 2.39)	92 (7.04)	0.42 (0.34, 0.51)	− 1.29 (− 4.87, 2.68)
60–69	81 (6.20)	0.71 (0.56, 0.88)	− 4.79 (− 8.76, − 0.79)	59 (4.51)	0.41 (0.31, 0.52)	− 6.24 (− 12.73, 0.12)
70–79	32 (2.45)	0.59 (0.4, 0.83)	N/A	49 (3.75)	0.59 (0.44, 0.78)	N/A
80+	13 (0.99)	0.55 (0.29, 0.94)	N/A	15 (1.15)	0.31 (0.17, 0.52)	N/A

NHW						
Age groups	Men			Women		
	Case (%)	ASIR (95% CI)	AAPC (95% CI)	Case (%)	ASIR (95% CI)	AAPC (95% CI)
20+	4452 (61.71)	0.71 (0.69, 0.73)	− 4.2 (− 4.82, − 3.71)	2762 (38.29)	0.39 (0.38, 0.41)	− 3.57 (− 4.28, − 2.96)
20–29	645 (8.94)	0.61 (0.57, 0.66)	− 2.49 (− 4.13, − 0.97)	315 (4.37)	0.31 (0.28, 0.34)	− 2.21 (− 4.61, 0.01)
30–39	589 (8.16)	0.55 (0.51, 0.6)	− 4.07 (− 6.95, − 2.1)	298 (4.13)	0.28 (0.25, 0.32)	− 0.66 (− 5.76, 2.44)
40–49	752 (10.42)	0.63 (0.59, 0.68)	− 7.61(− 10.44, − 4.49)	322 (4.46)	0.27 (0.24, 0.3)	− 2.6 (− 5.48, − 0.15)
50–59	815 (11.30)	0.68 (0.63, 0.73)	− 2.93 (− 3.93, − 2.01)	376 (5.21)	0.3 (0.27, 0.34)	− 3.58 (− 5.12, − 2.18)
60–69	730 (10.12)	0.85 (0.79, 0.91)	− 4.89 (− 6.58, − 3.3)	487 (6.75)	0.53 (0.48, 0.58)	− 4.99 (− 7.06, − 3)
70–79	599 (8.30)	1.16 (1.07, 1.26)	− 3.09 (− 5.42, − 1.19)	603 (8.36)	0.96 (0.88, 1.04)	− 3.46 (− 5.53, − 1.62)
80+	322 (4.46)	1.15 (1.02, 1.28)	− 5.28 (− 9.32, − 1.87)	361 (5.00)	0.76 (0.68, 0.84)	− 4.52 (− 7.31, − 2.26)

NHW, non-Hispanic White; NHB, non-Hispanic Black; ASIR, age-standardized incidence rate; CI, confidence interval; AAPC, average annual percent change; N/A, not available.

Men experienced a slight decrease in incidence rates between the age groups of 20–24 and 25–29, followed by a steady increase reaching a peak at 80–84. Women saw minimal fluctuations in the incidence rate between the ages of 20 and 44. From this point onward, the incidence rate progressively increased, reaching its peak at 75–79. Men consistently exhibited higher incidence rates compared to women (Fig. S8).

### Nodular sclerosis Hodgkin lymphoma (NSHL)

Over 2000–2019, 35,534 cases of NSHL in all age groups in the US were reported. The majority of the cases were men (50.23%), NHWs (70.58%), and between 20 and 29 years old (67.47%). The ASIR per 100,000 population was 1.72 (1.70, 1.75) for men and 1.65 (1.63, 1.67) for women. Both sexes experienced a significant decline in ASIRs over 2000–2019 (AAPC: − 2.48% [− 3.12, − 1.80] for men and AAPC: − 2.24% [− 2.64, − 1.82] for women). NHW men had the highest ASIR (2.16 [2.13, 2.20]). Hispanic men exhibited the greatest decline in ASIRs between 2000 and 2019 (AAPC: − 2.89% [− 4.42, − 0.95]) (Table 5, Figs. S9–S11). The details of incidence trends for males and females are provided in Appendix 1.

**Table 5:** Counts and age-standardized rate of nodular sclerosis Hodgkin lymphoma incidence per 100,000 and average annual percent change from 2000 to 2019 in the United States, by age, sex, and race.

All race/ethnicities						
Age group (years)	Men			Women		
	Case (%)	ASIR (95% CI)	AAPC (95%CI)	Case (%)	ASIR (95% CI)	AAPC (95%CI)
20+	17,850 (50.23)	1.72 (1.7, 1.75)	− 2.48 (− 3.12, − 1.8)	17,684 (49.77)	1.65 (1.63, 1.67)	-2.24 (− 2.64, − 1.82)
20–29	5436 (15.30)	2.55 (2.48, 2.62)	− 2 (− 2.8, − 1.21)	6546 (18.42)	3.2 (3.12, 3.27)	− 1.9 (− 2.42, − 1.4)
30–39	4193 (11.80)	2.02 (1.96, 2.09)	− 2.94 (− 3.53, − 2.41)	4463 (12.56)	2.16 (2.09, 2.22)	− 2.43 (− 2.89, − 2.01)
40–49	2957 (8.32)	1.46 (1.4, 1.51)	− 3.07 (− 4.06, − 2.21)	2467 (6.94)	1.2 (1.15, 1.25)	− 2.27 (− 3.21, − 1.43)
50–59	2109 (5.94)	1.16 (1.11, 1.21)	− 2.44 (− 3.14, − 1.78)	1498 (4.22)	0.79 (0.75, 0.83)	− 2.75 (− 3.6, − 1.95)
60–69	1504 (4.23)	1.23 (1.17, 1.29)	− 2.21 (− 3.92, − 0.75)	1148 (3.23)	0.84 (0.79, 0.89)	− 3.35 (-4.68, − 2.01)
70–79	1127 (3.17)	1.63 (1.54, 1.73)	− 1.36 (− 3.33, 0.81)	955 (2.69)	1.09 (1.02, 1.16)	− 3.2 (− 4.7, − 1.96)
80+	524 (1.47)	1.45 (1.33, 1.58)	− 2.48 (− 4.15, − 0.77)	607 (1.71)	0.98 (0.91, 1.06)	− 3.15 (− 4.69, − 1.76)

Hispanic						
Age groups	Men			Women		
	Case (%)	ASIR (95% CI)	AAPC (95%CI)	Case (%)	ASIR (95% CI)	AAPC (95%CI)
20+	2548 (48.68)	1.25 (1.19, 1.3)	− 2.89 (− 4.42, − 0.95)	2686 (51.32)	1.17 (1.12, 1.22)	− 2.64 (− 3.56, − 1.71)
20–29	818 (15.63)	1.32 (1.23, 1.42)	0.03 (− 2.89, 3.63)	1150 (21.97)	2.06 (1.95, 2.19)	− 1.6 (− 2.7, − 0.48)
30–39	591 (11.29)	1.06 (0.98, 1.15)	− 1.35 (− 3.18, 0.54)	662 (12.65)	1.26 (1.17, 1.36)	− 1.48 (− 4.02, 1.67)
40–49	418 (7.99)	0.95 (0.86, 1.05)	− 2.53 (− 5.09, 0.15)	330 (6.30)	0.77 (0.69, 0.85)	− 1.73 (− 4.53, 1.28)
50–59	287 (5.48)	0.99 (0.87, 1.11)	− 2.45 (− 4.04, − 0.7)	182 (3.48)	0.6 (0.52, 0.69)	− 4.09 (− 7.2, − 0.75)
60–69	197 (3.76)	1.28 (1.1, 1.47)	− 5.82 (− 8.11, − 3.49)	168 (3.21)	0.94 (0.8, 1.09)	− 5.57 (− 9.48, − 1.57)
70–79	169 (3.23)	2.32 (1.98, 2.7)	− 3.68 (− 5.67, − 1.57)	118 (2.25)	1.22 (1.01, 1.46)	− 4.62 (− 8.06, − 1.17)
80+	68 (1.30)	2.06 (1.6, 2.61)	N/A	76 (1.45)	1.41 (1.11, 1.76)	0.14 (− 4.51, 6.73)

NHB						
Age groups	Men			Women		
	Case (%)	ASIR (95% CI)	AAPC (95%CI)	Case (%)	ASIR (95% CI)	AAPC (95%CI)
20+	1682 (48.50)	1.46 (1.38, 1.53)	− 1.68 (− 3.78, 0.25)	1786 (51.50)	1.36 (1.29, 1.42)	− 1.89 (− 2.81, − 0.98)
20–29	553 (15.95)	2.09 (1.92, 2.27)	− 1.82 (− 4.13, 0.54)	664 (19.15)	2.44 (2.26, 2.63)	− 2.88 (− 5.33, − 0.83)
30–39	421 (12.14)	1.8 (1.63, 1.98)	− 3.11 (− 5.45, − 1)	499 (14.39)	1.92 (1.76, 2.1)	0.55 (− 3.37, 3.29)
40–49	328 (9.46)	1.45 (1.29, 1.61)	− 1.08 (− 3.56, 1.28)	267 (7.70)	1.05 (0.93, 1.18)	− 1 (− 3.19, 1.13)
50–59	209 (6.03)	1.11 (0.96, 1.27)	− 1.58 (− 3.78, 0.76)	176 (5.07)	0.8 (0.69, 0.93)	− 1.9 (− 4.56, 0.89)
60–69	102 (2.94)	0.89 (0.72, 1.08)	− 6.36 (− 17.05, 1.59)	113 (3.26)	0.79 (0.65, 0.94)	− 2.89 (− 5.76, 0.2)
70–79	52 (1.50)	0.96 (0.72, 1.26)	N/A	46 (1.33)	0.57 (0.41, 0.75)	N/A
80+	17 (0.49)	0.73 (0.43, 1.18)	N/A	21 (0.61)	0.43 (0.27, 0.66)	N/A

NHW						
Age groups	Men			Women		
	Case (%)	ASIR (95% CI)	AAPC (95%CI)	Case (%)	ASIR (95% CI)	AAPC (95%CI)
20+	12751 (50.84)	2.16 (2.13, 2.2)	− 2.38 (− 3.12, − 1.75)	12328 (49.16)	2.1 (2.06, 2.14)	− 1.99 (− 2.86, − 1.4)
20–29	3724 (14.85)	3.54 (3.43, 3.66)	− 2.35 (− 2.98, − 1.76)	4303 (17.16)	4.22 (4.1, 4.35)	− 1.76 (− 3.1, − 0.79)
30–39	2952 (11.77)	2.73 (2.64, 2.84)	− 2.8 (− 3.39, − 2.29)	3075 (12.26)	2.89 (2.79, 3)	− 2.39 (− 3.05, − 1.82)
40–49	2095 (8.35)	1.77 (1.7, 1.85)	− 2.63 (− 3.7, − 1.82)	1777 (7.09)	1.51 (1.44, 1.59)	− 1.67 (− 2.71, − 0.78)
50–59	1538 (6.13)	1.29 (1.23, 1.36)	− 2.32 (− 3.18, − 1.51)	1088 (4.34)	0.9 (0.85, 0.95)	− 2.25 (− 3.25, − 1.32)
60–69	1144 (4.56)	1.33 (1.25, 1.41)	− 1.69 (− 4.68, 0.41)	831 (3.31)	0.89 (0.83, 0.96)	− 2.93 (− 4.32, − 1.52)
70–79	876 (3.49)	1.71 (1.59, 1.82)	− 0.7 (− 2.85, 1.84)	760 (3.03)	1.2 (1.12, 1.29)	− 3.17 (− 4.76, − 1.71)
80+	422 (1.68)	1.5 (1.36, 1.65)	− 1.92 (− 3.84, 0.01)	494 (1.97)	1.03 (0.94, 1.13)	− 3.59 (− 5.34, − 2.12)

NHW, non-Hispanic White; NHB, non-Hispanic Black; ASIR, age-standardized incidence rate; CI, confidence interval; AAPC, average annual percent change; N/A, not available.

Both sexes showed declines in incident cases with advancing age. Moreover, both sexes revealed decreases in incidence rates, reaching the minimum rate at 55–59 age group. Subsequently, they increased until 75–79 age group for both sexes. Men exhibited higher incidence rates compared to women in all age groups except for the ages of 20–39 years (Fig. S12).

### Classical Hodgkin lymphoma not otherwise specified (CHL-NOS)

From 2000 to 2019, there were 20,139 CHL-NOS cases in all age groups in the US. The majority of cases were men (57.91%), NHWs (63.46%), and aged 20–29 years (17.00%). The ASIR per 100,000 population was 1.16 (1.13, 1.18) for men and 0.76 (0.74, 0.78) for women. Both sexes experienced increases in ASIRs between 2000 and 2019 (AAPC: 2.60% [2.07, 3.22] for males and AAPC: 3.89% [3.29, 4.70] for females) (Table 6, Figs. S13–S15). The details of incidence trends for males and females are provided in Appendix 1.

**Table 6:** Counts and age-standardized rate of classical Hodgkin lymphoma not otherwise specified incidence per 100,000 and average annual percent change from 2000 to 2019 in the United States, by age, sex, and race.

All race/ethnicities						
Age group (years)	Men			Women		
	Case (%)	ASIR (95% CI)	AAPC (95%CI)	Case (%)	ASIR (95% CI)	AAPC (95%CI)
20+	11,663 (57.91)	1.16 (1.13, 1.18)	2.6 (2.07, 3.22)	8476 (42.09)	0.76 (0.74, 0.78)	3.89 (3.29, 4.7)
20–29	1841 (9.14)	0.86 (0.82, 0.9)	4.44 (3.61, 5.47)	1582 (7.86)	0.77 (0.73, 0.81)	4.46 (3.41, 5.76)
30–39	1828 (9.08)	0.89 (0.85, 0.93)	2.74 (1.61, 4.01)	1329 (6.60)	0.64 (0.61, 0.68)	4.98 (4.26, 5.87)
40–49	1854 (9.21)	0.91 (0.87, 0.95)	2.13 (1.3, 3.06)	1106 (5.49)	0.53 (0.5, 0.56)	3.45 (2.31, 4.76)
50–59	2009 (9.98)	1.1 (1.05, 1.15)	2.69 (1.92, 3.6)	1099 (5.46)	0.57 (0.54, 0.61)	2.67 (1.41, 4.18)
60–69	1768 (8.78)	1.45 (1.39, 1.52)	1.88 (0.7, 3.36)	1230 (6.11)	0.9 (0.85, 0.96)	1.93 (0.64, 3.5)
70–79	1502 (7.46)	2.18 (2.07, 2.29)	1.95 (0.99, 3.04)	1222 (6.07)	1.4 (1.32, 1.48)	1.67 (− 0.21, 3.86)
80+	861 (4.28)	2.4 (2.24, 2.56)	6.42 (0.83, 13.02)	908 (4.51)	1.46 (1.37, 1.56)	1.72 (0.3, 3.33)

Hispanic						
Age groups	Men			Women		
	Case (%)	ASIR (95% CI)	AAPC (95%CI)	Case (%)	ASIR (95% CI)	AAPC (95%CI)
20+	2309 (60.02)	1.37 (1.31, 1.44)	3.01 (1.92, 4.43)	1538 (39.98)	0.82 (0.78, 0.87)	3.19 (2.2, 4.52)
20–29	405 (10.53)	0.65 (0.59, 0.72)	5.06 (2.99, 7.72)	347 (9.02)	0.62 (0.56, 0.69)	6.83 (4.91, 9.67)
30–39	408 (10.61)	0.74 (0.67, 0.81)	2.03 (0.17, 4.25)	236 (6.13)	0.45 (0.39, 0.51)	5.44 (3.47, 8.17)
40–49	387 (10.06)	0.88 (0.8, 0.98)	0.47 (− 1.72, 3.07)	198 (5.15)	0.46 (0.4, 0.53)	3.82 (1.92, 6.41)
50–59	386 (10.03)	1.33 (1.2, 1.47)	1.96 (− 0.6, 5.49)	209 (5.43)	0.69 (0.6, 0.79)	1.08 (− 1.07, 3.89)
60–69	327 (8.50)	2.14 (1.91, 2.39)	2.69 (0.99, 4.97)	217 (5.64)	1.22 (1.07, 1.4)	2.55 (0.12, 6.01)
70–79	280 (7.28)	3.8 (3.37, 4.28)	3.38 (0.85, 6.98)	220 (5.72)	2.24 (1.96, 2.56)	0.9 (− 3.33, 11.57)
80+	116 (3.02)	3.54 (2.92, 4.25)	**N/A**	111 (2.89)	2.05 (1.69, 2.47)	0.32 (− 3.64, 6.18)

NHB						
Age groups	Men			Women		
	Case (%)	ASIR (95% CI)	AAPC (95%CI)	Case (%)	ASIR (95% CI)	AAPC (95%CI)
20+	1488 (58.86)	1.37 (1.3, 1.45)	2.42 (1.36, 3.68)	1040 (41.14)	0.81 (0.76, 0.86)	3.4 (2.12, 4.94)
20–29	270 (10.68)	1.02 (0.91, 1.15)	3.58 (0.29, 8.21)	210 (8.31)	0.77 (0.67, 0.88)	3.87 (1.43, 7.06)
30–39	320 (12.66)	1.39 (1.24, 1.56)	2.62 (0.28, 5.4)	207 (8.19)	0.8 (0.7, 0.92)	4.69 (1.31, 9.09)
40–49	337 (13.33)	1.48 (1.33, 1.65)	1.36 (− 0.52, 3.38)	195 (7.71)	0.76 (0.66, 0.87)	2.03 (− 1, 5.31)
50–59	297 (11.75)	1.57 (1.4, 1.76)	1.79 (− 0.35, 4.38)	173 (6.84)	0.78 (0.67, 0.91)	2.77 (− 0.48, 7.09)
60–69	152 (6.01)	1.36 (1.15, 1.59)	1.93 (− 1.48, 6.7)	130 (5.14)	0.89 (0.74, 1.06)	2.45 (− 1.11, 7.73)
70–79	79 (3.13)	1.45 (1.14, 1.81)	3.06 (0.45, 6.85)	75 (2.97)	0.9 (0.71, 1.13)	5.76 (3.16, 9.91)
80+	33 (1.31)	1.43 (0.98, 2.01)	N/A	50 (1.98)	1.04 (0.77, 1.38)	0.22 (− 4.61, 6.06)

NHW						
Age groups	Men			Women		
	Case (%)	ASIR (95% CI)	AAPC (95%CI)	Case (%)	ASIR (95% CI)	AAPC (95%CI)
20+	7295 (57.08)	1.16 (1.13, 1.19)	2.65 (2.09, 3.27)	5486 (42.92)	0.81 (0.78, 0.83)	3.81 (2.86, 5.09)
20–29	1020 (7.98)	0.97 (0.91, 1.03)	4.37 (2.81, 6.3)	914 (7.15)	0.9 (0.84, 0.96)	3.81 (2.61, 5.21)
30–39	1005 (7.86)	0.93 (0.88, 0.99)	3.1 (1.17, 5.3)	791 (6.19)	0.75 (0.7, 0.8)	4.94 (3.63, 6.52)
40–49	1064 (8.32)	0.89 (0.84, 0.94)	2.86 (1.84, 3.9)	660 (5.16)	0.55 (0.51, 0.59)	3.62 (1.73, 5.66)
50–59	1228 (9.61)	1.03 (0.97, 1.08)	2.8 (2.08, 3.6)	685 (5.36)	0.56 (0.52, 0.6)	2.47 (0.64, 4.54)
60–69	1211 (9.48)	1.41 (1.33, 1.49)	1.4 (0.16, 2.92)	840 (6.57)	0.91 (0.85, 0.97)	1.33 (− 0.4, 3.38)
70–79	1086 (8.50)	2.12 (1.99, 2.25)	1.87 (− 0.2, 4.29)	876 (6.85)	1.39 (1.3, 1.48)	1.29 (− 0.6, 3.39)
80+	681 (5.33)	2.43 (2.25, 2.62)	5.56 (0.46, 11.31)	720 (5.63)	1.49 (1.39, 1.61)	1.89 (− 0.03, 3.99)

NHW, non-Hispanic White; NHB, non-Hispanic Black; ASIR, age-standardized incidence rate; CI, confidence interval, AAPC, average annual percent change; N/A, not available.

The incident cases of CHL-NOS in men remained relatively stable from ages 20 to 69, after which it began to decline, reaching its lowest point over 85. In contrast, there was a fluctuation for women, decreasing from 20–24 to 50–54, followed by an increase to 60–64 and stabilization until 70–74, then a subsequent decline. Men had minimal fluctuation from 20–24 to 45–49 in terms of incidence rates. Then, the incidence rate of men experienced an increase until 80–84 years, followed by a steep decline. For women, there was a minimal decline from 20–24, reaching the minimum at 50–54, and from this point onward, there was a steady increase until 80–84. In all age groups, men exhibited higher incidence rates than women (Fig. S16).

### Nodular lymphocyte prominent Hodgkin lymphoma (NLPHL)

Over 2000–2019, there were 4,063 cases of NLPHL in the US across all age groups. The majority of cases were observed in men (64.66%), NHWs (58.85%), and cases between 40 and 49 years (19.71%). The ASIR per 100,000 population was 0.25 (0.24, 0.26) for men and 0.13 (0.12, 0.14) for women. Both sexes experienced significant increases in ASIRs over 2000–2019 (AAPC: 4.95% [3.77, 6.45] for men and AAPC: 6.33% [5.10, 7.94] for women) (Table 7, Figs. S17–S19). The details of incidence trends for males and females are provided in Appendix 1.

**Table 7:** Counts and age-standardized rate of nodular lymphocyte prominent Hodgkin lymphoma incidence per 100,000 and average annual percent change from 2000 to 2019 in the United States, by age, sex, and race.

All race/ethnicities						
Age group (years)	Men			Women		
	Case (%)	Delayed ASIR (95% CI)	AAPC (95%CI)	Case (%)	Delayed ASIR (95% CI)	AAPC (95%CI)
20+	2627 (64.66)	0.25 (0.24, 0.26)	4.95 (3.77, 6.45)	1436 (35.34)	0.13 (0.12, 0.14)	6.33 (5.1, 7.94)
20–29	466 (11.47)	0.22 (0.2, 0.24)	3.45 (1.57, 5.73)	189 (4.65)	0.09 (0.08, 0.11)	N/A
30–39	525 (12.92)	0.26 (0.23, 0.28)	4.39 (1.93, 7.43)	189 (4.65)	0.09 (0.08, 0.11)	2.59 (− 1.28, 7.51)
40–49	534 (13.14)	0.26 (0.24, 0.28)	6.75 (5, 9)	267 (6.57)	0.13 (0.11, 0.14)	8.98 (7.38, 11.27)
50–59	520 (12.80)	0.28 (0.26, 0.31)	4.94 (3.14, 8.46)	305 (7.51)	0.16 (0.14, 0.18)	6.6 (4.72, 9.37)
60–69	366 (9.01)	0.3 (0.27, 0.33)	3.52 (1.75, 6.03)	275 (6.77)	0.2 (0.18, 0.23)	5.21 (1.61, 11.44)
70–79	169 (4.16)	0.24 (0.21, 0.28)	3.4 (0.8, 7.12)	169 (4.16)	0.19 (0.16, 0.22)	5.83 (3.77, 8.74)
80+	47 (1.16)	0.13 (0.09, 0.17)	N/A	42 (1.03)	0.07 (0.05, 0.09)	N/A

Hispanic						
Age groups	Men			Women		
	Case (%)	Delayed ASIR (95% CI)	AAPC (95%CI)	Case (%)	Delayed ASIR (95% CI)	AAPC (95%CI)
20+	321 (64.46)	0.15 (0.14, 0.17)	3.57 (1.48, 6.56)	177 (35.54)	0.09 (0.08, 0.11)	4.2 (0.71, 9.74)
20–29	84 (16.87)	0.14 (0.11, 0.17)	N/A	23 (4.62)	0.04 (0.03, 0.06)	N/A
30–39	74 (14.86)	0.14 (0.11, 0.17)	3.4 (− 1.75, 10.64)	25 (5.02)	0.05 (0.03, 0.07)	N/A
40–49	61 (12.25)	0.14 (0.11, 0.18)	N/A	44 (8.84)	0.1 (0.07, 0.14)	N/A
50–59	58 (11.65)	0.2 (0.15, 0.26)	N/A	34 (6.83)	0.11 (0.08, 0.16)	N/A
60–69	26 (5.22)	0.17 (0.11, 0.25)	N/A	30 (6.02)	0.17 (0.11, 0.24)	N/A
70–79	13 (2.61)	0.18 (0.09, 0.31)	N/A	16 (3.21)	0.16 (0.09, 0.27)	N/A
80+	5 (1.00)	0.15 (0.05, 0.35)	N/A	5 (1.00)	0.09 (0.03, 0.21)	N/A

NHB						
Age groups	Men			Women		
	Case (%)	Delayed ASIR (95% CI)	AAPC (95%CI)	Case (%)	Delayed ASIR (95% CI)	AAPC (95%CI)
20+	498 (50.51)	0.44 (0.41, 0.49)	5.98 (3.93, 8.93)	488 (49.49)	0.38 (0.35, 0.42)	6.14 (3.96, 9.31)
20–29	82 (8.32)	0.31 (0.25, 0.38)	6.71 (3.33, 12.06)	64 (6.49)	0.24 (0.18, 0.3)	N/A
30–39	106 (10.75)	0.45 (0.37, 0.55)	5.41 (0.85, 11.66)	97 (9.84)	0.37 (0.3, 0.46)	N/A
40–49	130 (13.18)	0.57 (0.47, 0.67)	N/A	119 (12.07)	0.46 (0.38, 0.55)	9.35 (6.99, 13.01)
50–59	103 (10.45)	0.55 (0.45, 0.66)	N/A	103 (10.45)	0.47 (0.38, 0.57)	N/A
60–69	56 (5.68)	0.5 (0.37, 0.65)	N/A	71 (7.20)	0.49 (0.39, 0.62)	N/A
70–79	18 (1.83)	0.33 (0.19, 0.52)	N/A	30 (3.04)	0.36 (0.25, 0.52)	N/A
80+	3 (0.30)	0.12 (0.03, 0.37)	N/A	4 (0.41)	0.09 (0.02, 0.22)	N/A

NHW						
Age groups	Men			Women		
	Case (%)	Delayed ASIR (95% CI)	AAPC (95%CI)	Case (%)	Delayed ASIR (95% CI)	AAPC (95%CI)
20+	1684 (70.43)	0.27 (0.26, 0.28)	4.87 (4.02, 5.84)	707 (29.57)	0.1 (0.09, 0.11)	6.25 (4.73, 8.28)
20–29	273 (11.42)	0.26 (0.23, 0.29)	2.01 (− 0.26, 4.63)	92 (3.85)	0.09 (0.07, 0.11)	N/A
30–39	324 (13.55)	0.3 (0.27, 0.34)	4.93 (1.65, 8.98)	60 (2.51)	0.06 (0.04, 0.07)	N/A
40–49	318 (13.30)	0.26 (0.24, 0.29)	6.57 (4.85, 8.63)	95 (3.97)	0.08 (0.06, 0.1)	N/A
50–59	333 (13.93)	0.28 (0.25, 0.31)	4.85 (3.06, 7.96)	148 (6.19)	0.12 (0.1, 0.14)	6.74 (3.38, 11.79)
60–69	269 (11.25)	0.31 (0.27, 0.35)	3.32 (1.18, 6.27)	160 (6.69)	0.17 (0.15, 0.2)	N/A
70–79	131 (5.48)	0.25 (0.21, 0.3)	3.53 (0.58, 7.66)	120 (5.02)	0.19 (0.16, 0.23)	5.45 (2.44, 9.9)
80+	36 (1.51)	0.13 (0.09, 0.18)	N/A	32 (1.34)	0.07 (0.05, 0.1)	N/A

NHW, non-Hispanic White; NHB, non-Hispanic Black; ASIR, age-standardized incidence rate; CI, confidence interval; AAPC, average annual percent change; N/A, not available.

There was an overall increase in incidence rates in both sexes, peaking at 55–59 for men and 65–69 for women. Subsequently, both sexes experienced a decline until ages over 85 years. Also, men exhibited higher incidence rates compared to women (Fig. S20).

## Discussion

Our study primarily delineated the incidence trend of HL as a relatively rare hematological malignancy in the US. As a SEER program-based study that exclusively assessed the US population, we mainly focused on different epidemiological features of the various HL histopathological subtypes in 20 years and especially presented updates on trend changes from the early 2000s, which was also partly discussed by Glaser et al.^33^ and Evens et al.^34^, up to 2019, when the severe acute respiratory syndrome coronavirus 2 (SARS-CoV-2) started to spread globally and drastically influenced cancer diagnostic and management processes. COVID-19 pandemic caused a significant decline in the ASIRs of the HL cases among all races and age groups. Our data demonstrated that the overall incidence of HL in both sexes decreased between 2000 and 2019 and had an approximately doubled pace in the last five years. However, the CHL-NOS subtype and NLPHL showed an increasing trend. Generally, in all HL subtypes, men comprised the higher proportion of the population and had an overall higher delayed ASIR, with the highest incidence rate among the ages 70 years and older. However, in the age group of 20–29, women represented slightly more cases and had higher ASIRs. While assessing the age composition, the 20–29 individuals were considered the most incident age group in the HL subtype population, except for the NLPHL and LR/MC/LD, in which the cases in their 40s and 50s, respectively, are the dominant age cluster. The present study demonstrated that NHW cases were the most frequent racial subgroup in the HL population in the US, although varieties were seen among distinct histological entities. From the histopathological point of view, among the NSHL subtype, NHWs had the highest age-adjusted incidence rates. In contrast, Hispanics showed dominancy in the MC subtype, which was also pointed out in the study performed by Evens et al.^34^. Moreover, NHB was the leading racial subgroup in NLPHL and CHL-NOS, as depicted in the survey conducted by Shenoy et al.^35^. Despite the overall decline in the HL incidence in all racial subgroups, NHB women depicted a positive AAPC.

Our analysis revealed that our data almost aligns with the previous studies conducted in the US. An early SEER-based paper that evaluated the population data from 1992 to 2011^33^ showed that the CHL incidence trend, as the most common HL subgroup comprising 95% of its cases, was nearly stable until 2007 and subsequently started to diminish. In our study, the incidence of CHL could be subclassified based on age categories. The age group 30–69 had a diminishing incidence without any joinpoint (JP) while illustrating the joinpoint regression analysis. The age range of 20–29 and 70–79 revealed a steady decline from its JP around 2004–2006. Despite the mentioned study in which the CHL incidence of the age group 80+ was relatively increased from 2008 to 2011^33^, our analysis indicated that their incidence patterns declined from 2004 to 2019. In a detailed evaluation of our data regarding NSHL, the most common subtype of classic HL which includes almost half of all CHL instances, we obtained that men showed a declining incidence pattern without a JP. Still, women showed an incidence rise from 2011 to 2019, probably explained in the following text, subsequent to a decreasing trend discussed by Glaser et al.^33^. While analyzing the NSHL statistics of the racial subgroups, both studies suggest a more rapid decrease that follows a slower decline with a JP around 2009–2011 in Hispanics and 2015 in NHWs. In our study, when analyzing the LR/MC/LD population concerning age subgroups, an overall decrease without JP was observed, while an initial rise in age groups 20–29, 40–49, and 70–79 was seen. Glaser et al. substantially agreed with our results, and Huang et al.^1^, relying on a worldwide evaluation, pointed out that the MC incidence decline was accelerated from 2009–2010.

In our study, NHWs’ highest ASIR was around 55–65 in males and 20–29 in females, while it was 20–29 in both sexes in Evens et al.^34^, which evaluated the HL trend in the US population data in 1992–2007. However, our study presented the age group of 30–39 in men and the age group of 20–29 in women as the NHB’s highest ASIRs, whereas it was 40–49 in males and 30–39 in females of the Blacks in the previous study. This reveals an approximately 10-year shift in the highest incidence age of the mentioned racial subgroup. Both sexes of Hispanics showed the highest ASIR of the 70–79 age group in both studies. Although this increasing trend in NHBs could be related to population aging, there is no convincing reason for this growing ASIRs in NHW males. Additionally, Evens et al.^34^ demonstrated that whites had the highest incidence in patients aged under 65 years, and Hispanics had the highest incidence in patients aged more than 65 years, which is also confirmed by our findings. It has been discussed in either of previous studies conducted on the US population that the declining trend of the CHL, accompanied by relevant and predictable changes in its major NSHL subtype and the presence of normal variations in diverse age and sex trends, is proof of a realistic change. Still, the MC rate decrement and similar reciprocal two–three-fold increments in NOS incidence with an age- and sex-disproportional pattern bring underlying false incidence changes in the mind. The identical incidence trend pattern of MC between 1992 and 1996 and the pattern of NOS between 2007 and 2011, revealed by Glaser et al., potentiates the proposed theory. Regarding the recent definition of NLP as a new disease entity, an increased incidence rate of NLP during the timespan among different subgroups could be reasonable and is also mentioned in both discussed articles. However, an actual incidence change cannot be overseen.

In accordance with our study, global patterns in HL incidence reported by Shen et al.^36^ revealed an ASIR decrement from 1999 to 2019. Global 2020 investigation has revealed that Australia, New Zealand, and the US are among the countries with the highest ASIR, after South, North, and West European countries. In male populations, a higher risk of HL is related with higher Gross Domestic Product (GDP) per capita, smoking (odds ratio = 1.1), obesity (a 10% higher risk for a body mass index increase of 5 kg/m^2^), and hypertension. In the women population, smoking and obesity are related to a higher risk of HL. Although alcohol consumption may increase the risk of HL in women aged under 50, smoking is more considerable in women aged above 50 years^1^. According to a study on national cancer registry data in Iran, the crude incidence rates of cancer were 10.41 and 7.28 among males and females, respectively, in 2016, reflecting increases of 3.4% and 4.3% in males and females, respectively, from 2000 to 2016^37^. Consequently, the incidence rates in Iran was higher than those in the US. However, it should be noted that there are differences in the reported measures (crude rate vs. age-standardized rate). Additionally, the study based on cancer registry in Iran did not differentiate between various types of hematological cancers, combining all cases of HL, non-HL, leukemia, and multiple myeloma together^37^. Moreover, another study in Tehran, the capital of Iran, showed that the ASIRs of hematological cancers among men and women were 99.5 and 16.2 per 100,000 over 1999–2014, respectively^38^. The rising incidence and greater ASIRs might be due to population growth or ageing, smoking, and air pollution^39,40^. Our study declares that NSHL had a decreasing rate from about 2005 to 2006, which could be related to approximately 1.8% overall GDP decline in the US from 2006 to 2016. Although GDP is not the only reason, it can be one of the contributing factors along with the reduction of smoking prevalence^41^. The lower incidence of HL in countries with lower GDP/income is related to a lack of powerful equipment and resultant undiagnosed or misclassified cases^36^. In a general review of this study^1^, the increasing trend of HL incidence in younger people and women is associated with the increase in obesity and metabolic diseases. The global weight gain and obesity in women has increased from 30% in 1980 to 38% in 2013 and 41.9% in 2018, which also had a higher pace than men in the recent three decades^42^. The global burden of the disease study conducted from the data collected between 1980 and 2015 confirmed that a higher sociodemographic index correlates with an increased prevalence of obesity^43^. Additionally, the study delineated that women had higher prevalence of obesity in all age groups and the most prominent increase in the obesity rate belongs to early adulthood^43^. Otherwise, between 1985 and 2014, central obesity had a more significant escalation in younger ages (15–40 age) and Asian women represented a higher HL incidence in global investigations^44^. A study comparing HL between native and US-born Asians showed that US born-Asian had more than twice the prevalence of HL, which also makes the role of environmental factors more evident^45^. Otherwise, a study revealed that a better quality of life in early childhood, possibly evident in US-born children, positively correlates with metabolic disorders and consequently would increase the possibility of HL at younger ages^46^. Among the 21 regions of our planet assessed for further clarifying the epidemiology of the lymphoma by Shen et al.^36^, Caribbean populations had the highest ASIRs for HL which was postulated to be associated with prominent EBV and HIV infection prevalence there.

In respect of other environmental factors affecting the histopathological trend of NSHL, the recent decrease in NSHL in our study could mainly be attributed to a lowered rate of early social isolation^47,48^. Studies have reported associations between lowered risk of lymphoma and larger sibship size and/or later birth order for lymphoid^49^. Besides, pre-school attendance is a known protective condition for this subtype reduced rate^50^. None of these factors could explain the difference in sex incidence in NSHL patients. While assessing the causes of higher HL in females, as it was seen in our study, in which the overall CHL rate was higher in females in the 20–35 age range. Zhu et al. presented that multi-parity and younger age first gravidity reduced the HL risk. Subsequently, reluctance to have more children and pregnancy at an older age impose a higher risk of NSHL in younger nulliparous women. Also, Zhu et al.^51^ concluded that Aspirin consumption, smoking, and EBV infection could not reasonably discuss the sex incidence difference in NSHL patients. Evens et al.^34^ declared that in their 15-year study, the rise in HL incidence mainly occurred in the 15–45-year ages, and cases predominantly belonged to the NS subtype. It has been demonstrated that NS subgroup positively correlated to socioeconomic status improvement and higher quality of care of the population of the study, which directly correlates with increased risk of metabolic syndrome^34,52^.

Regarding NSHL, the incidence rate in the US did not show a distinguishable change similar to other studies assessing NSHL trends in distinct regions in the globe^53–55^, but noticeable increases were reported in Australia until 2006 and in Japan until 2008. For young adults, the rising incidence of NSHL has been reported previously. In Australia, the NSHL rate rose 5% from 1997 until 2006, and an overall decrease was mentioned. The Automated Childhood Cancer Information System association also reported an incidence rise of HL (mostly NSHL), approximately 1% and 3.5% in childhood and early adolescent between 15 and 19 years, respectively^56^. Our study concluded that the overall incidence of HL in the > 20-year population declined, which could be discussed by the study of Hjalgrim et al.^57^, reporting that HL incidence markedly diminished in patients over 40 years, but children and young adults showed a discernable rise in the HL rate between 1978 and 1997.

In the study by Glaser et al. in 2015^33^, MC and NOS had diminished and raised incidence rates, respectively, which aligned with previous studies in the US and Australia^58,59^. The MC rate lessened in distinct racial subgroups between 1988–1992 and 2000–2004, mainly due to diagnostic problems and misclassification of subtypes. This principally resulted from the substitution of fine needle aspiration biopsy and core needle biopsy with conventional excisional biopsy, from which a small amount of body tissue with poorer quality was obtained^60,61^. About 12% of the obtained samples, primarily diagnosed as NOS, were identified to have insufficient cells. Modified tissue gathering procedures affect the NSHL detection less prominently than MC due to the preserved subtype morphology, even with a needle biopsy. Reduced specificity in histological classification caused complicated and less successful trend analysis of distinct HL subtypes and assessment of environmental cofounders. Furthermore, lower specified classification of HL subtypes caused NOS to be the second most common CHL subclass following NSHL, diminishing the need for genetic profiling, transcriptional analysis, and overall healthcare diagnosis costs^62–64^. Although differentiating various HL subgroups did not exert a major effect on treatment adjustment, it would assist in predicting patient survival and choosing tailored treatment^64–66^. As stated by Ye et al.^54^ in the Canadian society, a reasonable mechanism for HL decline in our study, is reduced HIV prevalence. HIV prevalence decreased by 38% from 2010 to 2022 globally, which could be a potent factor in the HL decreasing trend, especially in the MC subtype. As Grulich et al.^67^ declared, HIV infection could raise the risk of HL by 11 times. In contrast to the APC of MC subtype in the US, which demonstrated a platue since mid-1990s and a fall following the early 2000s, with the highly active antiretroviral therapy treatment being launched and widely used^68^ and more adoption to a westernized life style^69^, the MC subtype ASIR in the Japanese population raised from 1993 to 2008 delineated by Chihara et al.^70^. This could be at least partially explained by the increasing HIV rate in the same era, despite the nearly constant or slightly increasing HIV rate in Japan from 2006^71^. Glaser et al.^33^ and McNally et al.^72^ reported that MC is associated with low socioeconomic status. Consequently, life welfare improvement lowered the rate of MC. An approximately 50% lowered smoking rate since 2005^41^, which was confirmed to be more significant in countries with higher income^73^, and a westernized lifestyle are also considered as reasons for the decreased MC rate in the US. Although Juntikka et al.^53^ affirmed that NS was more common in women at a younger age, it emphasized that MC was more common in men in all age subgroups and was mostly prevalent in older ages. This report in Finland discussed that a significant MC decrease in ASIR was seen in their 20-year survey. Younger ages had a more appreciable ASIR decline, but there was no considerable decrement in older adults. The effect of EBV on the MC trend and the relation between the anti-EBV antibodies and its associated cancers was investigated as a reason for this decline MC trend in the US^74,75^. Although no significant changes have been reported regarding LR, a considerable decrease in LD has been seen in Finland till 2015. NOS incidence has been constant during the study which strengthens the theory of HIV and EBV effect. Solans et al.^55^ additionally pointed out that HL incidence was higher in Southern Europe and lower in Eastern Europe and accused the EBV infection, HIV, socioeconomic factors, differences in diagnosis precision, and health system availability as the possible reasons.

In our study, similar to the study by Strobbe et al.^76^ in the Netherlands, an increased incidence of NLPHL had been reported. This study has confirmed previous studies that have pointed out the effect of pathology misclassification as 2.8% to 20%. NLPHL increase could be associated with several associated factors: (1) better and more widely recognition of this subtype as a new distinct disease entity in 1994, (2) further development of NLPHL diagnostic tools, immunohistochemistry technology and its availability leading to more accurate diagnosis of these cases, and (3) improvements in the recording process of hematologic malignancies in the hematopathologic laboratories since 2000. An article from Finland^53^ reported that NLPHL incidence has not made any remarkable changes. As suggested by our study, NLP was more common in men and was diagnosed at few younger ages in men. Although the main reason was unclear, one of the considerable reasons could be the wider distribution of a germline mutation in 11q22-23 site which initially detected in Finland.

Following the initial spread of SARS-CoV-2 and residing interpersonal restrictions, including social isolation, plenty of adverse effects harmed the healthcare system. Most healthcare costs were spent on diagnosis, medical treatment and rehabilitation of COVID-19 patients, and even most hospitals were specialized only for dealing with the pandemic^77^. Besides, patients were neglected due to the decline in the number of working medical staff either who affected with the disease or who were redirected from different centers to new viral disease centers exacerbates the condition. Not referring to general clinics or hospitals due to fear of getting COVID-19 and converting out-patient visits to telemedicine had significantly affected the patients with cancer or high probability to acquire one. Otherwise, low availability of imaging, difficulty of public transportation, and depressed emotional support had all negative effects on these patients^78^. These adverse effects were more evident at the pandemic's beginning because of the authority-organized lockdown and the health system's incapability of handling enormous patient load. In the first months after the initial disease spread, cancer screening was not performed thoroughly, and people did not pursue medical aid due to social anxiety and the stress of getting the disease. The decline in new cancer diagnoses was reported by 40–50% in most cancers compared to the previous year^79^. Also, most of the accomplished diagnoses were at an advanced stage, which is also confirmed about lung cancer in many studies^80^. Different studies have proved that delays in screening cause a considerable decrease in diagnosed cases and higher incidence in advanced stages of cancer reducing their survival rate^81,82^. All histological subgroups of HL in our study presented a lower incidence while comparing 2019 statistics with past years. This delay resulted in not starting treatment on time, increased mortality, and decreased survival. Also, in patients whose treatment has begun, the pandemic caused a delay in the regular continuation of the radiotherapy process. This irregular schedule, along with emotional injuries, was a disturbing factor for these patients. Investigations showed that approximately one out of three of lymphoma cases in the COVID-19 time were suffered from anxiety, depression, and post-traumatic stress disorder^83^. Also, studies have revealed that patients with cancers had a 60% higher risk of getting infected with COVID-19^84^. Chemotherapy drugs increased the risk of getting infected with COVID-19. COVID-19 caused more severe symptoms in these cases and higher mortality in patients with hematological malignancies^77,85^.

Our study had strengths and limitations which are necessary to be acknowledged. We used the large database of SEER for evaluating and clarifying the incidence patterns of histological subtypes of HL among adults in the US. Additionally, in this study we conducted JP regression analyses, parallelism and coincidence tests. Assessing pre- and post-COVID-19 effect on the incidence trend of HL was necessary and is counted on as a strength point of our study. On the other side, several limitations could be mentioned. Registered data are always considered to have several potential bias. Deficient central pathology reanalysis and lack of final diagnosis confirmation by a hematopathologist, and also refusing to resample and recode the previously diagnosed NOS subtypes all could contribute to increased risk of detection and classification bias. In this article, we merely focused on the incidence trend analysis and other studies are needed to further evaluate survival and region-based analysis of the data and evaluate the risk factor association of each subtype. Another distinguishable racial disparity of our study is that Asian/Pacific Americans were not considered as a distinct ethnicity which could potentially affect the way secular trends would be interpreted. However, they were included for reporting the overall numbers and rates. Reporting the results of our histopathological subgroup, it is worth mentioning that separate MC, LR, and LD subgroups are discussed in our article as one CHL subcategory which may interfere with analyses the each HL subtype separately.

## Conclusions

Overall HL incidence and NSHL subtype, as the largest histopathological subtype of HL, decreased in 20 years in the US among both sexes, however, the CHL-NOS and NLPHL subtypes showed an increasing trend. The age group of 20–29 years comprises the majority of cases, and from the racial aspect, NHWs were the dominant subcategory. The COVID-19 pandemic resulted in an overall decline in the incidence of HL in all age groups, races, and sexes.

## Supplementary Information

Supplementary Information 1.

Supplementary Information 2.

## Data Availability

The data presented in this study are available at https://seer.cancer.gov/data-software/.
