# Supplementary material for: Adult Hodgkin lymphoma incidence trends in the United States from 2000 to 2020
Source: Sci Rep. 2024 Sep 3;14:20500. doi: 10.1038/s41598-024-69975-3 (PMC11372180; doi:10.1038/s41598-024-69975-3)
Supplement: Supplementary file 1 — Supplementary Information 1. [file 41598_2024_69975_MOESM1_ESM.docx]

**Appendix 1. Results**

**Classical Hodgkin Lymphoma (CHL)**

**Men**

A total of 36,522 cases of CHL were reported in men over 2000-2019. The majority of cases were NHWs (67.10%) and occurred between the ages of 20 and 29 (22.80%). Those between 40 and 49 years experienced the most significant decline, with an AAPC of -1.97% (-2.79, -1.28) (Table 3).

Of all the reported cases, 6,255 (17.13%) were Hispanics. The majority of registered individuals were between 20 and 29 years (22.81%). The overall ASIR per 100,000 population was 3.41 (3.31, 3.51). Cases between 70 and 79 years had the highest ASIR among other age groups (7.98 [7.34, 8.66]). There was a significant decrease in the incidence rate of Hispanic cases from 2000-2019 (-1.00%; [-1.49, -0.45]). Individuals aged 60-69 years experienced the most significant fall in incidence from 2000 to 2019 (-1.76% [-3.19, -0.06]) (Table 3).

NHBs constituted 10.96% of men with CHL. The majority of NHBs were in the age range of 20 to 29 years (23.98%). The overall ASIR per 100,000 population was 3.59 (3.47, 3.70), and cases between 30 and 39 years had the highest ASIR among other age groups (3.97 [3.72, 4.24]). The overall AAPC was -0.68% (-2.44, 1.16) (Table 3).

There were 24,498 reported cases of NHWs with CHL. The majority of the cases were between 20 and 29 years (22.00%). The overall ASIR per 100,000 population was 4.03 (3.98, 4.09), with cases between 20 and 29 years having the highest ASIR (5.13 [4.99, 5.27]). There was a significant decrease in overall ASIR over 2000-2019 in NHW men (AAPC: -1.14% [-1.88, -0.56]) (Table 3).

**Women**

Between 2000 and 2019, a total of 30,339 cases of CHL were reported. The majority of these cases were among NHWs (67.82%), with most falling between the ages of 20 and 29, representing 28.50% of the cases. Individuals between 20 and 29 years old had the highest reported ASIR (4.22 [4.13, 4.31]). Cases aged 60-69 years old experienced the most significant decline in ASIRs over 2000-2019 with an AAPC of -1.51% (-2.17, -0.79) (Table 3).

Among all reported cases, 4,992 (16.45%) were among Hispanic individuals. The majority of instances were between 20 and 29 years (31.91%). The overall ASIR per 100,000 population for this group was 2.43 (2.36, 2.50). Individuals between 70 and 79 years old had the highest ASIR compared to other age groups, with an ASIR of 4.80 (4.38, 5.26). There was a significant decrease in the ASIRs of cases between 50 and 59 years over the 2000-2019 period, with an AAPC of -2.52% (-4.23, -0.63) (Table 3).

NHBs comprised 10.88% of the instances of CHL in women. A noteworthy proportion of NHB instances were observed within the 20 to 29-year age group (28.48%). The overall ASIR per 100,000 population for NHB women was 2.54 (2.46, 2.63), with instances between 20 and 29 years old exhibiting the highest ASIR among other age groups (3.45 [3.23, 3.68]). Those between 20 and 29 years experienced the most substantial decline in ASIRs from 2000 to 2019, with an AAPC of -1.61% (-3.09, -0.13) (Table 3).

The majority of NHW instances occurred in the 20 to 29 (26.89%) age group. The overall ASIR per 100,000 population for this group was 3.29 (3.25, 3.34), with instances between 20 and 29 years old having the highest ASIR (5.43 [5.28, 5.57]). Over the 2000-2019 period, there was a significant decrease in the overall ASIRs for NHW women, with an AAPC of -0.81% (-1.21, -0.35). Individuals between 60 and 69 years old experienced the most substantial decline in ASIRs with an AAPC of -1.79% (-2.56, -0.99) from 2000 to 2019 (Table 3).

**Lymphocyte-rich/mixed cell/lymphocyte-depleted Hodgkin lymphoma (LR/MC/LD HL)**

**Men**

A total of 7,009 cases of LR/MC/LD HL were reported in men from 2000 to 2019. The majority of these cases were among NHWs (63.52%) and occurred in individuals aged 40 to 49 years (18.95%). Individuals between 70 and 79 had the highest ASIR compared to other age groups (1.16 [1.08, 1.24]). Those between 40 and 49 years had the most substantial decline in ASIRs over 2000-2019, with an AAPC of -5.24% (-8.46, -3.28) (Table 4).

Out of all the reported cases, 1,398 (19.95%) were among Hispanic individuals. The majority of cases were between 40 and 49 (21.82%). The overall ASIR per 100,000 population for this group was 0.79 (0.74, 0.84). Cases between 70 and 79 years had the highest ASIR among all age groups (1.86 [1.56, 2.20]). Those aged 50-59 years experienced the most significant decline in ASIR from 2000 to 2009 with an AAPC of -5.30% (-9.64, -1.37) (Table 4).

NHBs accounted for 11.88% of the cases of LR/MC/LD HL in men. The majority of NHB cases occurred in individuals aged 40 to 49 years (26.05%). The overall ASIR per 100,000 population for NHBs was 0.76 (0.70, 0.81), with cases between 40 and 49 years having the highest ASIR (0.95 [0.83, 1.09]). The overall AAPC for NHB over 2000-2019 was -4.30% (-7.06, -2.12) (Table 4).

The majority of NHW cases occurred in individuals aged 55 to 59 years (18.31%). The overall ASIR per 100,000 population for NHW men was 0.71 (0.69, 0.73), with cases between 70 and 79 years having the highest ASIR (1.16 [1.07, 1.26]). There was a significant decrease in the overall ASIR over the period of 2000 to 2019 in NHW men (AAPC: -4.20% [-4.82, -3.71]) (Table 4).

**Women**

Between 2000 and 2019, a total of 4,179 cases of LR/MC/LD HL were reported in women. The majority of these cases were among NHWs (66.09%) and between 70 and 79 years (16.85%). Individuals between 70 and 79 years old had the highest reported ASIR among other age groups (0.93 [0.87, 1.00]). Those over 80 years old had the most significant decline, with an AAPC of -5.66% (-8.27, -3.55) (Table 4).

Among all reported cases, 768 (18.38%) were among Hispanic individuals. The majority of cases were individuals between 60 and 69 years old (18.23%). The overall ASIR per 100,000 population for this group was 0.44 (0.41, 0.47). Individuals between 70 and 79 years had the highest ASIR compared to other age groups (1.34 [1.12, 1.59]). The overall AAPC for Hispanic women was -2.41% [-5.07, 0.31] (Table 4).

NHBs constituted 11.34% of the cases of LR/MC/LD HL in women. A significant portion of NHB cases fell within the 40 to 49-year age group (20.46%). The overall ASIR per 100,000 population for NHB women was 0.38 (0.34, 0.41), with cases between 70 and 79 years old having the highest ASIR among other age groups (0.59 [0.44, 0.78]). NHB women showed no significant changes in ASIRs over 2000-2019 (AAPC: -2.32% [-5.40, 0.39]) (Table 4).

The majority of NHW cases were in the 70 to 79 (21.83%) age group. The overall ASIR per 100,000 population was 0.39 (0.38, 0.41), with cases between 70 and 79 years old having the highest ASIR at 0.96 (0.88, 1.04). From 2000 to 2019, there was a significant decrease in the overall ASIR for NHW women, with an AAPC of -3.57% (-4.28, -2.96) (Table 4).

**Nodular sclerosis Hodgkin lymphoma (NSHL)**

**Men**

A total of 17,850 cases of NSHL were reported in men between 2000 and 2019. The majority of these cases were among NHWs (69.06%), and the most considerable portion of them occurred in individuals aged 20 to 29 years (30.45%). Among men with NSHL, those between 20 and 29 years had the highest ASIR (2.55 [2.48, 2.62]). Individuals aged 40-49 showed the largest decrease with an AAPC of -3.07% (-4.06, -2.21) (Table 5).

Out of all the reported cases, 2,548 (14.27%) were among Hispanic individuals. The majority of cases were between 20 and 29 years (32.10%). The overall ASIR per 100,000 population for this group was 1.25 (1.19, 1.30). Cases aged 70-79 years had the highest ASIR among all age groups (2.32 [1.98, 2.70]). There was a significant decrease in ASIR from 2000-2019, with an AAPC of -2.89% (-4.42, -0.95). Notably, individuals between 60 and 69 had the most substantial fall in ASIRs (AAPC: -5.82% [-8.11, -3.49]) (Table 5).

NHBs accounted for 9.42% of the cases of NSHL in men. A majority of NHB cases occurred in individuals between 20 and 29 years (32.88%). The overall ASIR per 100,000 population for NHBs was 1.46 (1.38, 1.53), and cases aged 20-29 years had the highest ASIR among all age groups (2.09 [1.92, 2.27]). Although the overall AAPC was not significant, those aged 30-39 years had the most significant decrease in ASIRs (AAPC: -3.11% [-5.45, -1.00]) (Table 5).

The majority of NHWs were between 20 and 29 years old (29.21%). The overall ASIR per 100,000 population for NHW men was 2.16 (2.13, 2.20), with cases between 20 and 29 years old having the highest ASIR (3.54 [3.43, 3.66]). The overall AAPC for NHW between 2000 and 2019 was -2.38 (-3.12, -1.75) (Table 5).

**Women**

Between 2000 and 2019, a total of 17,684 cases of NSHL were documented in women. The majority of these cases were observed among NHWs, accounting for 72.10%. Notably, the age group between 20 and 29 years old had the highest number of reported cases, comprising 37.02% of the total. Additionally, this age group exhibited the highest ASIR at 3.20 (3.12, 3.27). A noteworthy trend was observed in individuals aged 60-69, who experienced a substantial decline in ASIRs over the period 2000-2019, with an AAPC of -3.35% (-4.68, -2.01) (Table 5).

Hispanics constituted 15.19% of the cases of NSHL in women, with a large proportion in the 20 to 29-year age group (42.81%). The overall ASIR per 100,000 population for Hispanic women was 1.17 (1.12, 1.20), with cases in the 20-29 age group having the highest ASIR among other age groups (2.06 [1.95, 2.19]). The overall AAPC in this group was -2.64% (-3.56, -1.71). Among the age groups, those between 60 and 69 had the most significant drop in ASIRs with an AAPC of -5.57% (-9.48, -1.57) (Table 5).

Among all reported cases, 10.10% were NHBs. The majority of cases were between 20 and 29 years old (37.18%). The overall ASIR per 100,000 population was 1.36 (1.29, 1.42), with cases between 20 and 29 years having the highest ASIR (2.44 [2.26, 2.63]). The overall AAPC for this group was -1.89% (-2.81, -0.98) (Table 5).

The majority of NHW cases were in the 20 to 29 age group (34.90%). The overall ASIR per 100,000 population was 2.10 (2.06, 2.14), with cases in the 20-29 age group having the highest ASIR (4.22 [4.10, 4.35]). NHWs experienced a significant drop in ASIRs between 2000 and 2019 (AAPC: -1.99% [-2.86, -1.40]) (Table 5).

**Classical Hodgkin lymphoma not otherwise specified (CHL-NOS)**

**Men**

A total of 11,663 cases of CHL-NOS were reported in men over 2000-2019. The majority of the cases were NHWs (62.55%) and between 50 and 59 years (17.23%). Cases over 80 had the highest reported ASIR per 100,000 population (2.40 [2.24, 2.56]). Additionally, the same group exhibited the largest increase in ASIRs between 2000 and 2019 (AAPC: 6.42% [0.83, 13.02]) (Table 6).

Of all the reported cases, 2,309 (19.80%) were Hispanics. The majority of cases were in the 30-39 age group (17.67%). The overall ASIR per 100,000 population was 1.37 (1.31, 1.44). Cases between 70 and 79 years had the highest ASIR among other age groups (3.80 [3.37, 4.28]). Hispanic men experienced a significant increase in ASIRs from 2000-2019 (AAPC: 3.01% [1.92, 4.43]) (Table 6).

NHBs consisted of 12.76% of the men with CHL-NOS. The majority of NHBs were between 40 and 49 years old (22.65%). The overall ASIR per 100,000 population was 1.37 (1.30, 1.45), and the cases between 50 and 59 years had the highest ASIR among other age groups (1.57 [1.40, 1.76]). NHBs experienced a substantial increase in ASIRs with an AAPC at 2.42% (1.36, 3.68) with cases in the 20-29 age groups having the highest AAPC compared to the other age groups (AAPC: 3.58% [0.29, 8.21]) (Table 6).

There were 7,295 reported cases of NHWs. The majority of the cases were in the 60-69 age group years (16.60%). The overall ASIR per 100,000 population was 1.16 (1.13, 1.19) with cases over 80 years having the highest ASIR (2.43 [2.25, 2.62]). There was an increase in AAPC (2.65% [2.09, 3.27]) with individuals over 80 years having the highest increase in ASIRs compared to the other age groups with an AAPC of 5.56% (0.46, 11.31) (Table 6).

**Women**

Between 2000 and 2019, a total of 8,476 cases of CHL-NOS were reported in women. The majority of these cases were among NHWs (64.72%), and most of them were in the 20-29 age group (18.66%). Cases over 80 years had the highest ASIR (1.46 [1.37, 1.56]). Notably cases between 30 and 39 years had the highest AAPC (4.98% [4.26, 5.87]) (Table 6).

Among all reported cases, 1,538 (18.15%) were among Hispanic individuals. The majority of reported cases were between 20 and 29 years (22.56%). This group's overall ASIR per 100,000 population was 0.82 (0.78, 0.87). Cases among individuals between 70 and 79 years had the highest ASIR compared to other age groups (2.24 [1.96, 2.56]). The AAPC for Hispanics was 3.19% (2.20, 4.52), and those aged 20-29 years experienced the greatest increase in ASIRs compared to other age groups (AAPC: 6.83% [4.91, 9.67]) (Table 6).

NHBs constituted 12.27% of the cases of CHL-NOS in women. A significant portion of NHB cases fell between the 20- and 29-years age group (20.19%). The overall ASIR per 100,000 population for NHB women was 0.81 (0.76, 0.86), with cases over 80 years old having the highest ASIR among other age groups (1.04 [0.77, 1.38]). NHBs in the 70-79 age group had the highest AAPC (5.76% [3.16, 9.91]) (Table 6).

The majority of NHW cases were between 20 and 29 years (16.67%) age group. The overall ASIR per 100,000 population for this group was 0.81 (0.78, 0.83), with cases over 80 years old having the highest ASIR (1.49 [1.39, 1.61]). Over the 2000-2019 period, there was a significant increase in ASIRs for NHW women with an AAPC at 3.81% (2.86, 5.09) (Table 6).

**Nodular lymphocyte prominent Hodgkin lymphoma (NLPHL)**

**Men**

A total of 2,627 cases of NLPHL were reported in men between 2000 and 2019. Most of these cases were among NHWs (64.10%) and occurred in cases aged 40 to 49 years (20.33%). Those in the 60-69 age group had the highest ASIR (0.30 [0.27, 0.33]) (Table 7).

Out of all the reported cases, 12.22% were among Hispanic individuals. Most cases were between 20 and 29 years old (26.17%). This group's overall ASIR per 100,000 population was 0.15 (0.14, 0.17). Cases between 50 and 59 years had the highest ASIR among all age groups (0.20 [0.15, 0.26]). Hispanics experienced an increase in ASIRs from 2000 to 2019 with an AAPC of 3.57% (1.48, 6.56) (Table 7).

NHBs accounted for 18.96% of the cases of NLPHL. Most NHB cases occurred between 40 and 49 years (26.10%). The overall ASIR per 100,000 population was 0.44 (0.41, 0.49), and cases in the 40-49 age group had the highest ASIR (0.57 [0.47, 0.67]). NHBs had a substantial increase in ASIRs with an AAPC at 5.98% (3.93, 8.93) (Table 7).

Most NHWs were individuals aged 50 to 59 years (19.77%). The overall ASIR per 100,000 population for NHW men was 0.27 (0.26, 0.28), with cases between 60 and 69 years having the highest ASIR (0.31 [0.27, 0.35]). NHW men exhibited a significant increase in ASIRs with an AAPC at 4.87% (4.02, 5.84) (Table 7).

**Women**

Between 2000 and 2019, a total of 1,436 cases of NLPHL were reported in women. The majority of these cases were among NHWs (49.23%) and between 50 and 59 years (21.24%). Cases between 60 and 69 years had the highest ASIR per 100,000 population compared to other age groups (0.20 [0.18, 0.23]) (Table 7).

Among all reported cases, 12.33% were among Hispanic individuals. The majority of cases were individuals between 40 and 49 years (24.86%). The overall ASIR per 100,000 population was 0.09 (0.08, 0.11). There was a significant increase in ASIRs between 2000 and 2019 (AAPC: 4.20% [0.71, 9.74]) (Table 7).

NHBs constituted 33.98 % of the cases of NLPHL in women. A large portion of NHB cases were between 40 and 49-year age group (24.39%). The overall ASIR per 100,000 population for NHB women was 0.38 (0.35, 0.42), with cases between 60 and 69 years old having the highest ASIR among other age groups (0.49 [0.39, 0.62]). NHBS experienced a significant change in ASIRs with an AAPC of 6.14% (3.96, 9.31) (Table 7).

The majority of NHW cases were in the 60 to 69 age group (22.63%). The overall ASIR per 100,000 population was 0.10 (0.09, 0.11), with cases between 70 and 79 years having the highest ASIR (0.19 [0.16, 0.23]). Over the 2000-2019, there was an increase in ASIRs in NHWs with an AAPC of 6.25% (4.73, 8.28) (Table 7).
