# Supplementary material for: Adult Hodgkin lymphoma incidence trends in the United States from 2000 to 2020
Source: Sci Rep. 2024 Sep 3;14:20500. doi: 10.1038/s41598-024-69975-3 (PMC11372180; doi:10.1038/s41598-024-69975-3)
Supplement: Supplementary file 2 — Supplementary Information 2. [file 41598_2024_69975_MOESM2_ESM.docx]

| **All race/ethnicities** | | | | | | |
| --- | --- | --- | --- | --- | --- | --- |
| **Age group (years)** | **Men** | | | **Women** | | |
|  | **Case (%)** | **Delayed ASIR (95% CI)** | **AAPC (95% CI)** | **Case (%)** | **Delayed ASIR (95% CI)** | **AAPC (95% CI)** |
| 20+ | 9957 (55.62) | 3.56 (3.49, 3.63) | -1.27 (-2.2, -0.92) | 7946 (44.38) | 2.73 (2.67, 2.79) | -1.11 (-1.75, -0.8) |
| 20-29 | 2243 (12.53) | 3.98 (3.82, 4.15) | -0.48 (-1.01, 0.07) | 2137 (11.94) | 3.96 (3.8, 4.14) | -1.47 (-2.53, -0.99) |
| 30-39 | 1826 (10.20) | 3.4 (3.24, 3.56) | -1.11 (-1.62, -0.62) | 1593 (8.90) | 3 (2.85, 3.15) | -0.75 (-1.22, -0.3) |
| 40-49 | 1409 (7.87) | 2.91 (2.76, 3.07) | -2.23 (-5.97, -1.29) | 1031 (5.76) | 2.09 (1.97, 2.22) | -0.25 (-0.96, 0.45) |
| 50-59 | 1567 (8.75) | 3.15 (2.99, 3.31) | -0.14 (-0.6, 0.34) | 920 (5.14) | 1.79 (1.67, 1.91) | -0.39 (-0.94, 0.19) |
| 60-69 | 1410 (7.88) | 3.63 (3.45, 3.83) | -1.22 (-1.98, -0.37) | 974 (5.44) | 2.27 (2.13, 2.42) | -0.87 (-1.5, -0.19) |
| 70-79 | 1017 (5.68) | 4.85 (4.55, 5.15) | -1.71 (-3.66, -0.87) | 841 (4.70) | 3.29 (3.07, 3.52) | -2.07 (-8.69, -1.12) |
| 80+ | 485 (2.71) | 4.65 (4.25, 5.09) | -1.9 (-6.73, -0.61) | 450 (2.51) | 2.72 (2.47, 2.99) | -2.19 (-7.08, -1.06) |
| **Hispanic** | | | | | | |
| **Age groups** | **Men** | | | **Women** | | |
|  | **Case (%)** | **Delayed ASIR (95% CI)** | **AAPC (95% CI)** | **Case (%)** | **Delayed ASIR (95% CI)** | **AAPC (95% CI)** |
| 20+ | 1905 (56.36) | 3.36 (3.2, 3.53) | -0.72 (-1.18, -0.21) | 1475 (43.64) | 2.36 (2.24, 2.49) | -1.27 (-4.7, -0.26) |
| 20-29 | 419 (12.40) | 2.52 (2.28, 2.77) | -0.52 (-3.71, 0.78) | 454 (13.43) | 2.92 (2.66, 3.2) | 0.35 (-0.65, 1.44) |
| 30-39 | 353 (10.44) | 2.29 (2.05, 2.54) | -0.5 (-1.63, 0.68) | 290 (8.58) | 2.03 (1.8, 2.28) | 0.5 (-0.51, 1.62) |
| 40-49 | 302 (8.93) | 2.36 (2.1, 2.64) | -1.49 (-2.85, -0.05) | 178 (5.27) | 1.42 (1.22, 1.64) | -0.2 (-2.19, 2.09) |
| 50-59 | **295** (8.73) | 3.04 (2.7, 3.41) | -0.94 (-2.26, 0.61) | 163 (4.82) | 1.65 (1.4, 1.92) | -1.8 (-3.56, 0.2) |
| 60-69 | 267 (7.90) | 4.81 (4.25, 5.43) | 1.62 (-3.87, 10.79) | 172 (5.09) | 2.77 (2.37, 3.21) | -0.78 (-2.54, 1.37) |
| 70-79 | 193 (5.71) | 7.73 (6.66, 8.91) | -0.28 (-1.92, 1.71) | 138 (4.08) | 4.28 (3.59, 5.06) | -3.47 (-9.78, -1.35) |
| 80+ | 76 (2.25) | 6.43 (5.07, 8.05) | -1.76 (-3.51, 0.38) | 80 (2.37) | 4.23 (3.36, 5.27) | -1.05 (-3.11, 1.47) |
| **NHB** | | | | | | |
| **Age groups** | **Men** | | | **Women** | | |
|  | **Case (%)** | **Delayed ASIR (95% CI)** | **AAPC (95% CI)** | **Case (%)** | **Delayed ASIR (95% CI)** | **AAPC (95% CI)** |
| 20+ | 1219 (53.84) | 3.96 (3.74, 4.2) | 0.17 (-0.58, 0.97) | 1905 (46.16) | 3.01 (2.83, 3.2) | 0.68 (0.08, 1.32) |
| 20-29 | 288 (12.72) | 3.8 (3.37, 4.27) | 0.09 (-1.67, 2.03) | 239 (10.56) | 3.22 (2.82, 3.65) | -1.11 (-2.49, 0.28) |
| 30-39 | 262 (11.57) | 4.28 (3.77, 4.83) | -0.15 (-1.39, 1.11) | 243 (10.73) | 3.66 (3.21, 4.15) | 1.12 (-0.11, 2.44) |
| 40-49 | 231 (10.20) | 4.27 (3.74, 4.86) | 0.22 (-0.86, 1.31) | 192 (8.48) | 3.13 (2.7, 3.6) | 1.59 (-0.31, 3.65) |
| 50-59 | 230 (10.16) | 4.23 (3.7, 4.81) | 0.7 (-0.4, 1.96) | 159 (7.02) | 2.58 (2.19, 3.01) | 1.06 (-0.03, 2.3) |
| 60-69 | 127 (5.61) | 3.38 (2.82, 4.03) | 0.31 (-1.97, 3.11) | 120 (5.30) | 2.55 (2.12, 3.05) | -10.13 (-20.66, 0.58) |
| 70-79 | 62 (2.74) | 3.7 (2.83, 4.76) | -0.09 (-2.07, 2.19) | 70 (3.09) | 2.82 (2.2, 3.57) | 1.78 (-0.11, 4.13) |
| 80+ | 19 (0.84) | 2.58 (1.55, 4.03) | N/A | 22 (0.97) | 1.54 (0.96, 2.34) | -5.71 (-38.94, -0.46) |
| **NHW** | | | | | | |
| **Age groups** | **Men** | | | **Women** | | |
|  | **Case (%)** | **Delayed ASIR (95% CI)** | **AAPC (95% CI)** | **Case (%)** | **Delayed ASIR (95% CI)** | **AAPC (95% CI)** |
| 20+ | 6239 (55.91) | 4.01 (3.9, 4.11) | -1.29 (-4.02, -0.9) | 4920 (44.09) | 3.15 (3.06, 3.25) | -1.27 (-2.85, -0.89) |
| 20-29 | 1364 (12.22) | 5.17 (4.9, 5.45) | 1.17 (-1, 4.56) | 1258 (11.27) | 5 (4.72, 5.28) | -1.47 (-4.25, -0.94) |
| 30-39 | 1081 (9.69) | 4.12 (3.88, 4.38) | -1.09 (-1.65, -0.57) | 951 (8.52) | 3.71 (3.48, 3.96) | -1 (-1.55, -0.48) |
| 40-49 | 804 (7.20) | 3.22 (3, 3.45) | -1 (-1.54, -0.5) | 595 (5.33) | 2.41 (2.22, 2.61) | -0.17 (-1.01, 0.6) |
| 50-59 | 970 (8.69) | 3.21 (3.01, 3.42) | -0.1 (-0.53, 0.33) | 551 (4.94) | 1.82 (1.67, 1.98) | -0.38 (-1.06, 0.3) |
| 60-69 | 943 (8.45) | 3.61 (3.38, 3.85) | -1.36 (-2.3, -0.34) | 636 (5.70) | 2.27 (2.1, 2.46) | -1.18 (-2.03, -0.28) |
| 70-79 | 714 (6.40) | 4.75 (4.41, 5.12) | -2.25 (-3.23, -1.49) | 599 (5.37) | 3.39 (3.12, 3.68) | -1.11 (-2.24, 0.01) |
| 80+ | 363 (3.25) | 4.76 (4.28, 5.27) | -1.99 (-8.18, -0.67) | 330 (2.96) | 2.78 (2.48, 3.1) | -1.15 (-3.06, 0.64) |

**Table S1.** Counts and age-standardized rate of Hodgkin lymphoma cancer incidence per 100,000 and average annual percent change from 2015 to 2019 in the United States, by age, sex, and race.

**Abbreviations:** NHW: Non-Hispanic White; NHB: Non-Hispanic Black; ASIR: Age-standardized incidence rate; CI: Confidence interval, AAPC: Average annual percent change.

**Table S2**. Results of the tests of incidental trends for adult Hodgkin lymphoma incidence rate over 2000-2019 in the United States.

| **Race/ ethnicities** | **sex** | **subtypes** | **Race/ ethnicities** | **sex** | **subtype** | **P value** |
| --- | --- | --- | --- | --- | --- | --- |
| **Cohort 1** | | | **Cohort 2** | | |  |
| Hispanic | Female | CHL-NOS | NHB | Female | CHL-NOS | 0.94 |
| Hispanic | Female | CHL-NOS | NHW | Female | CHL-NOS | 0.86 |
| NHB | Female | CHL-NOS | NHW | Female | CHL-NOS | 0.82 |
| All | Male | CHL-NOS | NHW | Male | CHL-NOS | 0.45 |
| Hispanic | Male | CHL-NOS | NHB | Male | CHL-NOS | 0.45 |
| Hispanic | Both | CHL-NOS | NHB | Both | CHL-NOS | 0.87 |
| Hispanic | Female | CHL | NHB | Female | CHL | 0.37 |
| All | Male | CHL | NHB | Male | CHL | 0.18 |
| Hispanic | Male | CHL | NHB | Male | CHL | 0.07 |
| Hispanic | Both | CHL | NHB | Both | CHL | 0.08 |
| All | Female | LR/MC/LD | NHB | Female | LR/MC/LD | 0.05 |
| Hispanic | Female | LR/MC/LD | NHB | Female | LR/MC/LD | 0.11 |
| All | Male | LR/MC/LD | NHW | Male | LR/MC/LD | 0.62 |
| Hispanic | Male | LR/MC/LD | NHB | Male | LR/MC/LD | 0.38 |
| Hispanic | Female | NLPHL | NHW | Female | NLPHL | 0.25 |
| All | Both | NLPHL | NHW | Both | NLPHL | 0.74 |
| NHW | Female | NLPHL | NHB | Male | NLPHL | 0.16 |
| NHB | Female | NLPHL | NHB | Both | NLPHL | 0.35 |
| NHB | Male | NLPHL | NHB | Both | NLPHL | 0.08 |
| All | Female | NSHL | All | Both | NSHL | 0.05 |
| Hispanic | Female | NSHL | Hispanic | Male | NSHL | 0.07 |
| Hispanic | Female | NSHL | Hispanic | Both | NSHL | 0.21 |
| NHB | Female | NSHL | NHB | Male | NSHL | 0.33 |
| NHB | Female | NSHL | NHB | Both | NSHL | 0.85 |
| NHB | Male | NSHL | NHB | Both | NSHL | 0.08 |
| NHW | Female | NSHL | NHW | Male | NSHL | 0.2 |
| NHW | Female | NSHL | NHW | Both | NSHL | 0.24 |
| NHW | Male | NSHL | NHW | Both | NSHL | 0.06 |

**Abbreviations**: NHW: Non-Hispanic White; NHB: Non-Hispanic Black; CHL-NOS: Classical Hodgkin Lymphoma-Not Otherwise Specified; CHL: Classical Hodgkin Lymphoma; LR/MC/LD: Lymphocyte-Rich/ Mixed Cellular/ Lymphocyte depleted; NLPHL: Nodular Lymphocyte Predominant Hodgkin Lymphoma; NSHL: Nodular Sclerosis Hodgkin Lymphoma.

**Table S3**. Results of the tests of parallelism for adult Hodgkin lymphoma incidence rate over 2000-2019 in the United States.

| **Race/ ethnicities** | **sex** | **subtypes** | **Race/ ethnicities** | **sex** | **subtype** | **P value** |
| --- | --- | --- | --- | --- | --- | --- |
| **Cohort 1** | | | **Cohort 2** | | |  |
| Hispanic | Female | CHL-NOS | Hispanic | Female | NLPHL | 0.38 |
| Hispanic | Female | CHL | Hispanic | Female | HL | 0.13 |
| Hispanic | Female | CHL | Hispanic | Female | NSHL | 0.08 |
| NHB | Female | LR/MC/LD | NHB | Female | NSHL | 0.24 |
| Hispanic | Male | CHL-NOS | Hispanic | Male | NLPHL | 0.62 |
| NHB | Male | LR/MC/LD | NHB | Male | NSHL | 0.18 |
| Hispanic | Both | CHL-NOS | Hispanic | Both | NLPHL | 0.36 |
| All | Female | CHL-NOS | Hispanic | Female | CHL-NOS | 0.76 |
| All | Female | CHL-NOS | NHB | Female | CHL-NOS | 0.65 |
| All | Female | CHL-NOS | NHW | Female | CHL-NOS | 0.26 |
| Hispanic | Female | CHL-NOS | NHB | Female | CHL-NOS | 0.75 |
| Hispanic | Female | CHL-NOS | NHW | Female | CHL-NOS | 0.72 |
| NHB | Female | CHL-NOS | NHW | Female | CHL-NOS | 0.66 |
| All | Male | CHL-NOS | Hispanic | Male | CHL-NOS | 0.18 |
| All | Male | CHL-NOS | NHB | Male | CHL-NOS | 0.6 |
| All | Male | CHL-NOS | NHW | Male | CHL-NOS | 0.78 |
| Hispanic | Male | CHL-NOS | NHB | Male | CHL-NOS | 0.26 |
| Hispanic | Male | CHL-NOS | NHW | Male | CHL-NOS | 0.51 |
| NHB | Male | CHL-NOS | NHW | Male | CHL-NOS | 0.69 |
| All | Both | CHL-NOS | Hispanic | Both | CHL-NOS | 0.85 |
| All | Both | CHL-NOS | NHB | Both | CHL-NOS | 0.5 |
| All | Both | CHL-NOS | NHW | Both | CHL-NOS | 0.75 |
| Hispanic | Both | CHL-NOS | NHB | Both | CHL-NOS | 0.73 |
| Hispanic | Both | CHL-NOS | NHW | Both | CHL-NOS | 0.9 |
| NHB | Both | CHL-NOS | NHW | Both | CHL-NOS | 0.69 |
| All | Female | CHL | Hispanic | Female | CHL | 0.05 |
| All | Female | CHL | NHW | Female | CHL | 0.57 |
| Hispanic | Female | CHL | NHB | Female | CHL | 0.6 |
| Hispanic | Female | CHL | NHW | Female | CHL | 0.08 |
| All | Male | CHL | NHB | Male | CHL | 0.12 |
| All | Male | CHL | NHW | Male | CHL | 0.39 |
| Hispanic | Male | CHL | NHB | Male | CHL | 0.25 |
| Hispanic | Male | CHL | NHW | Male | CHL | 0.14 |
| Hispanic | Both | CHL | NHB | Both | CHL | 0.28 |
| All | Female | HL | Hispanic | Female | HL | 0.08 |
| Hispanic | Female | HL | NHB | Female | HL | 0.07 |
| Hispanic | Female | HL | NHW | Female | HL | 0.07 |
| All | Male | HL | Hispanic | Male | HL | 0.28 |
| All | Male | HL | NHW | Male | HL | 0.19 |
| Hispanic | Male | HL | NHB | Male | HL | 0.09 |
| Hispanic | Male | HL | NHW | Male | HL | 0.35 |
| All | Female | LR/MC/LD | Hispanic | Female | LR/MC/LD | 0.17 |
| All | Female | LR/MC/LD | NHW | Female | LR/MC/LD | 0.68 |
| Hispanic | Female | LR/MC/LD | NHB | Female | LR/MC/LD | 0.36 |
| Hispanic | Female | LR/MC/LD | NHW | Female | LR/MC/LD | 0.42 |
| All | Male | LR/MC/LD | Hispanic | Male | LR/MC/LD | 0.59 |
| All | Male | LR/MC/LD | NHB | Male | LR/MC/LD | 0.29 |
| All | Male | LR/MC/LD | NHW | Male | LR/MC/LD | 0.78 |
| Hispanic | Male | LR/MC/LD | NHB | Male | LR/MC/LD | 0.53 |
| Hispanic | Male | LR/MC/LD | NHW | Male | LR/MC/LD | 0.63 |
| NHB | Male | LR/MC/LD | NHW | Male | LR/MC/LD | 0.39 |
| All | Both | LR/MC/LD | Hispanic | Both | LR/MC/LD | 0.3 |
| All | Both | LR/MC/LD | NHW | Both | LR/MC/LD | 0.84 |
| Hispanic | Both | LR/MC/LD | NHB | Both | LR/MC/LD | 0.16 |
| Hispanic | Both | LR/MC/LD | NHW | Both | LR/MC/LD | 0.5 |
| All | Female | NLPHL | Hispanic | Female | NLPHL | 0.13 |
| All | Female | NLPHL | NHB | Female | NLPHL | 0.82 |
| All | Female | NLPHL | NHW | Female | NLPHL | 0.89 |
| Hispanic | Female | NLPHL | NHB | Female | NLPHL | 0.35 |
| Hispanic | Female | NLPHL | NHW | Female | NLPHL | 0.27 |
| NHB | Female | NLPHL | NHW | Female | NLPHL | 0.94 |
| All | Male | NLPHL | Hispanic | Male | NLPHL | 0.61 |
| All | Male | NLPHL | NHB | Male | NLPHL | 0.42 |
| All | Male | NLPHL | NHW | Male | NLPHL | 0.66 |
| Hispanic | Male | NLPHL | NHB | Male | NLPHL | 0.05 |
| Hispanic | Male | NLPHL | NHW | Male | NLPHL | 0.25 |
| NHB | Male | NLPHL | NHW | Male | NLPHL | 0.18 |
| All | Both | NLPHL | Hispanic | Both | NLPHL | 0.09 |
| All | Both | NLPHL | NHB | Both | NLPHL | 0.25 |
| All | Both | NLPHL | NHW | Both | NLPHL | 0.57 |
| Hispanic | Both | NLPHL | NHW | Both | NLPHL | 0.08 |
| NHB | Both | NLPHL | NHW | Both | NLPHL | 0.12 |
| All | Female | NSHL | Hispanic | Female | NSHL | 0.07 |
| All | Female | NSHL | NHB | Female | NSHL | 0.26 |
| Hispanic | Female | NSHL | NHB | Female | NSHL | 0.25 |
| Hispanic | Female | NSHL | NHW | Female | NSHL | 0.12 |
| NHB | Female | NSHL | NHW | Female | NSHL | 0.49 |
| All | Male | NSHL | Hispanic | Male | NSHL | 0.13 |
| All | Male | NSHL | NHW | Male | NSHL | 0.15 |
| Hispanic | Male | NSHL | NHB | Male | NSHL | 0.36 |
| Hispanic | Male | NSHL | NHW | Male | NSHL | 0.06 |
| Hispanic | Both | NSHL | NHB | Both | NSHL | 0.48 |
| All | Female | CHL-NOS | All | Male | CHL-NOS | 0.2 |
| All | Female | CHL-NOS | All | Both | CHL-NOS | 0.15 |
| All | Male | CHL-NOS | All | Both | CHL-NOS | 0.33 |
| Hispanic | Female | CHL-NOS | Hispanic | Male | CHL-NOS | 0.5 |
| Hispanic | Female | CHL-NOS | Hispanic | Both | CHL-NOS | 0.4 |
| Hispanic | Male | CHL-NOS | Hispanic | Both | CHL-NOS | 0.61 |
| NHB | Female | CHL-NOS | NHB | Male | CHL-NOS | 0.27 |
| NHB | Female | CHL-NOS | NHB | Both | CHL-NOS | 0.25 |
| NHB | Male | CHL-NOS | NHB | Both | CHL-NOS | 0.17 |
| NHW | Female | CHL-NOS | NHW | Male | CHL-NOS | 0.45 |
| NHW | Female | CHL-NOS | NHW | Both | CHL-NOS | 0.44 |
| NHW | Male | CHL-NOS | NHW | Both | CHL-NOS | 0.22 |
| All | Female | CHL | All | Male | CHL | 0.81 |
| All | Female | CHL | All | Both | CHL | 0.51 |
| All | Male | CHL | All | Both | CHL | 0.81 |
| Hispanic | Female | CHL | Hispanic | Male | CHL | 0.42 |
| Hispanic | Female | CHL | Hispanic | Both | CHL | 0.13 |
| Hispanic | Male | CHL | Hispanic | Both | CHL | 0.35 |
| NHB | Female | CHL | NHB | Male | CHL | 0.78 |
| NHB | Female | CHL | NHB | Both | CHL | 0.43 |
| NHB | Male | CHL | NHB | Both | CHL | 0.54 |
| NHW | Female | CHL | NHW | Male | CHL | 0.68 |
| NHW | Female | CHL | NHW | Both | CHL | 0.93 |
| NHW | Male | CHL | NHW | Both | CHL | 0.82 |
| All | Female | HL | All | Male | HL | 0.89 |
| All | Female | HL | All | Both | HL | 0.33 |
| All | Male | HL | All | Both | HL | 0.94 |
| Hispanic | Female | HL | Hispanic | Male | HL | 0.44 |
| Hispanic | Female | HL | Hispanic | Both | HL | 0.16 |
| Hispanic | Male | HL | Hispanic | Both | HL | 0.43 |
| NHB | Female | HL | NHB | Male | HL | 0.74 |
| NHB | Female | HL | NHB | Both | HL | 0.81 |
| NHB | Male | HL | NHB | Both | HL | 0.59 |
| NHW | Female | HL | NHW | Male | HL | 1 |
| NHW | Female | HL | NHW | Both | HL | 0.96 |
| NHW | Male | HL | NHW | Both | HL | 0.99 |
| All | Female | LR/MC/LD | All | Male | LR/MC/LD | 0.14 |
| All | Female | LR/MC/LD | All | Both | LR/MC/LD | 0.32 |
| All | Male | LR/MC/LD | All | Both | LR/MC/LD | 0.06 |
| Hispanic | Female | LR/MC/LD | Hispanic | Male | LR/MC/LD | 0.2 |
| Hispanic | Male | LR/MC/LD | Hispanic | Both | LR/MC/LD | 0.18 |
| NHB | Female | LR/MC/LD | NHB | Male | LR/MC/LD | 0.49 |
| NHB | Female | LR/MC/LD | NHB | Both | LR/MC/LD | 0.51 |
| NHB | Male | LR/MC/LD | NHB | Both | LR/MC/LD | 0.24 |
| NHW | Female | LR/MC/LD | NHW | Male | LR/MC/LD | 0.09 |
| NHW | Male | LR/MC/LD | NHW | Both | LR/MC/LD | 0.37 |
| All | Female | NLPHL | All | Male | NLPHL | 0.41 |
| All | Female | NLPHL | All | Both | NLPHL | 0.33 |
| All | Male | NLPHL | All | Both | NLPHL | 0.3 |
| Hispanic | Female | NLPHL | Hispanic | Male | NLPHL | 0.77 |
| Hispanic | Female | NLPHL | Hispanic | Both | NLPHL | 0.64 |
| Hispanic | Male | NLPHL | Hispanic | Both | NLPHL | 0.85 |
| NHB | Female | NLPHL | NHB | Male | NLPHL | 0.92 |
| NHB | Female | NLPHL | NHB | Both | NLPHL | 0.58 |
| NHB | Male | NLPHL | NHB | Both | NLPHL | 0.5 |
| NHW | Female | NLPHL | NHW | Male | NLPHL | 0.17 |
| NHW | Female | NLPHL | NHW | Both | NLPHL | 0.21 |
| NHW | Male | NLPHL | NHW | Both | NLPHL | 0.53 |
| All | Female | NSHL | All | Male | NSHL | 0.37 |
| All | Female | NSHL | All | Both | NSHL | 0.24 |
| All | Male | NSHL | All | Both | NSHL | 0.46 |
| Hispanic | Female | NSHL | Hispanic | Male | NSHL | 0.21 |
| Hispanic | Female | NSHL | Hispanic | Both | NSHL | 0.27 |
| Hispanic | Male | NSHL | Hispanic | Both | NSHL | 0.41 |
| NHB | Female | NSHL | NHB | Male | NSHL | 0.64 |
| NHB | Female | NSHL | NHB | Both | NSHL | 0.91 |
| NHB | Male | NSHL | NHB | Both | NSHL | 0.33 |
| NHW | Female | NSHL | NHW | Male | NSHL | 0.44 |
| NHW | Female | NSHL | NHW | Both | NSHL | 0.27 |
| NHW | Male | NSHL | NHW | Both | NSHL | 0.44 |

**Abbreviations**: NHW: Non-Hispanic White; NHB: Non-Hispanic Black ; CHL-NOS: Classical Hodgkin Lymphoma-Not Otherwise Specified; CHL: Classical Hodgkin Lymphoma; LR/MC/LD: Lymphocyte-Rich/ Mixed Cellular/ Lymphocyte depleted; NLPHL: Nodular Lymphocyte Predominant Hodgkin Lymphoma; NSHL: Nodular Sclerosis Hodgkin Lymphoma.


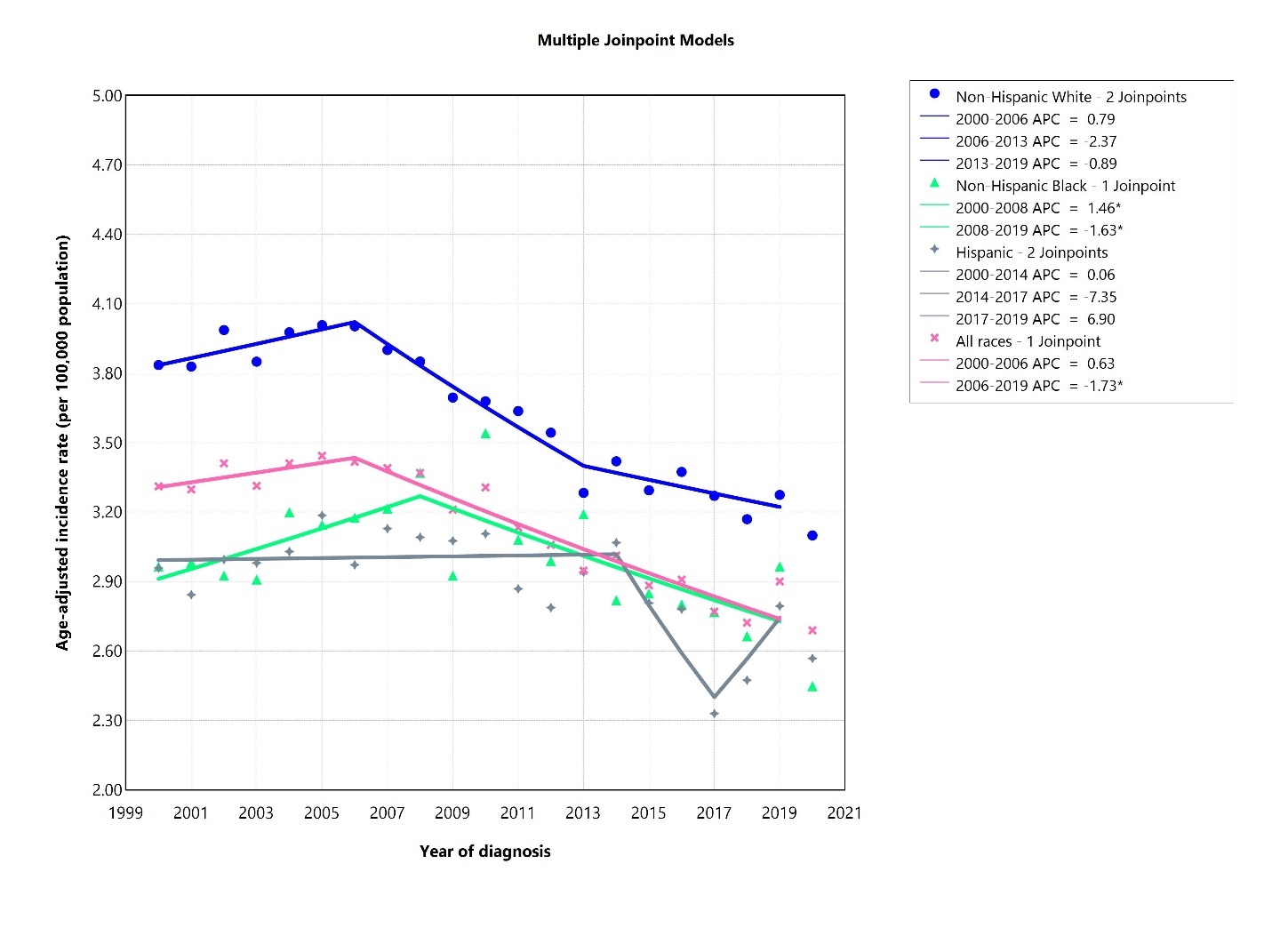
**Figure S1.** Age-adjusted incidence rate of adult classical Hodgkin lymphoma over 2000-2019 and in 2020 in the United States, by race. APC: annual percent change. * Represent p-value less than 0.05.


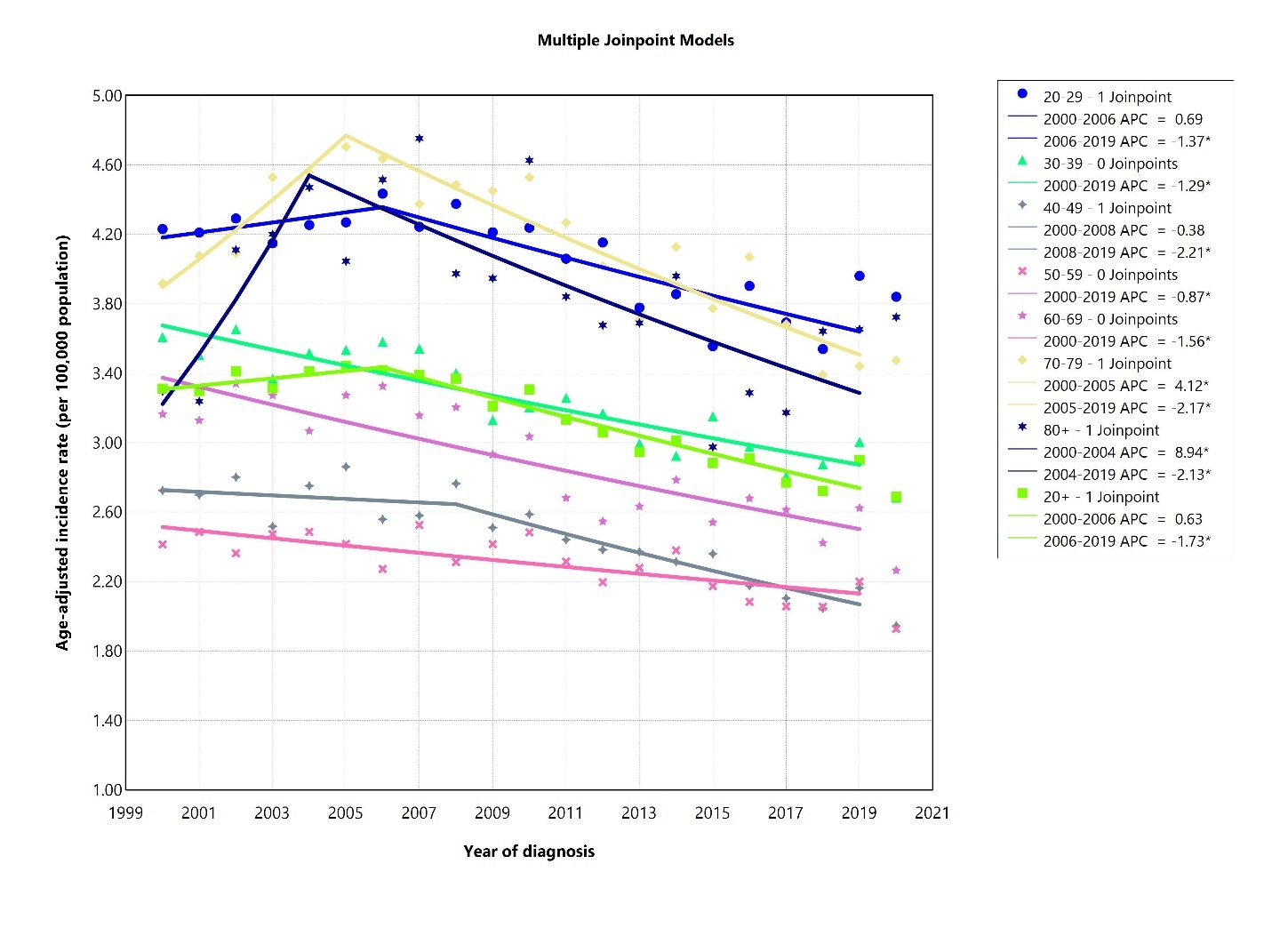


**Figure S2.** Age-adjusted incidence rate of classical Hodgkin lymphoma over 2000-2019 and in 2020 in the United States, by age. APC: annual percent change. * Represent p-value less than 0.05.


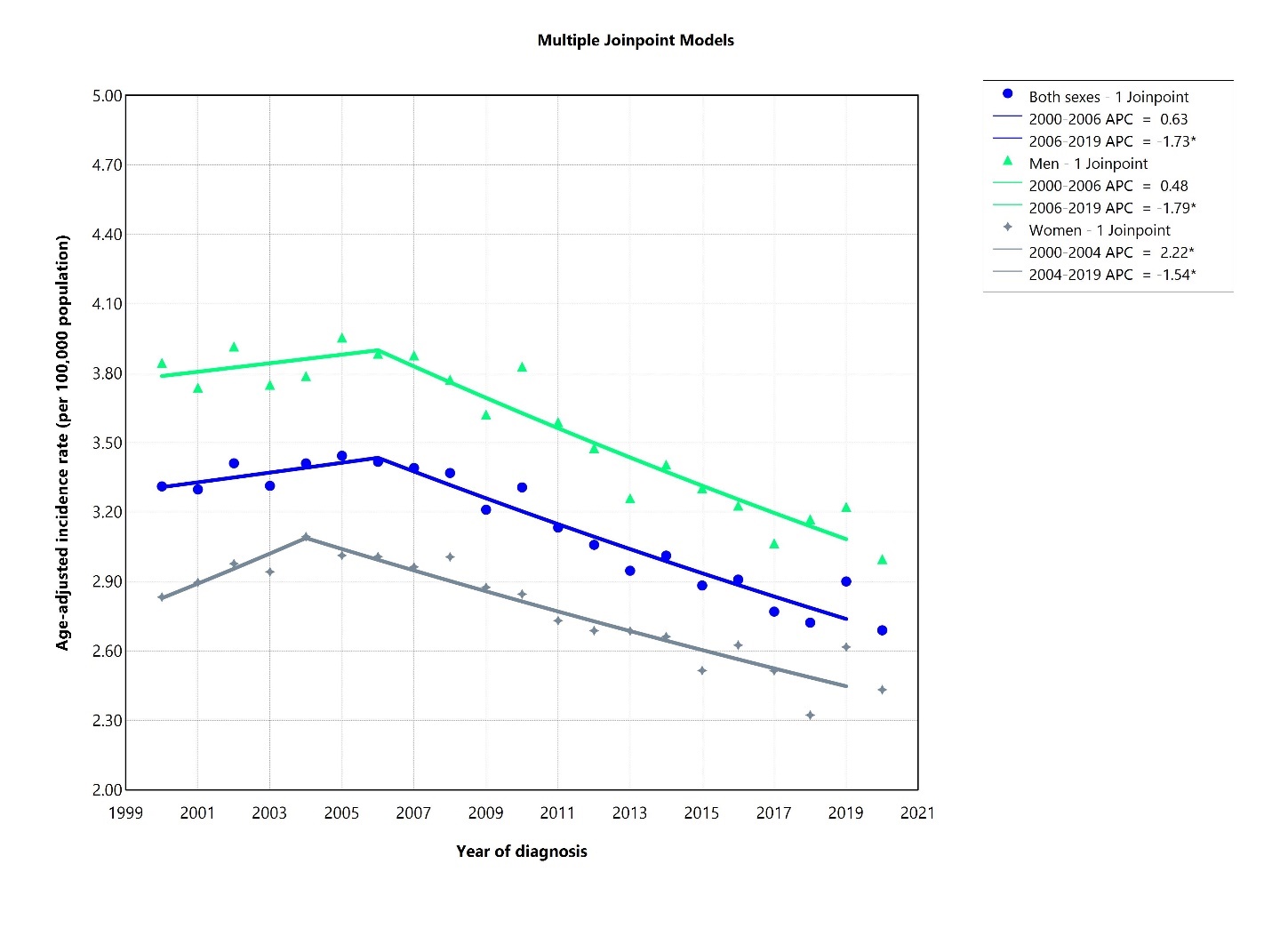


**Figure S3.** Age-adjusted incidence rate of classical Hodgkin lymphoma over 2000-2019 and in 2020 in the United States, by sex. APC: annual percent change. * Represent p-value less than 0.05.


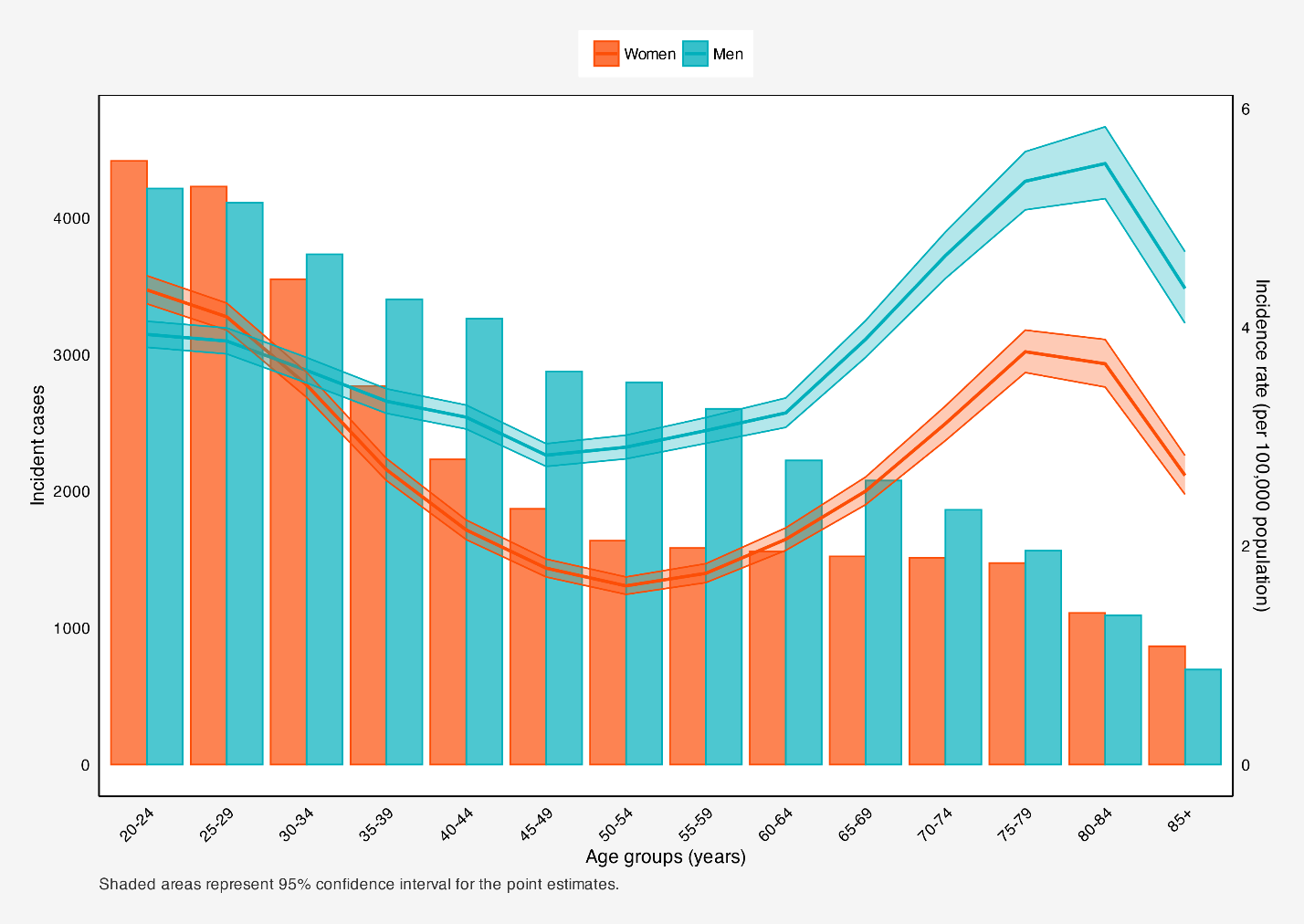


**Figure S4.** Incident cases and incidence rate of classical Hodgkin lymphoma in the United States among males and females in each age group.


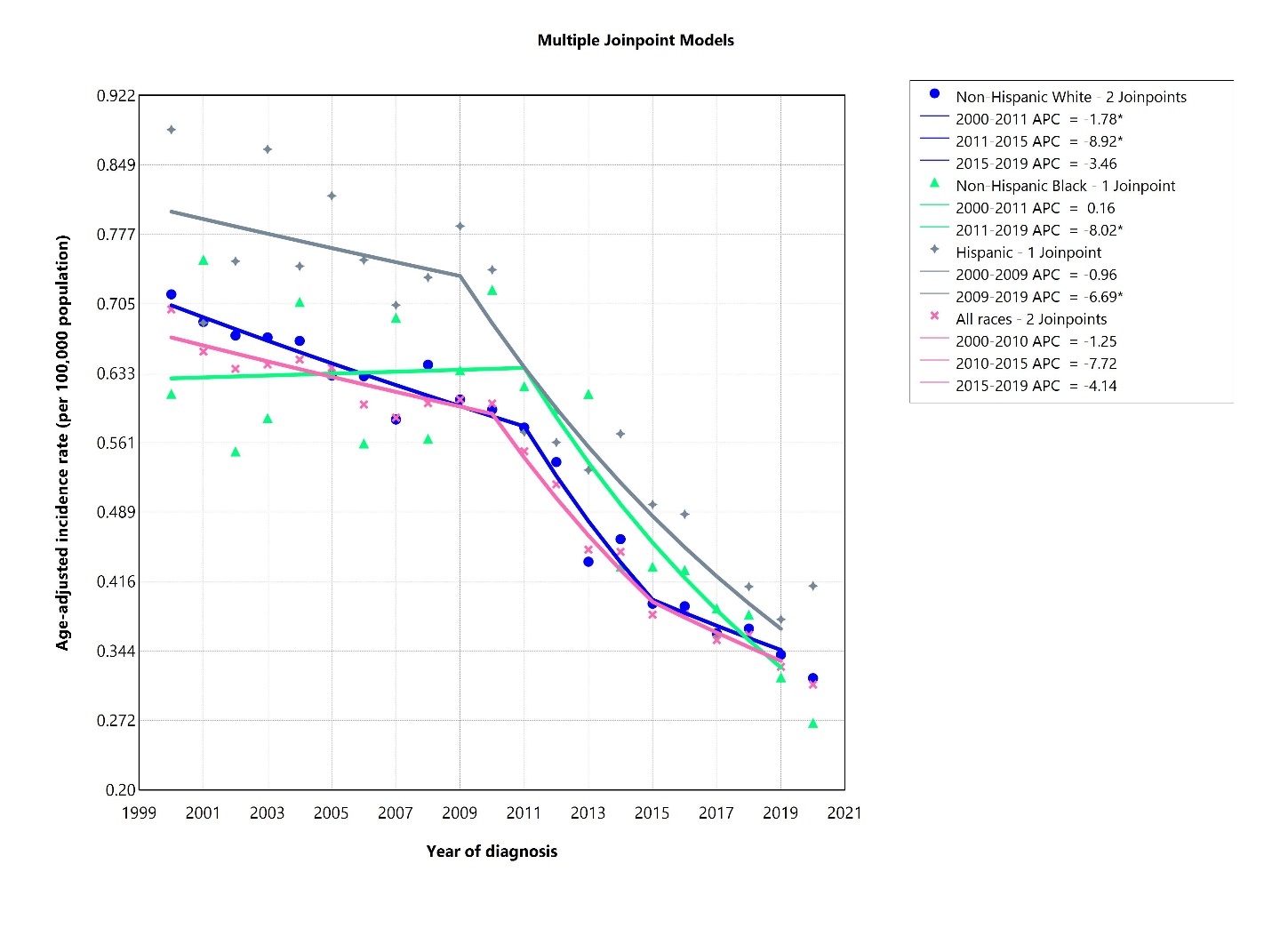


**Figure S5.** Age-adjusted incidence rate of lymphocyte-rich mixed cell lymphocyte depleted Hodgkin lymphoma over 2000-2019 and in 2020 in the United States, by race. APC: annual percent change. * Represent p-value less than 0.05.


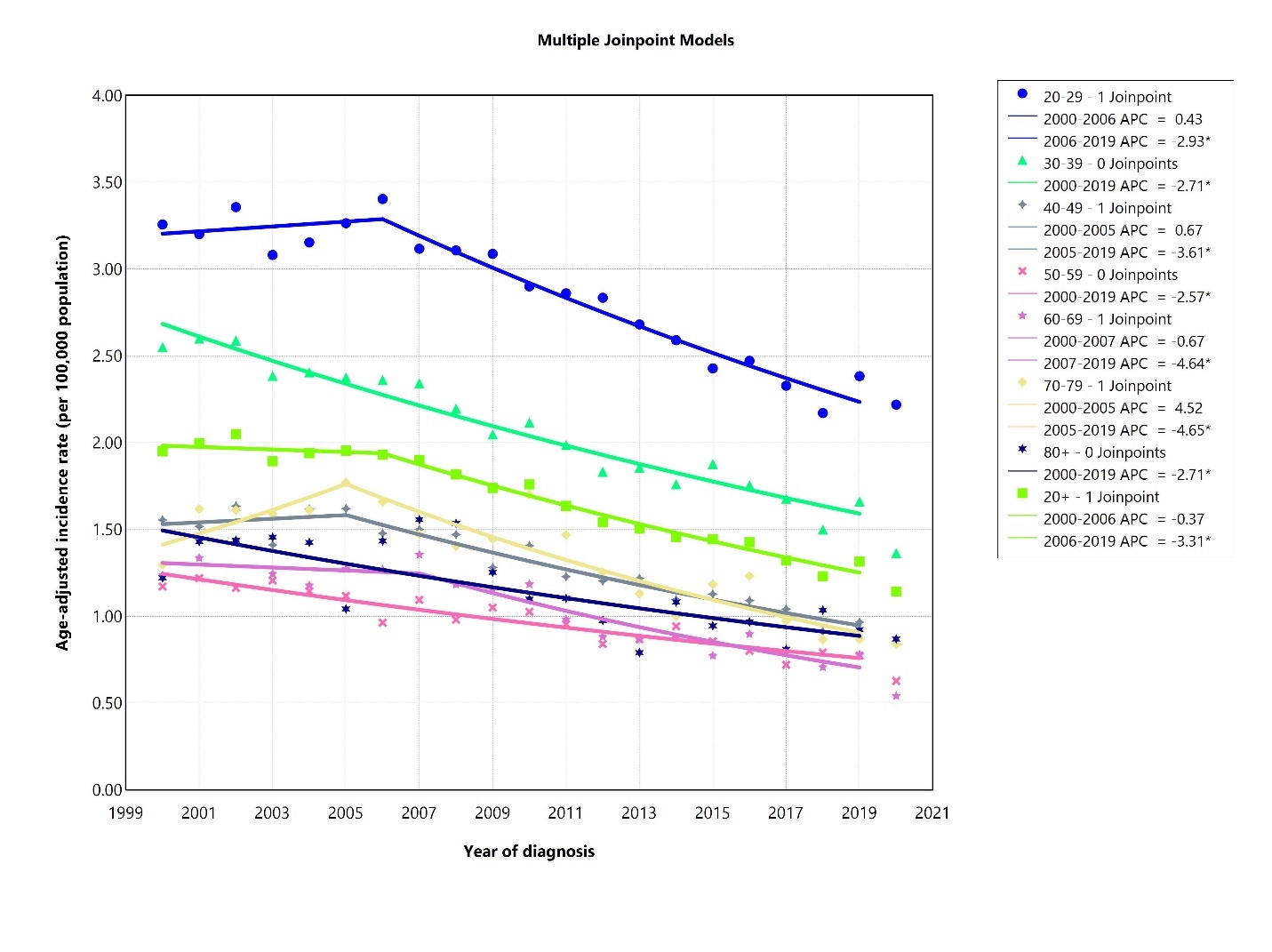


**Figure S6.** Age-adjusted incidence rate of lymphocyte-rich mixed cell lymphocyte depleted Hodgkin lymphoma over 2000-2019 and in 2020 in the United States, by age. APC: annual percent change. * Represent p-value less than 0.05.


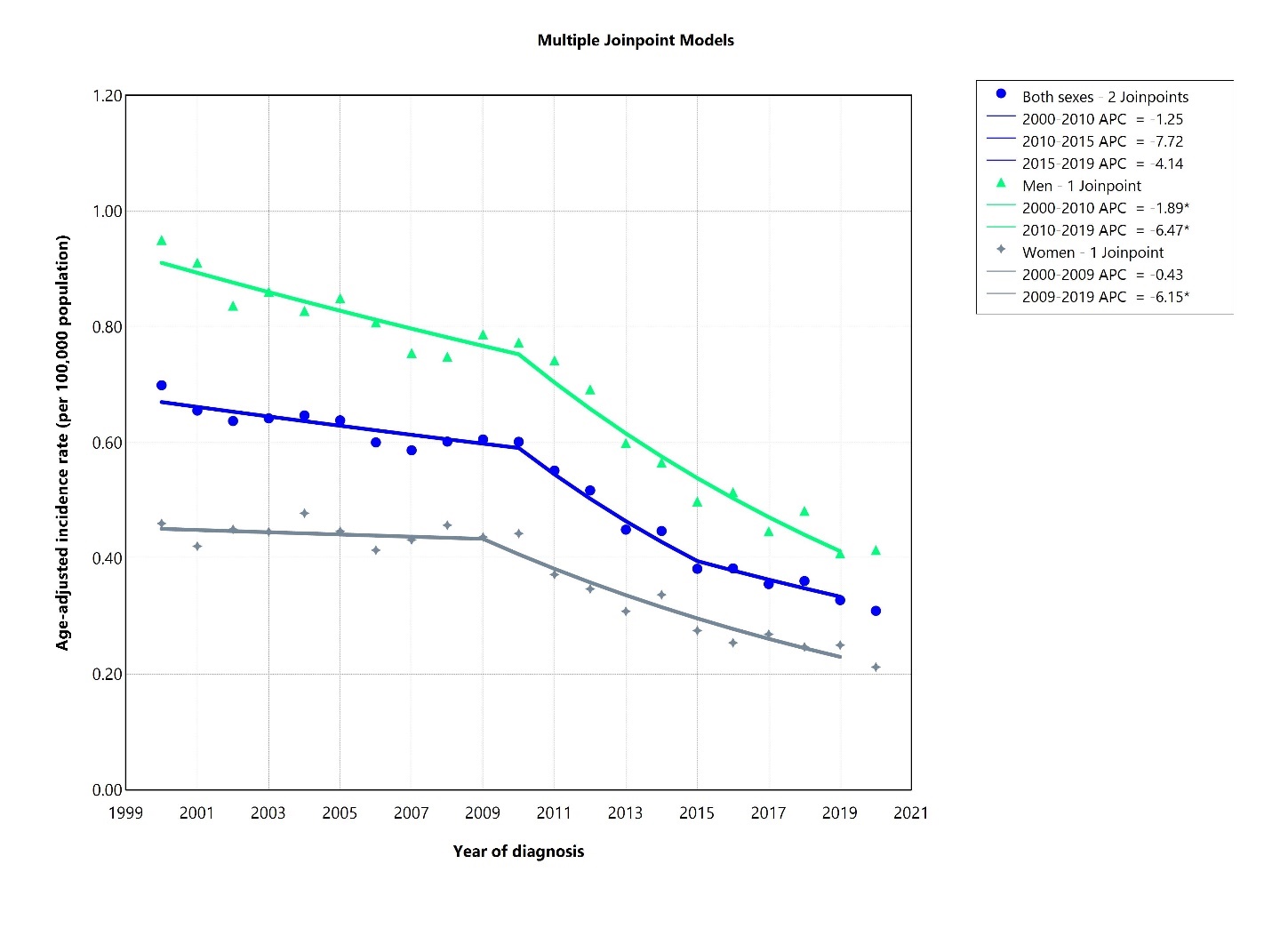


**Figure S7.** Age-adjusted incidence rate of lymphocyte-rich mixed cell lymphocyte depleted Hodgkin lymphoma over 2000-2019 and in 2020 in the United States, by sex. APC: annual percent change. * Represent p-value less than 0.05.


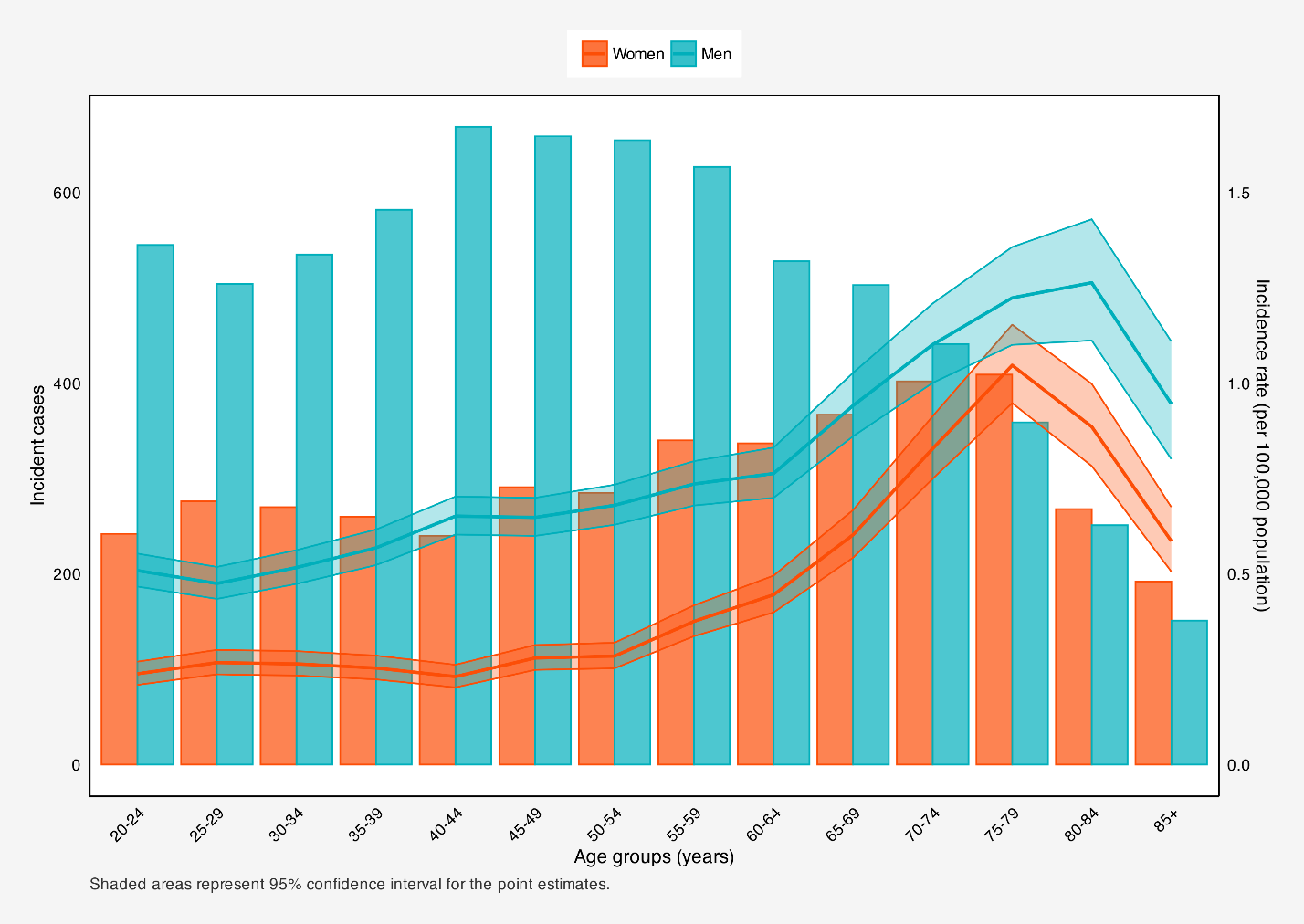


**Figure S8.** Incident cases and incidence rate of lymphocyte-rich mixed cell lymphocyte depleted Hodgkin lymphoma in the United States among males and females in each age group.


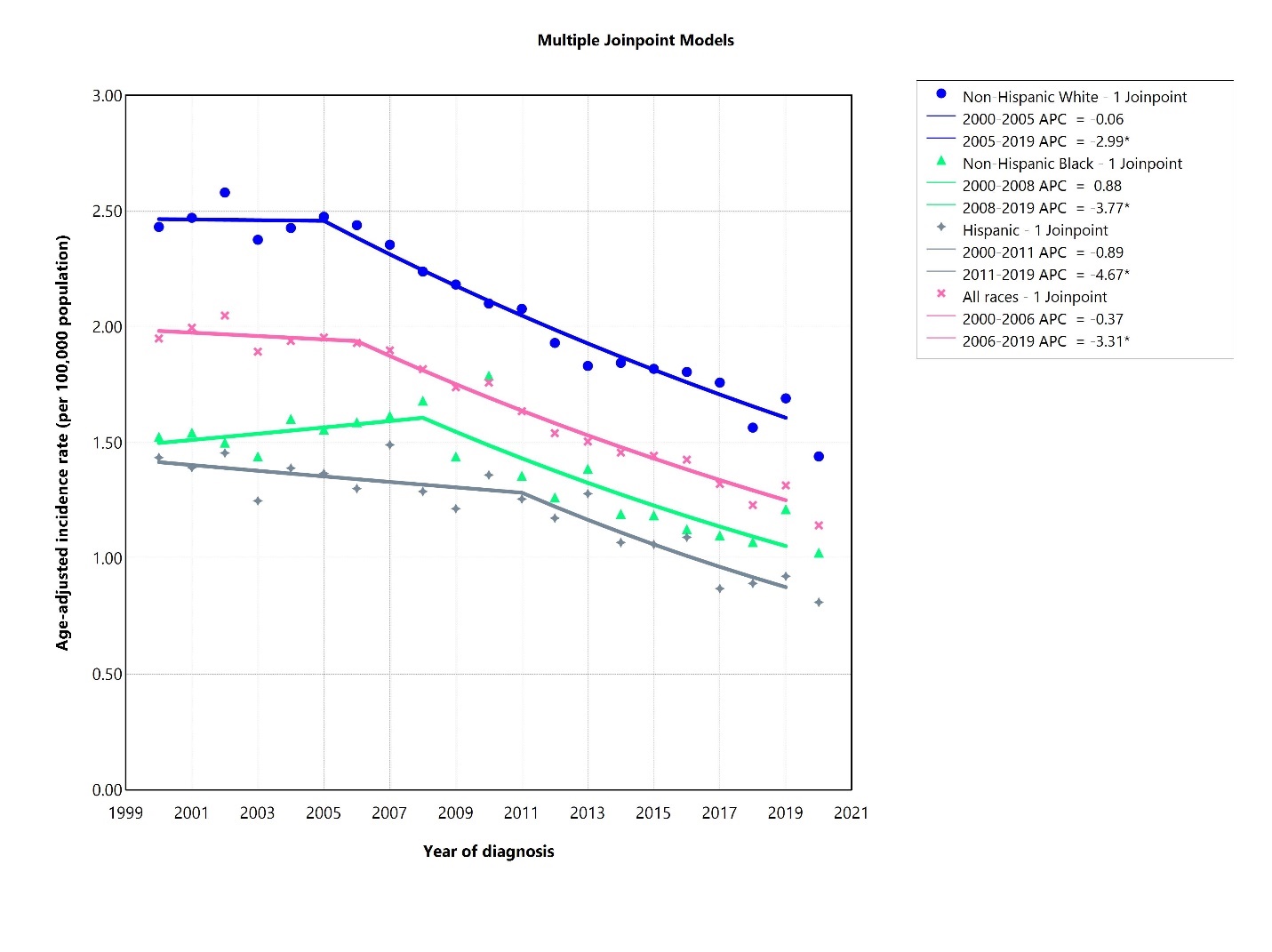


**Figure S9.** Age-adjusted incidence rate of nodular sclerosis Hodgkin lymphoma over 2000-2019 and in 2020 in the United States, by race. APC: annual percent change. * Represent p-value less than 0.05.


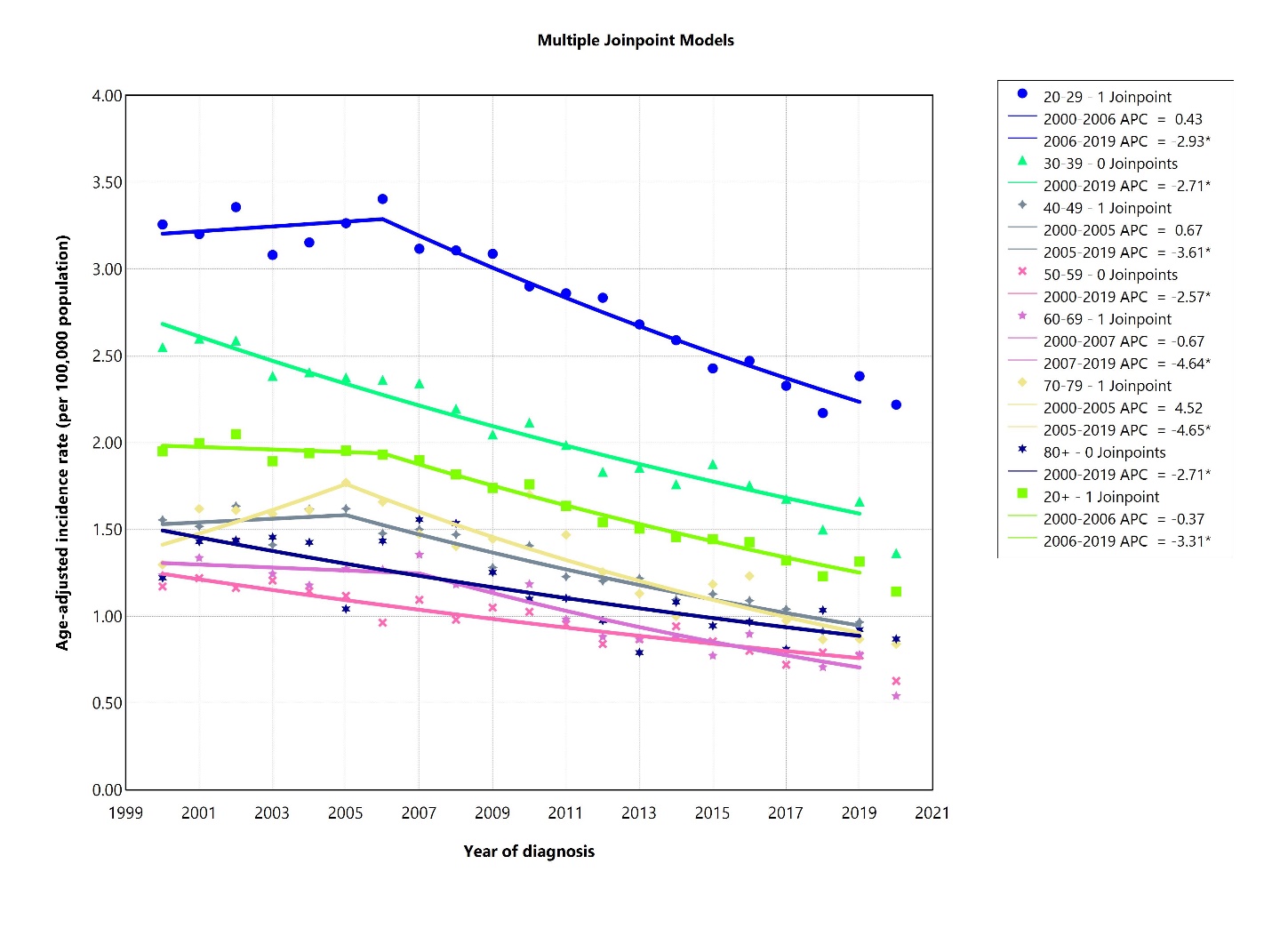


**Figure S10.** Age-adjusted incidence rate of nodular sclerosis Hodgkin lymphoma over 2000-2019 and in 2020 in the United States, by age. APC: annual percent change. * Represent p-value less than 0.05.


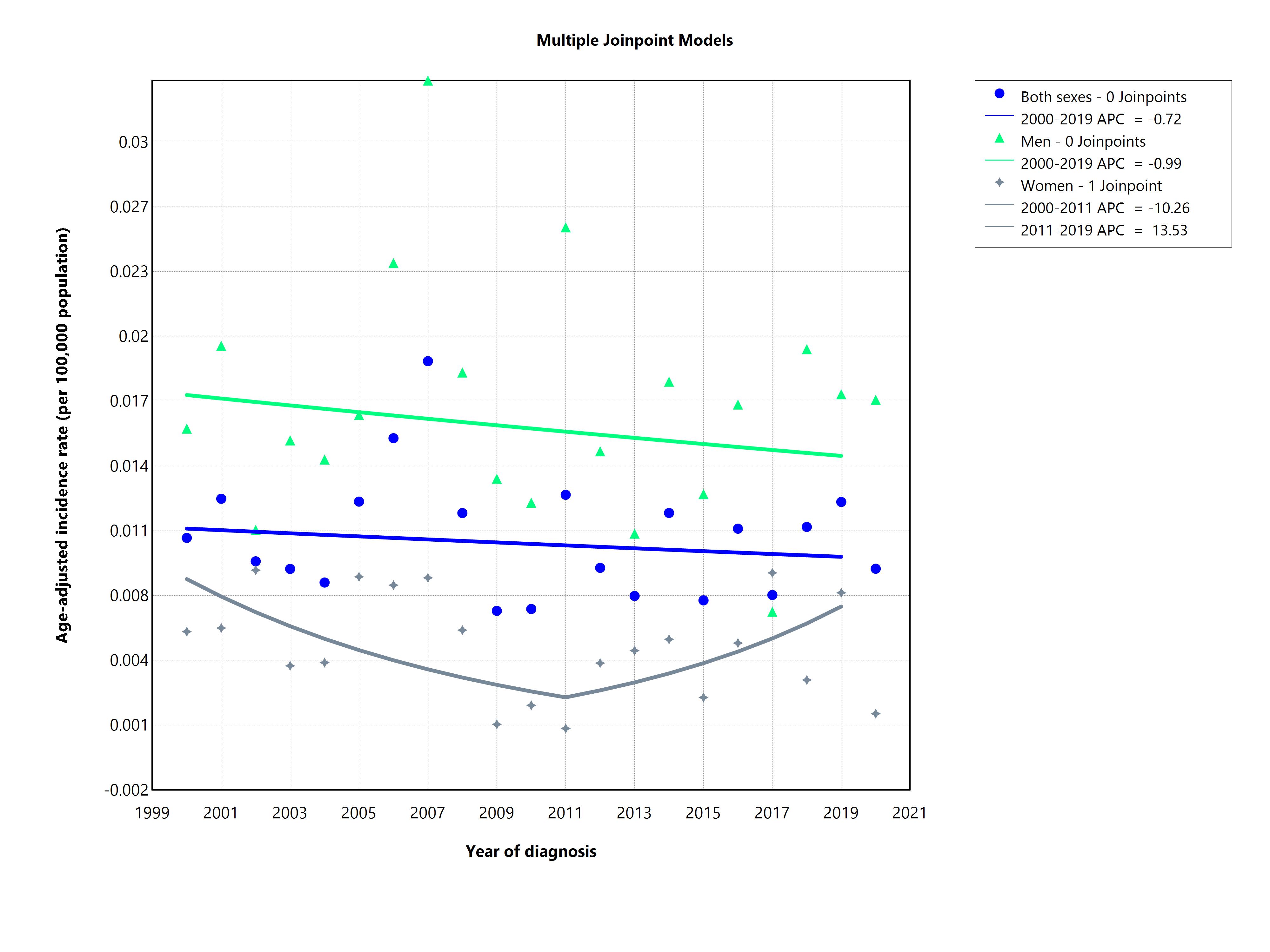


**Figure S11.** Age-adjusted incidence rate of nodular sclerosis Hodgkin lymphoma over 2000-2019 and in 2020 in the United States, by sex. APC: annual percent change.


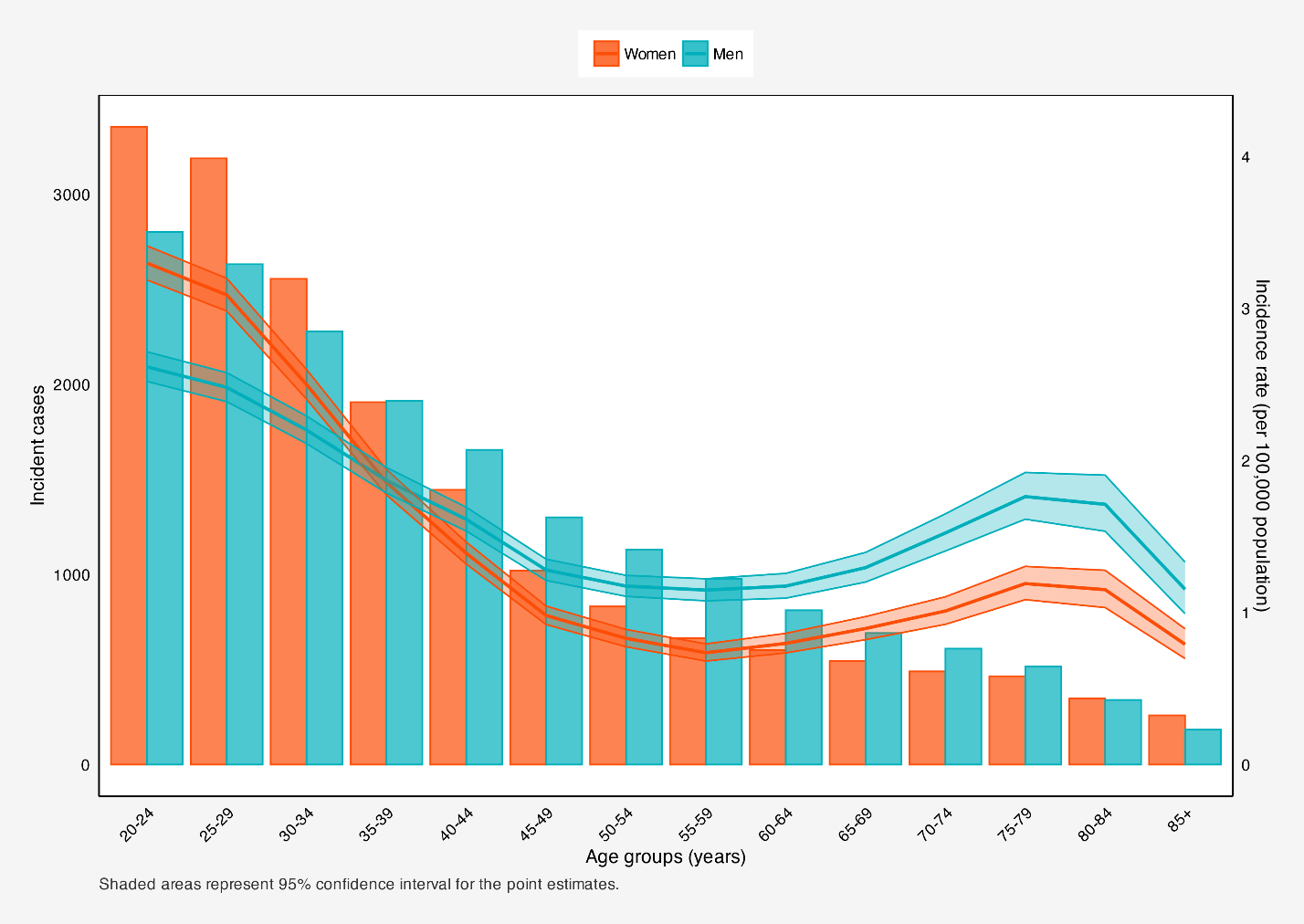


**Figure S12.** Incident cases and incidence rate of nodular sclerosis Hodgkin lymphoma in the United States among males and females in each age group.


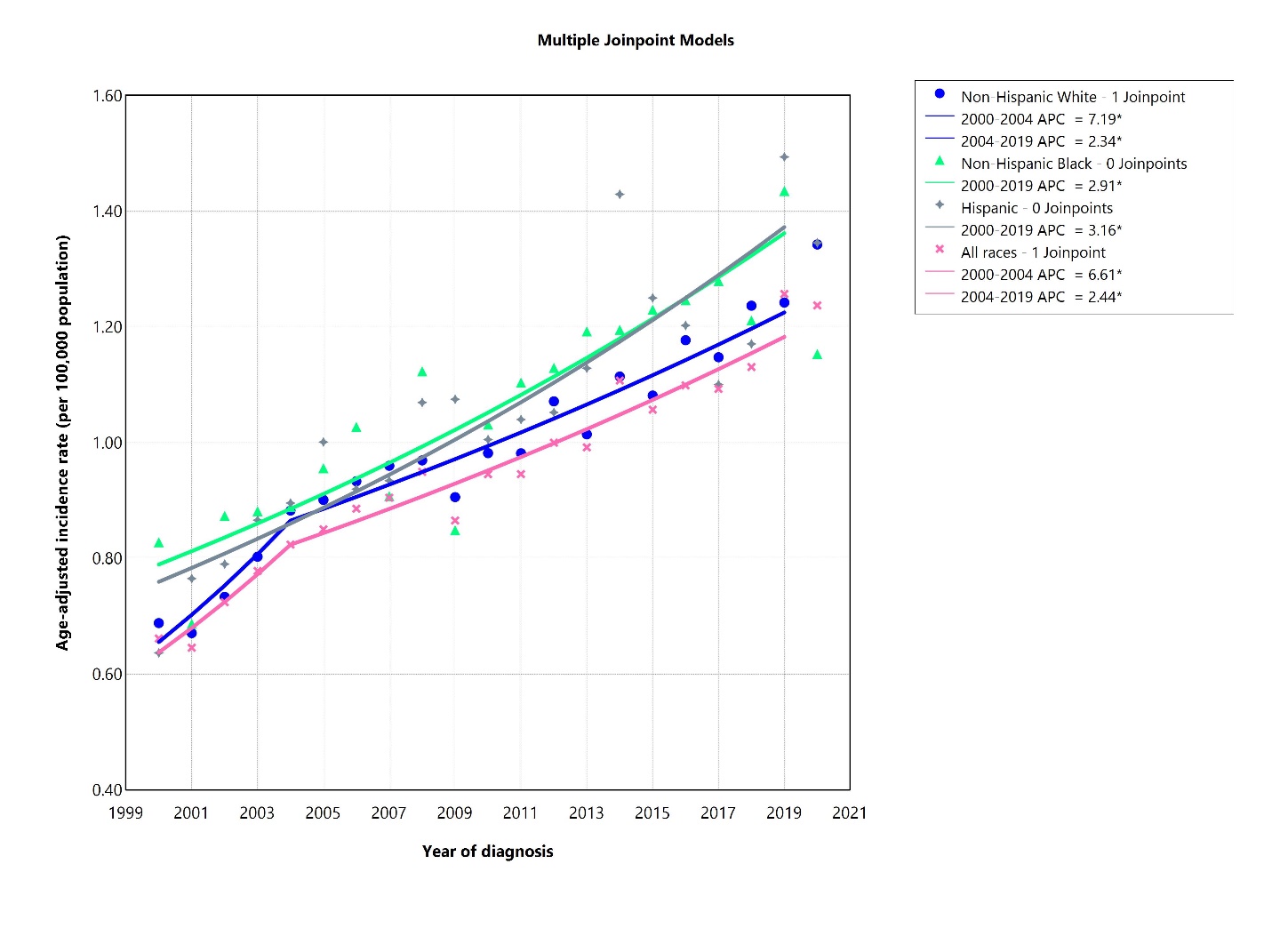


**Figure S13.** Age-adjusted incidence rate of classical Hodgkin lymphoma not otherwise specified (NOS) over 2000-2019 and in 2020 in the United States, by race. APC: annual percent change. * Represent p-value less than 0.05.


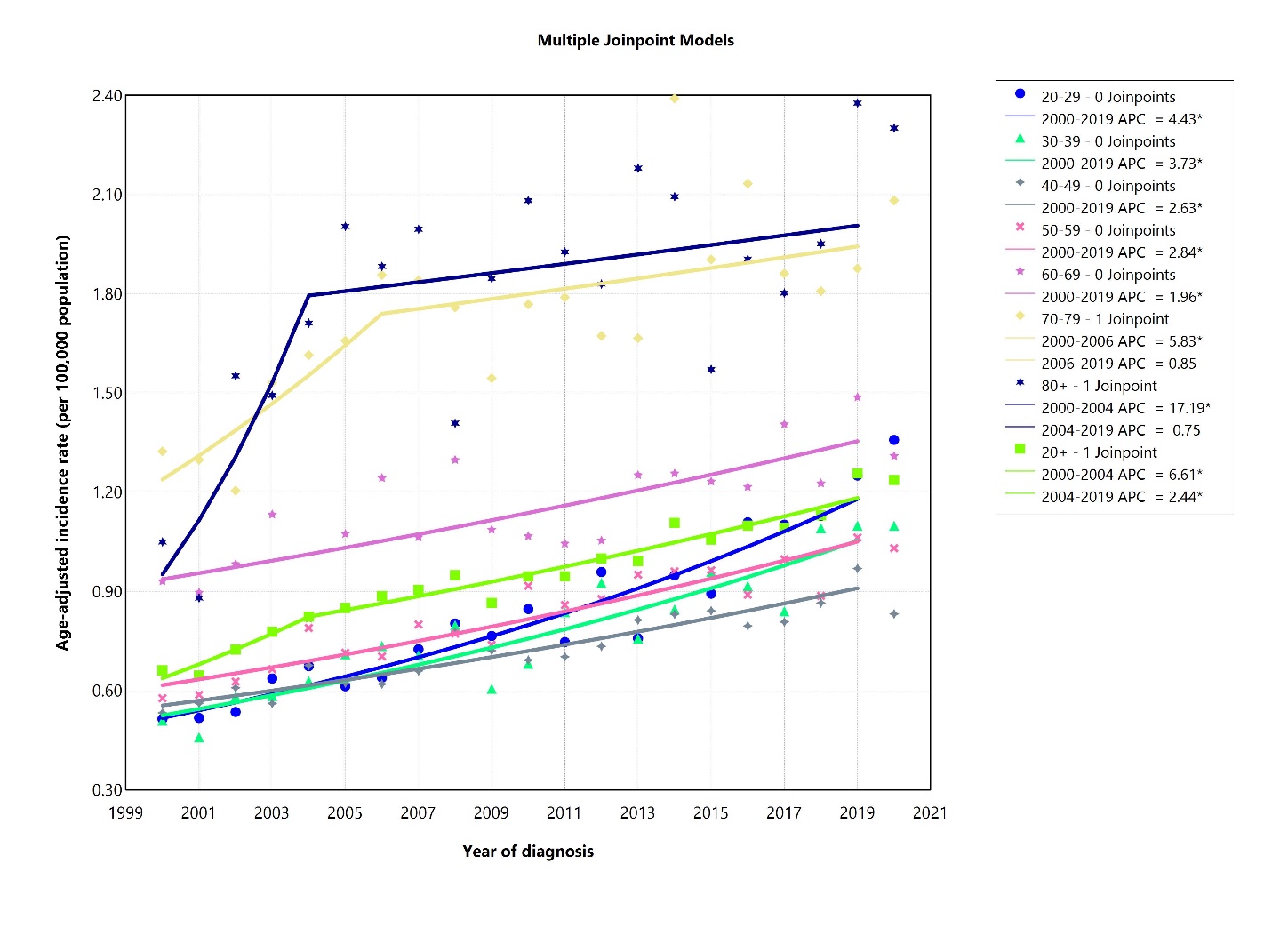


**Figure S14.** Age-adjusted incidence rate of classical Hodgkin lymphoma not otherwise specified (NOS) over 2000-2019 and in 2020 in the United States, by age. APC: annual percent change. * Represent p-value less than 0.05.


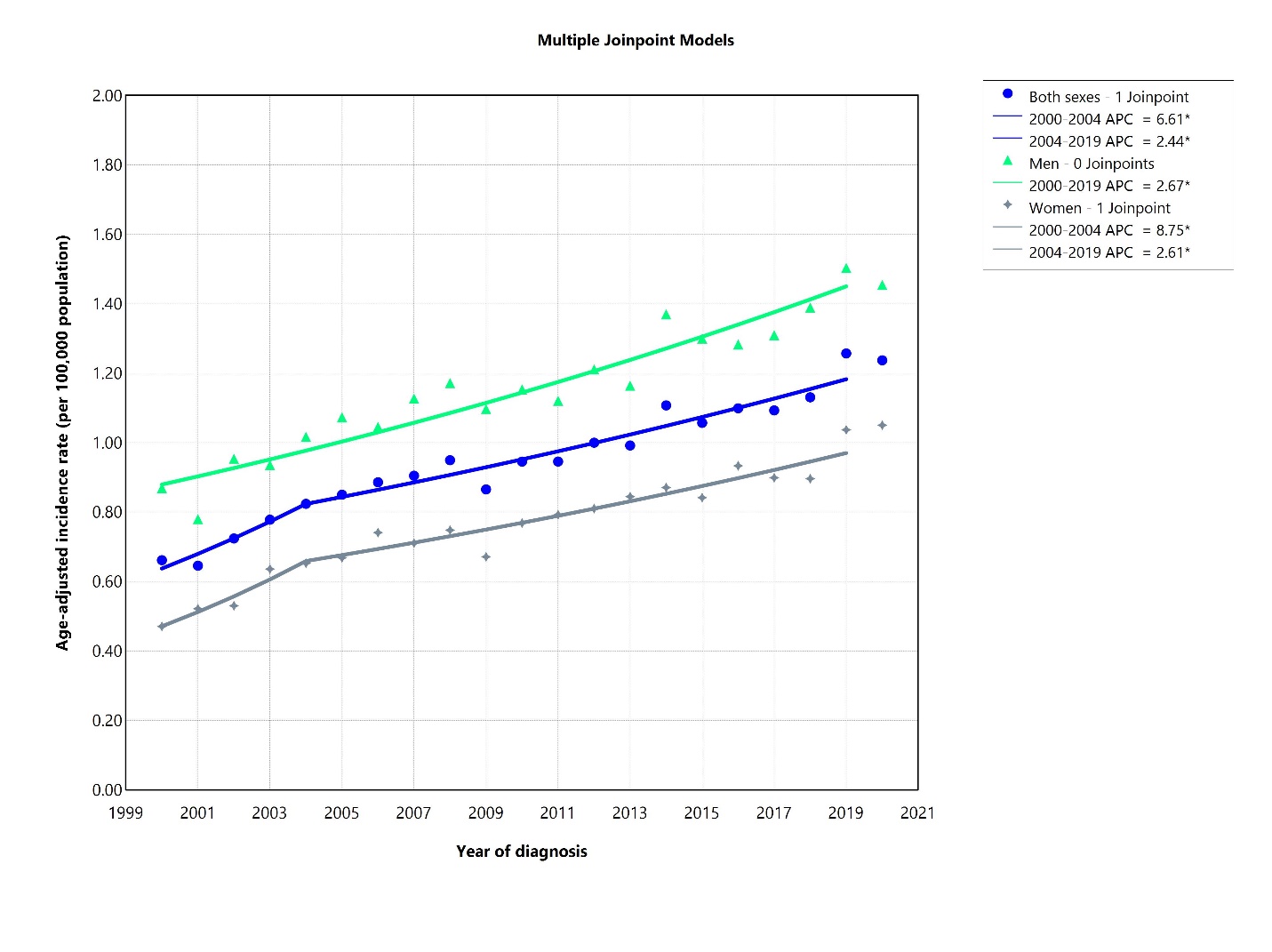


**Figure S15.** Age-adjusted incidence rate of classical Hodgkin lymphoma not otherwise specified (NOS) over 2000-2019 and in 2020 in the United States, by sex. APC: annual percent change. * Represent p-value less than 0.05.


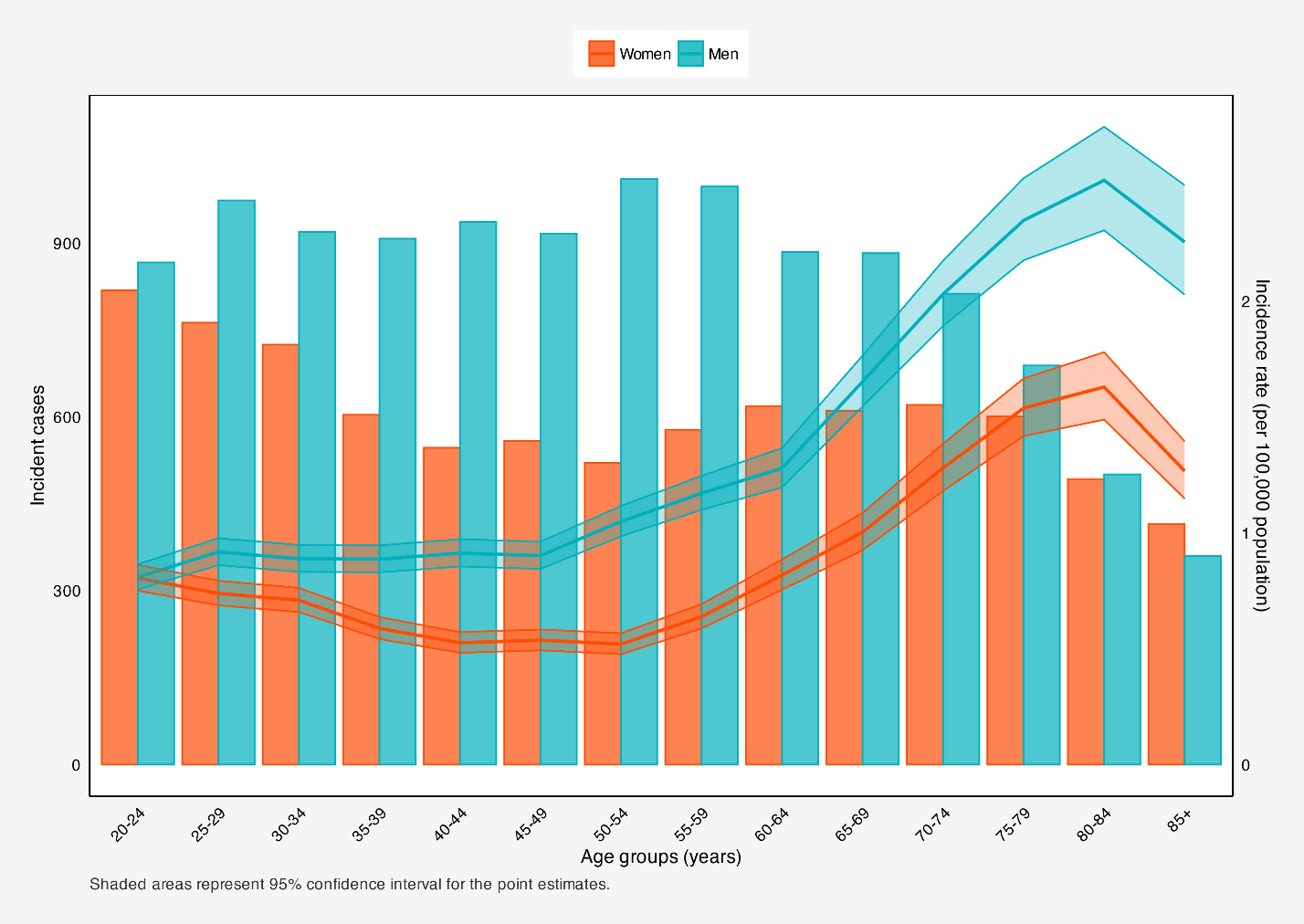


**Figure S16.** Incident cases and incidence rate of classical Hodgkin lymphoma not otherwise specified (NOS) in the United States among males and females in each age group.


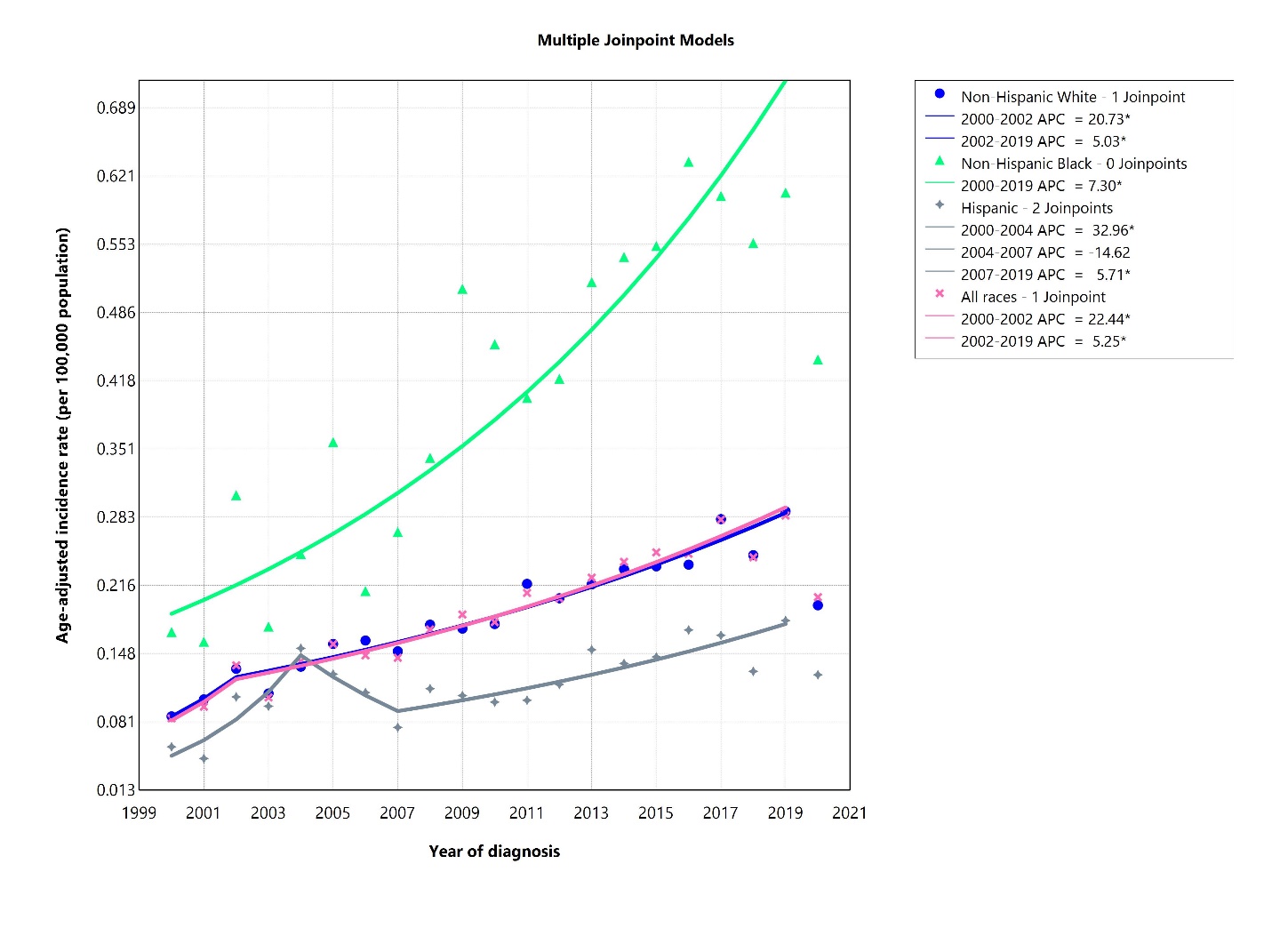


**Figure S17.** Age-adjusted incidence rate of nodular lymphocyte prominent Hodgkin lymphoma over 2000-2019 and in 2020 in the United States, by race. APC: annual percent change. * Represent p-value less than 0.05.


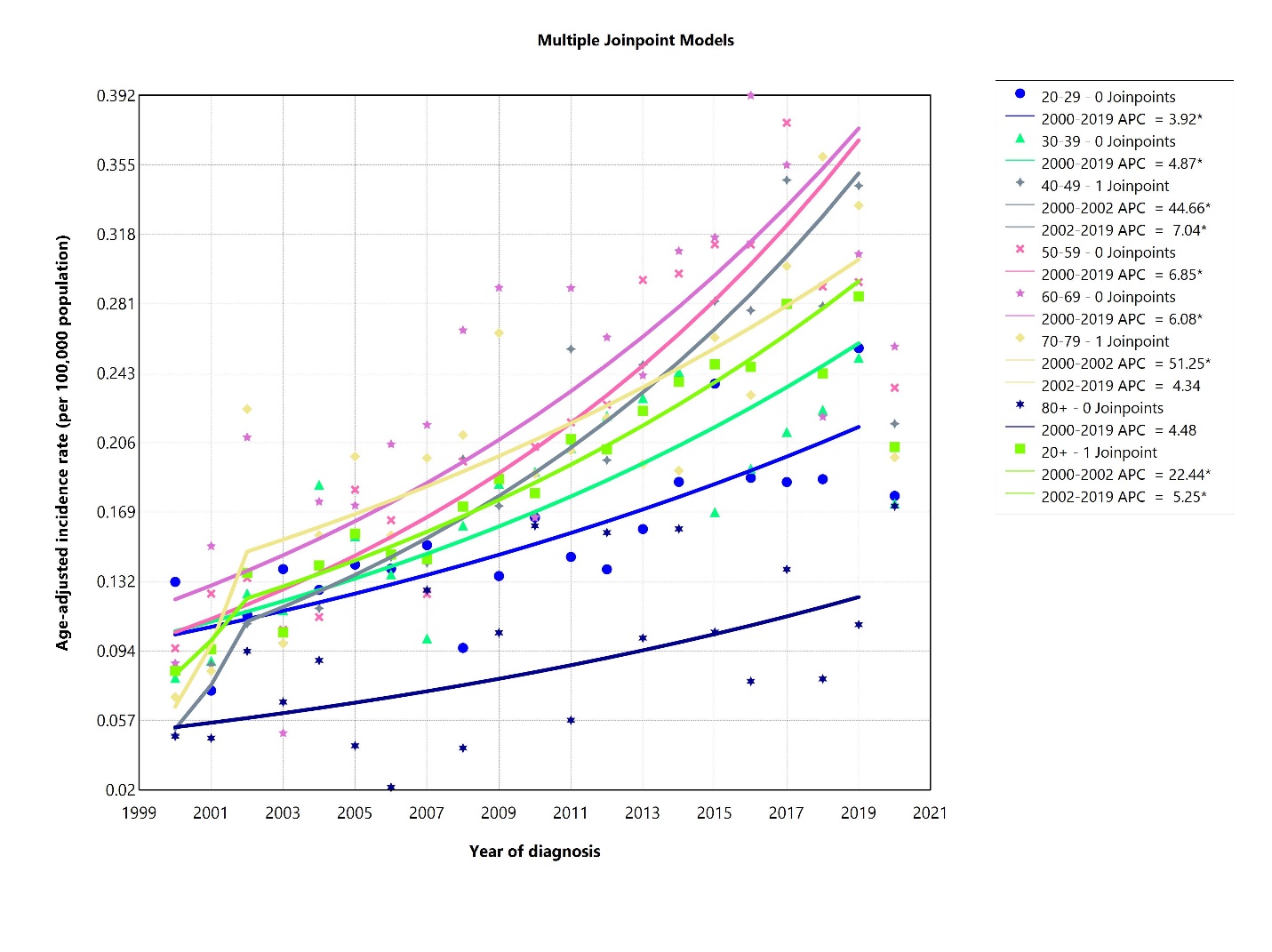


**Figure S18.** Age-adjusted incidence rate of nodular lymphocyte prominent Hodgkin lymphoma over 2000-2019 and in 2020 in the United States, by age. APC: annual percent change. * Represent p-value less than 0.05.


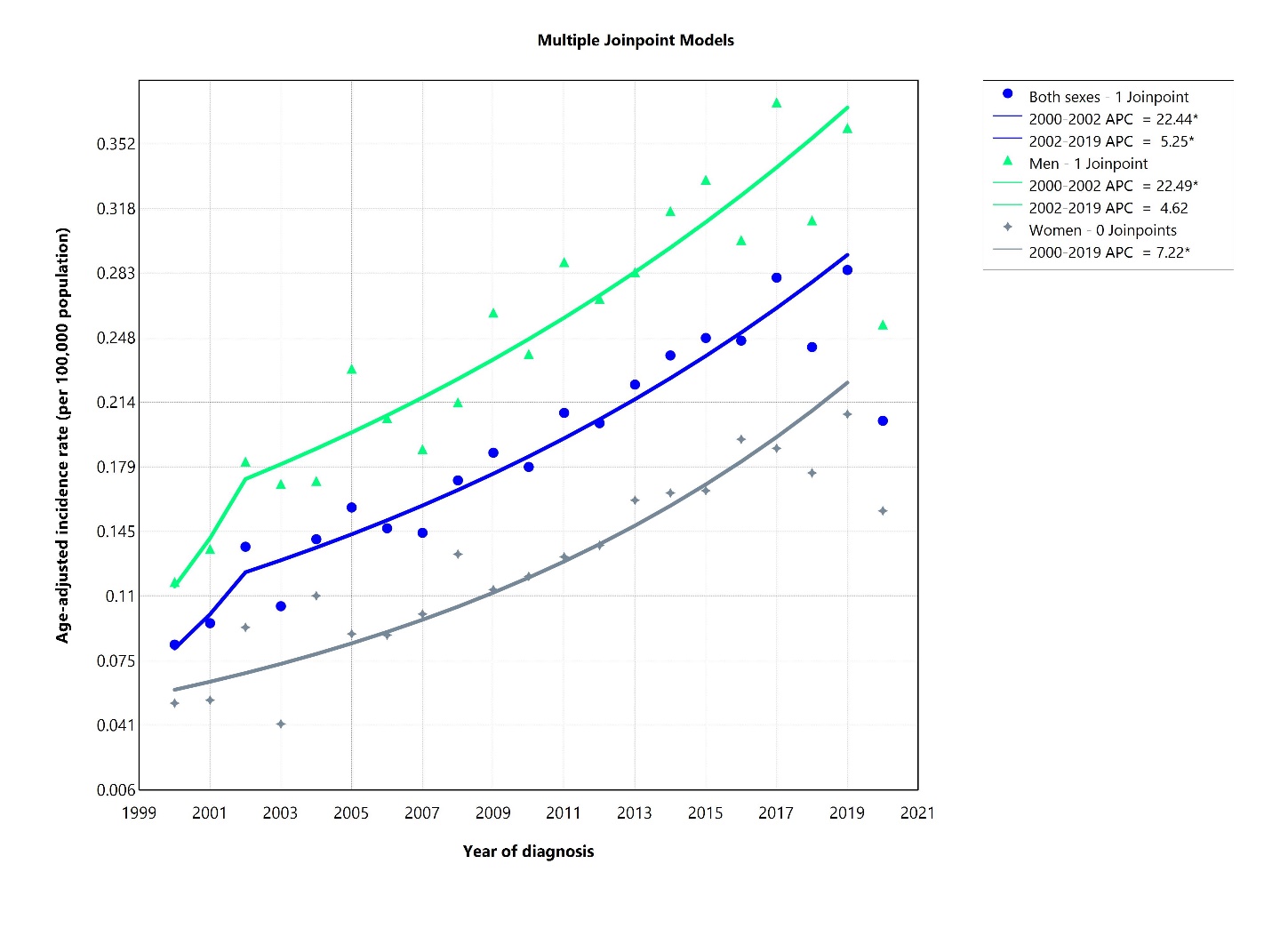


**Figure S19.** Age-adjusted incidence rate of nodular lymphocyte prominent Hodgkin lymphoma over 2000-2019 and in 2020 in the United States, by sex. APC: annual percent change. * Represent p-value less than 0.05.


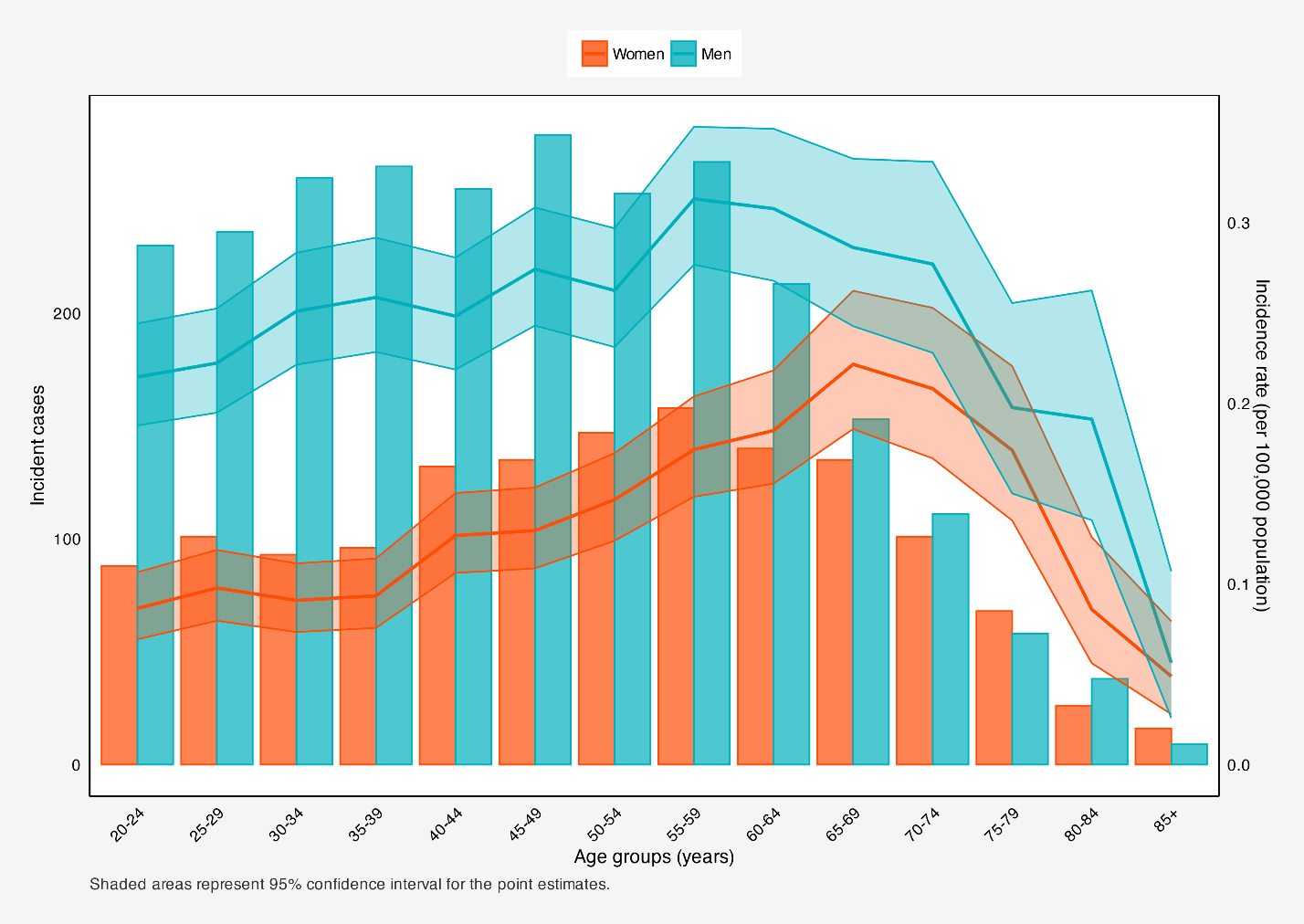


**Figure S20.** Incident cases and incidence rate of nodular lymphocyte prominent Hodgkin lymphoma in the United States among males and females in each age group.
